# Targeting hypothalamic SIK3 to promote weight loss and improve glycemic control in mice

**DOI:** 10.1038/s41467-026-73649-1

**Published:** 2026-06-02

**Authors:** Danise Ann Onda, Chieh-Hsin Yang, Callen Goldsmith, Cait A. Beddows, Rui Qi Teo, Lei Zhang, XiaoZhuo Yuan, Yifei Zhu, Man KS Lee, Ashley J. Ovens, Dingyi Yu, Kei Sakamoto, John W. Scott, Andrew J. Murphy, Noriyuki Tsumaki, Herbert Herzog, Garron T. Dodd, Kim Loh

**Affiliations:** 1 Diabetes and Metabolic Disease, St. Vincent’s Institute of Medical Research, Fitzroy, Victoria, Australia; 2 Department of Anatomy and Physiology, The University of Melbourne, Melbourne, Victoria, Australia; 3 St Vincent’s Center for Applied Medical Research, Sydney, NSW Australia; 4 Division of Immunometabolism, Baker Heart and Diabetes Institute, Melbourne, Australia; 5 Protein Engineering in Immunity & Metabolism, St. Vincent’s Institute of Medical Research, Fitzroy, Victoria, Australia; 6 St. Vincent’s Institute of Medical Research, Fitzroy, Victoria, Australia; 7 Novo Nordisk Foundation Center for Basic Metabolic Research, University of Copenhagen, Copenhagen, Denmark; 8 Drug Discovery Biology, Monash Institute of Pharmaceutical Sciences, Melbourne Victoria, Australia; 9 Division of Immunometabolism, Baker Heart and Diabetes Institute, Melbourne, Australia; Department of Diabetes, Monash University, Melbourne, Australia; Department of Cardiometabolic Health, University of Melbourne, Melbourne, Australia; Department of Medicine, University of Melbourne, Melbourne, Australia; 10 Department of Tissue Biochemistry, Graduate School of Medicine and Frontier Biosciences, Osaka University, Osaka, Japan; 11 Department of Medicine, University of Melbourne, Fitzroy, Victoria, Australia; 12 Mary MacKillop Institute for Health Research, Australian Catholic University, Victoria, Australia

**Keywords:** Hypothalamus, Obesity

## Abstract

Salt-Inducible Kinase 3 (SIK3) has emerged as a key regulator of peripheral metabolism, however its cellular and molecular function in regulating body weight and energy metabolism, particularly in hypothalamic neurons, remains unclear and largely unexplored. Here, we demonstrate that SIK3 expression is elevated in the hypothalamus of obese mice. Inactivation of SIK3 specifically in orexigenic NPY neurons reduces food intake, increases energy expenditure, and enhances white adipose tissue browning, resulting in resistance to high-fat diet-induced obesity in mice. Pharmacological inhibition of SIK3 with the inhibitor GLPG3970 in diet-induced obese mice led to significant reductions in body weight and adiposity, primarily due to decreased energy intake, with notable improvements in metabolic health. These effects are linked to enhanced central leptin and insulin signaling, and the dephosphorylation and nuclear translocation of key transcriptional metabolic regulators, CRTC1 and HDAC5. These findings reveal a previously unidentified SIK3-mediated pathway that promotes positive energy balance and suggests SIK3 inhibition may offer therapeutic potential for treating obesity and metabolic disorders.

## Abstract (web summary)

Obesity results from excess energy intake and reduced energy use. Here, authors show that inhibiting SIK3 suppresses appetite and increases energy expenditure, leading to weight loss.

## Introduction

Obesity affects more than a billion people globally^1^. It poses a major health concern as it is a major risk factor for developing type 2 diabetes, cardiovascular diseases and certain cancers^2–6^. In recent years, a plethora of research has demonstrated that body weight is largely regulated by the coordination between neural and endocrine systems^7–10^. This is achieved by facilitating complex interactions and communication between neuropeptides and hormones^11^. Dysregulation in hormonal signaling, such as leptin and insulin signaling, that regulates energy homeostasis in the hypothalamus, is central to the development of obesity^12–16^. To date, there remains an unmet clinical need for obesity interventions that target adaptive molecular mechanisms to protect against the storage of excess fat mass and to prevent weight regain. Identifying signaling pathways involved in these molecular processes is of major clinical importance.

Key neuronal populations involved in the regulation of body weight and energy homeostasis are Neuropeptide Y (NPY) and Agouti-related peptide (AgRP) expressing neurons, as well as Pro-opiomelanocortin (POMC) expressing neurons, both situated within the arcuate nucleus of the hypothalamus (ARC)^17–19^. These functionally opposing neuronal populations have the specialized capability to sense circulating signals from the periphery, which include but are not limited to, adipose, nutrient, gastrointestinal and pancreatic signals^7,20,21^. Upon receiving these signals, NPY/AgRP and POMC neurons function to adjust food intake and energy expenditure in accordance with homeostatic needs^22–25^. Therefore, identifying the endogenous regulators within these neurons that control body weight and energy balance is critical for understanding the pathology of obesity.

The Salt-Inducible-Kinases (SIKs) belong to the AMPK-related kinase subfamily of serine/threonine protein kinases and consist of 3 isoforms: SIK1, SIK2, and SIK3^26–28^. Previous studies have indicated that the genes encoding SIK isoforms are evolutionarily conserved and present across various species including *Drosophila*, mice and humans^29–31^. Structurally, all SIK isoforms contain a highly homologous N-terminal kinase domain (KD) that, like all 13 AMPK-related kinases, can be phosphorylated and activated by the upstream kinase, Liver Kinase B1 (LKB1)^32,33^. Upon activation, SIKs regulate gene expression by phosphorylating several key transcriptional regulators, such as class IIa histone deacetylases (HDACs) and cAMP response element-binding protein-regulated transcription coactivators (CRTCs)^29,34–36^. Conversely, SIKs are inhibited in a cyclic AMP/protein kinase A (cAMP/PKA)-dependent manner, likely by phosphorylation-induced 14-3-3 binding, which in turn reduces substrate availability^29,34,35^. In addition, changes in nutrient and hormonal cues can also influence the expression and activity of SIKs. For instance, insulin stimulation or chronic exposure to high glucose levels increases SIK protein abundance and kinase activity^37–42^.

The SIK family has been implicated in the regulation of various physiological processes, including bone development, circadian cycle and immune function^31,43–47^. Thus far, SIK1 and SIK2 are the most well-studied SIKs, whereas the function and metabolic regulation of SIK3 remain largely undetermined. This is due to the lack of suitable models, since global SIK3 knockout mice are associated with partial lethality, skeletal abnormalities and dwarfism and thus do not represent an ideal model for physiological/metabolic study^48,49^. In *Drosophila* and *C. elegans*, however, SIK3 has been shown to play an essential role in fat storage and mobilization. Interestingly, although SIK3 is ubiquitously expressed in mice^29,34,35,50^, SIK3 has the highest level of expression in the brain and accounts for ~ 80% of total SIK enzymatic activity ( ~ 5% for SIK1 and ~ 15% for SIK2)^46^. *SIK3* was identified as a novel gene associated with dyslipidaemia and obesity in the Amerindian population^51^. Despite the high levels of expression within the hypothalamus, the role of SIK3 in regulating body weight and energy homeostasis is yet to be investigated. Here, we identify a previously unknown signaling pathway linking SIK3 to the development of obesity and show inhibition of SIK3 in orexigenic neurons protects mice against diet-induced weight gain. Our preclinical findings, utilizing an orally bioavailable, highly potent SIK2/3 inhibitor with an acceptable safety profile in humans, reveal SIK3 as a therapeutic target for treating obesity and its associated comorbidities.

## RESULTS

### Hypothalamic SIK3 expression is elevated in obesity

**Fig. 1:**
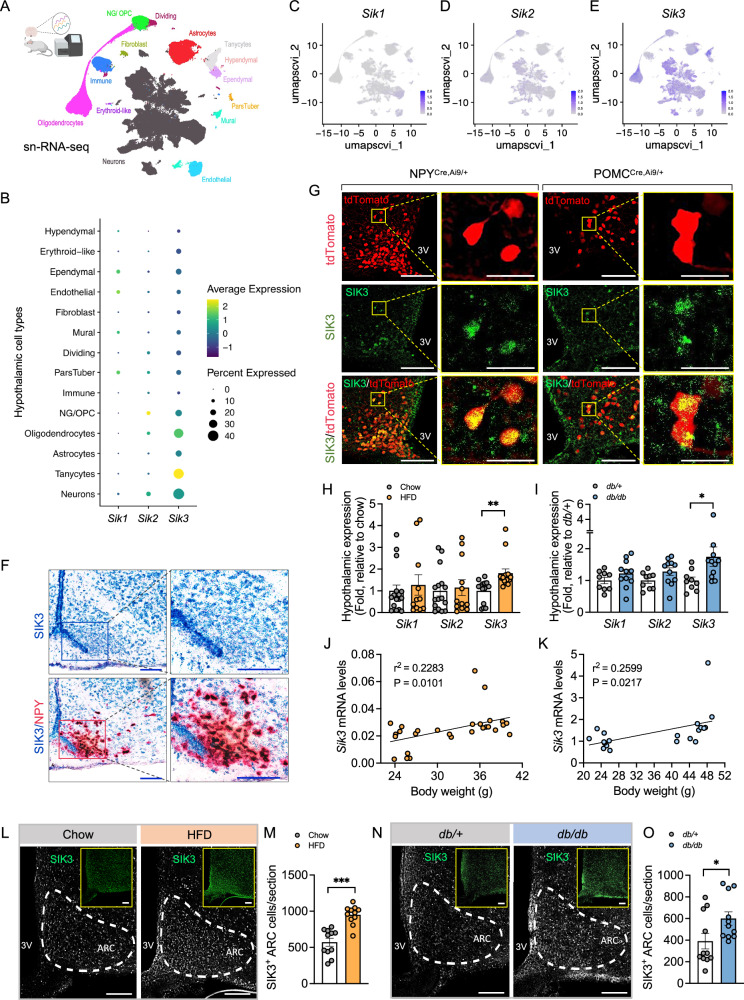
Identification of SIK3 positive cells in the hypothalamus of the murine brain. **A** UMAP visualization of HypoMap, colored by major cell types. RNA-sequencing method diagram: Created in BioRender. Onda, D. A. (2026) https://BioRender.com/mv2x6id. **B** Dot plot visualization of *Siks* expression in a hypothalamic-cell type specific manner. **C**–**E** UMAP plot analysis of *Sik1*, *Sik2*, and *Sik3* within different neuronal subtype populations in the hypothalamus. Each dot is colored according to the expression level. **F** Representative images from double in situ hybridization using RNA Scope with chromogenic visualization of *Sik3* in blue and *Npy* in red in the ARC of wild-type HFD-fed mice. Scale bar, 100 µm. *n* = 5 biologically independent samples. **G** Immunofluorescence staining of NPY^Cre/+,Ai9/+^ and POMC^Cre/+,Ai9/+^ mice showing co-localization of SIK3 in green with NPY or POMC neurons in tdTomato red, on a protein level. Scale bar, 100 µm and 25 µm (zoomed-in images). *n* = 3 biologically independent samples per group. **H** mRNA levels of *Sik1*, *Sik2* and *Sik3* in the hypothalamus of chow and HFD-fed mice (normalized to *Gapdh* and presented as fold change relative to chow for each gene). (chow: *n* = 14–16 biologically independent samples; HFD:* n* = 11–14 biologically independent samples). Data are mean ± s.e.m. **P = 0.002, by two-tailed Student’s *t* test. **I** mRNA levels *of Sik1, Sik2* and *Sik3* in the hypothalamus of lean *db/+* and obese *db/db* mice (normalized to *Gapdh* and presented as fold change relative to *db/+* for each gene). (*db/+*: *n* = 9 biologically independent samples; *db/db*: *n* = 11 biologically independent samples). Data are mean ± s.e.m. *P = 0.038, by two-tailed Student’s *t* test. **J** Correlation between mRNA levels of *Sik3* against changes in body weight in chow and HFD-fed mice. *n* = 28 biologically independent samples. Data are mean ± s.e.m. *P = 0.010, by simple linear regression. **K** Correlation between mRNA levels of *Sik3* against changes in body weight in lean *db/+* and obese *db/db* mice. *n* = 20 biologically independent samples. Data are mean ± s.e.m. *P = 0.021, by simple linear regression. **L**, **M** Immunostaining of SIK3-positive cells in the ARC of chow and HFD-fed mice. Scale bar, 100 µm. *n* = 11 biologically independent samples per group. Data are mean ± s.e.m. ***P < 0.001, by two-tailed Student’s *t* test. **N**, **O** Immunostaining of SIK3-positive cells in the ARC of lean *db/+* and obese *db/db* mice. Scale bar, 100 µm. *n* = 11 biologically independent samples per group. Data are mean ± s.e.m. *P = 0.039, by two-tailed Student’s *t* test. Data are from at least two independent experiments. Source data are provided as a Source Data file.

Previous studies have reported that SIK3 is highly expressed in the brain^46^, however the anatomical or cellular correlates are unknown. Through interrogation of the HypoMap single-nucleus RNA-seq database (Fig. 1A)^52^, we identified that *Sik3* is most highly expressed in neurons, followed by tanycytes and oligodendrocytes (Fig. 1B). These data also show greater *Sik3* expression compared to *Sik1* and *Sik2* across these cell types (Fig. 1B–E). Importantly, we found *Sik3* is also expressed in neuronal cells expressing *Sim1*, *Nr5a1*, and *Tbx3*, which are established markers for neurons within the PVN, VMH and ARC, respectively (Supplementary Fig. S1A–D). ARC NPY/AgRP and POMC-expressing neurons are the two predominant first-order neuronal populations through which afferent humoral and nutrient signaling, particularly leptin and insulin, drive the central control of metabolism^17,21,53–55^. To validate the snRNA-seq datasets, we examined the co-expression of *Npy* (red) and *Sik3* (blue) mRNA by chromogenic RNA-scope in the ARC. We found *Sik3* indeed colocalizes with *Npy* mRNA (Fig. 1F). To further confirm the co-existence of SIK3 in NPY and POMC neurons at the protein level, we utilized our NPY^cre/+,Ai9/+^ and POMC^cre/+,Ai9/+^ Cre reporter models, which expresses the fluorescence marker tdTomato under the control of the NPY and POMC promoter, respectively. We performed immunostaining for SIK3 and observed significant co-localization of ARC SIK3-positive cells (green) with both NPY and POMC neurons (tdTomato red) (Fig. 1G). Immunostaining analysis in the ARC revealed that approximately 24% of hypothalamic SIK3⁺ cells co-express NPY and about 18% co-express POMC, supporting the notion that SIK3 is enriched in key neurons regulating energy homeostasis (Supplementary Fig. S1E). Collectively, these observations demonstrate that NPY and POMC neurons within the ARC express SIK3.

*SIK3* was previously identified as a novel risk gene associated with dyslipidaemia and obesity^51^. To establish the role of the SIK3 signaling pathway in obesity development, we first assessed whole hypothalamic *Sik3* mRNA expression in both diet-induced and genetically induced obese mouse models. Hypothalamic *Sik3* mRNA levels were significantly increased after 10 weeks of high-fat diet (HFD) feeding in mice compared to their lean chow-fed counterparts (Fig. 1H). Consistently, we observed similar increases in hypothalamic *Sik3* mRNA levels in 12-week-old genetically induced leptin receptor-deficient (*db/db*) mice when compared to their lean (*db/+*) counterparts (Fig. 1I). In contrast, both hypothalamic *Sik1* and *Sik2* levels remained unchanged across both obesogenic mouse models (Fig. 1H, I). Correlation analysis revealed that increases in *Sik3* mRNA expression were significantly positively correlated with increases in body weight in obese mice (Fig. 1J, K), while no correlation was noted between *Sik1* or *Sik2* expression and changes in body weight (Supplementary Fig. S1F–I). To assess SIK3 activity in obesity, we performed immunoblotting of whole hypothalamic lysates from lean chow and HFD-fed mice.  Antibodies were first validated in SK-N-SH-SY5Y cells over expressing SIK3 (Supplementary Fig. S1J). Quantification revealed a marked increase in both phosphorylated SIK3 and total SIK3 protein levels in HFD-fed mice compared to lean controls. (Supplementary Fig. S1K–M). To determine the specific area of the hypothalamus where SIK3 is upregulated, we conducted immunofluorescent staining and observed a significant increase in SIK3 levels in the ARC of HFD-fed (Fig. 1L, M) and db*/db* (Fig. 1N, O) mice. This increase was also evident in the paraventricular hypothalamic nucleus (PVN) (Supplementary Fig. S1N–Q). Collectively, these results suggest that increased hypothalamic SIK3 expression correlates to the development of obesity.

### Selective deletion of SIK3 in NPY neurons attenuates diet-induced obesity

We demonstrate that SIK3 is expressed in key metabolically relevant neurons, NPY and POMC neurons in the ARC. To understand the role of SIK3 specifically in the orexigenic NPY neurons or anorexigenic POMC neurons, we crossed SIK3^lox/lox^ mice^56^ with mice expressing Cre recombinase under the control of either the NPY promoter (NPY^cre/+^ mice)^57^ or POMC promoter (POMC^IRES,cre/+^ mice)^58^ to generate NPY neuron-specific SIK3-deficient (NPY^cre/+^,SIK3^lox/lox^) or POMC neuron-specific SIK3-deficient (POMC^cre/+^,SIK3^lox/lox^) mice. Successful and selective deletion of SIK3 was confirmed in both models through PCR analysis of genomic DNA isolated from NPY and POMC expressing tissues, including the hypothalamus, cerebrum, olfactory bulb, and pituitary gland, while no recombination was detected in peripheral tissues that lack NPY or POMC expression, such as the liver (Supplementary Fig. S2A, B). Consistent with that, our results showed that SIK3 (green) co-localized with NPY and POMC-expressing neurons (tdTomato red) in the ARC of NPY^cre/+,Ai9/+^ (Supplementary Fig. S2C) and POMC^cre/+,Ai9+^ mice (Supplementary Fig. S2D). However, this co-localization was reduced in NPY^cre/+,Ai9/+^,SIK3^lox/lox^ (Supplementary Fig. S2C) and POMC^cre/+,Ai9/+^,SIK3^lox/lox^ mice (Supplementary Fig. S2D), confirming successful ablation of SIK3 in NPY and POMC neurons. Unlike global SIK3-deficient mice, which exhibit impaired development and dwarfism^43,59,60^, NPY^cre/+^,SIK3^lox/lox^ and POMC^cre/+^,SIK3^lox/lox^ mice are viable, with no overt phenotype showing no differences in litter size, litter intervals, gender ratio of pups, body length or body weight at 8-week of age (Supplementary Fig. S2E–J) compared to respective control littermates. Moreover, quantitative anatomical analysis revealed no differences in NPY and POMC neuronal counts between NPY^cre/Ai9^,SIK3^lox/lox^ and NPY^cre/Ai9^;SIK3^+/+^ mice as well as POMC^cre/Ai9^,SIK3^lox/lox^ and POMC^cre/Ai9^;SIK3^+/+^ mice (Supplementary Fig. S2K–N). These results indicate that SIK3 deficiency in NPY or POMC neurons does not impact upon reproductive fitness or postnatal development and growth towards adulthood.

**Fig. 2:**
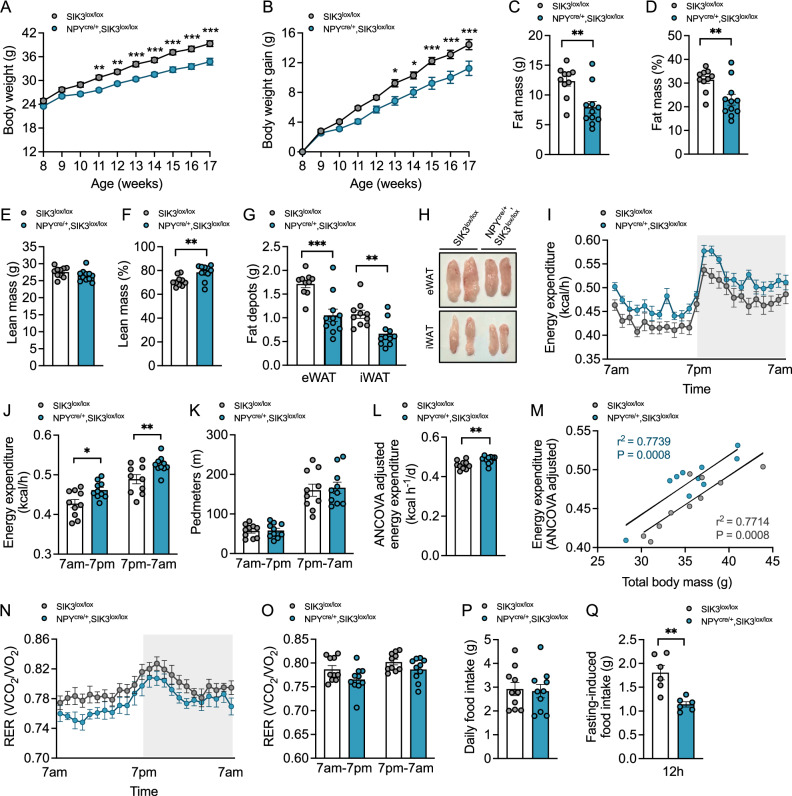
Selective deletion of SIK3 in NPY neurons attenuates diet-induced obesity. **A** Body weight and (**B**) body weight gain monitoring of HFD-fed SIK3^lox/lox^ and NPY^cre/+^,SIK3^lox/lox^ mice. *n* = 11 biologically independent samples per group. Data are mean ± s.e.m. *P < 0.05; **P < 0.01; ***P < 0.001, by two-way ANOVA with Sidak’s multiple comparisons. **C** Whole body fat mass measured by EchoMRI. **D** Fat mass % normalized to body weight. **E** Whole body lean mass measured by EchoMRI. **F** Lean mass % normalized to body weight. *n* = 10-11 biologically independent samples per group. Data are mean ± s.e.m. **P = 0.003 for (**C**); **P = 0.006 for (**D**); **P = 0.005 for (**F**), by two-tailed Student’s* t* test. Data are from at least two independent experiments. **G** Dissected weights of individual fat depots from epididymal white adipose tissue (eWAT) and inguinal white adipose tissue (iWAT). *n* = 10-11 biologically independent samples per group. Data are mean ± s.e.m. **P = 0.002; ***P = 0.0005, by two-tailed Student’s *t* test. **H** Representative images of eWAT and iWAT depots. n = 10-11 biologically independent samples per group. **I**, **J** Energy expenditure. **K** Pedmeters. *n* = 10 biologically independent samples per group. Data are mean ± s.e.m. *P = 0.011; **P = 0.007, by two-way ANOVA with Sidak’s multiple comparisons. **L**, **M** ANCOVA adjusted energy expenditure. *n* = 10 biologically independent samples per group. Data are mean ± s.e.m. **P = 0.009, by two-tailed Student’s *t* test; ***P = 0.0008, by simple linear regression. **N**, **O** Respiratory exchange ratio (RER). **P** Daily food intake. *n* = 10 biologically independent samples per group. Data are mean ± s.e.m. *P* > 0.05, by two-tailed Student’s *t* test (**Q**) Fasting-induced food intake. *n* = 6 biologically independent samples per group. Data are mean ± s.e.m. **P = 0.005, by two-tailed Student’s *t* test. Data are from at least two independent experiments. Source data are provided as a Source Data file.

To assess the impact of SIK3 deficiency in NPY neurons in the regulation of body weight and energy homeostasis, 8-week-old NPY^cre/+^,SIK3^lox/lox^ and SIK3^lox/lox^ were fed either a standard chow diet (SCD) or high fat diet (HFD) for 10 weeks. Under SCD feeding, body weight and body composition, including fat mass and lean mass were indistinguishable between SIK3^lox/lox^ and NPY^cre/+^, SIK3^lox/lox^ mice (Supplementary Fig. S3A–E). Apart from a modest increase in energy expenditure during the light cycle (Supplementary Fig. S3F, G), no differences in activity, food intake, or respiratory exchange ratio (RER) were observed between chow-fed SIK3^lox/lox^ and NPY^cre/+^,SIK3^lox/lox^ mice (Supplementary Fig. S3H–K). However, when challenged with chronic positive energy balance through 10 weeks of HFD feeding, NPY^cre/+^,SIK3^lox/lox^ mice exhibited significantly reduced body weight gain when compared to SIK3^lox/lox^ mice (Fig. 2A, B). EchoMRI analysis revealed the reduction in weight gain observed in NPY^cre/+^,SIK3^lox/lox^ mice was primarily attributed to a decrease in body fat mass (Fig. 2C, D). While absolute lean mass remained comparable (Fig. 2E), NPY^cre/+^,SIK3^lox/lox^ mice exhibited a significant increase in lean mass when normalized to body weight (Fig. 2F). Consistently, the weights of individually dissected white adipose tissue depots, including the inguinal (iWAT) and epididymal (eWAT), were significantly reduced in HFD-fed NPY^cre/+^,SIK3^lox/lox^ mice (Fig. 2G, H).

To test the effects of SIK3 deficiency in NPY neurons on energy homeostasis under HFD feeding, indirect calorimetry was performed on NPY^cre/+^,SIK3^lox/lox^ mice. This revealed a significant increase in energy expenditure, both in absolute terms (Fig. 2I, J) and when normalized to lean mass (Supplementary Fig. S4A), during both the light (7am-7pm) and dark (7pm-7am) cycles compared to SIK3^lox/lox^ mice. By contrast, ambulatory activity was indistinguishable between NPY^cre/+^,SIK3^lox/lox^ and SIK3^lox/lox^ mice (Fig. 2K). To validate whether the increased energy expenditure observed is independent of body mass, a regression analysis was conducted. ANCOVA-adjusted daily energy expenditure remained significantly higher in NPY^cre/+^,SIK3^lox/lox^ compared to SIK3^lox/lox^ mice (Fig. 2L, M). In addition, NPY^cre/+^,SIK3^lox/lox^ mice also exhibited a modest, albeit non-significant, decrease in respiratory exchange ratio (RER) during the light cycle (Fig. 2N, O), suggesting a trend in substrate utilization toward fat over carbohydrates as the primary fuel source. While ad libitium food intake was comparable between genotypes (Fig. 2P and Supplementary Fig. S4B), fasting-induced food intake was significantly lower in HFD-fed NPY^cre/+^,SIK3^lox/lox^ mice (Fig. 2Q and Supplementary Fig. S4C). Together, these results suggest that both increased energy expenditure and reduced energy intake contribute to the reduced body weight gain and adiposity in HFD-fed NPY^cre/+^,SIK3^lox/lox^ mice.

### SIK3 deficiency in NPY neurons enhances BAT thermogenesis and promotes WAT browning

**Fig. 3:**
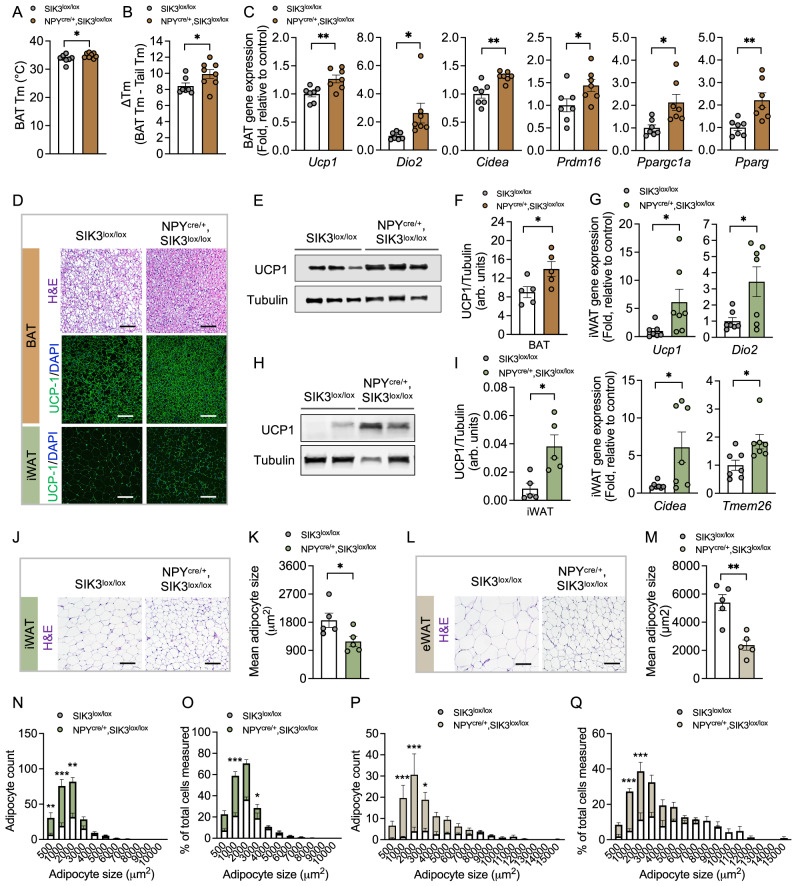
SIK3 deficiency in NPY neurons enhances BAT thermogenesis and WAT browning. **A** Brown adipose tissue (BAT) dermal thermography on shaved HFD-fed SIK3^lox/lox^ and NPY^cre/+^,SIK3^lox/lox^ mice. *n* = 7-8 biologically independent samples per group. Data are mean ± s.e.m. *P = 0.044, by one-tailed Student’s *t* test. **B** Tail corrected BAT dermal thermography. *n* = 7-8 biologically independent samples per group. Data are mean ± s.e.m. *P = 0.048, by two-tailed Student’s *t* test. Values from the FLIR infrared camera. **C** mRNA expression levels of thermogenic and mitochondrial function/biogenesis markers in intrascapular BAT of HFD-fed SIK3^lox/lox^ and NPY^cre/+^,SIK3^lox/lox^ mice. *n *= 7 biologically independent samples per group. Data are mean ± s.e.m. *P < 0.05; **P < 0.01, by two-tailed Student’s *t* test. **D** Representative H&E and UCP-1 immunohistochemistry staining of BAT and iWAT. Scale bar, 100 µm. *n* = 4 biologically independent samples per group. **E**, **F** Immunoblot densitometry analyses of UCP-1 protein levels in BAT of HFD-fed SIK3^lox/lox^ and NPY^cre/+^,SIK3^lox/lox^ mice. *n* = 5 biologically independent samples per group. Data are mean ± s.e.m. *P = 0.037, by two-tailed Student’s *t* test. **G** Thermogenic/browning markers in iWAT. *n* = 7 biologically independent samples per group. Data are mean ± s.e.m. *P < 0.05, by two-tailed Student’s *t* test. **H**, **I** Immunoblot densitometry analyses of UCP-1 protein levels in iWAT. *n* = 5 biologically independent samples per group. Data are mean ± s.e.m. *P = 0.011, by two-tailed Student’s *t* test. **J** Representative H&E staining of iWAT. Scale bar, 100 µm. **K** Mean adipocyte size of iWAT. **L** Representative H&E staining of eWAT. Scale bar, 100 µm. **M** Mean adipocyte size of eWAT. *n* = 5 biologically independent samples per group. Data are mean ± s.e.m. *P = 0.040; **P = 0.002, by two-tailed Student’s *t* test. **N** Adipocyte count of iWAT. **O** % of total iWAT cells measured. **P** Adipocyte count of eWAT. **Q** % of total eWAT cells measured. *n* = 5 biologically independent samples per group. Data are mean ± s.e.m. *P < 0.05; **P < 0.01; ***P < 0.001, by two-way ANOVA with Sidak’s multiple comparisons. Source data are provided as a Source Data file.

One of the central mechanisms of energy expenditure is adaptive thermogenesis, where the body adjusts its temperature, particularly in the brown adipose tissue (BAT) region, to regulate energy balance based on the current energy status^61^. Thus, we next examined whether increased BAT thermogenesis could account for the rise in energy expenditure in NPY^cre/+^,SIK3^lox/lox^ mice using non-invasive high-sensitivity infrared imaging (Supplementary Fig. S4D). Dermal thermography of the interscapular BAT (BAT Tm) (Fig. 3A, B), when normalized to tail temperature, was significantly increased in NPY^cre/+^,SIK3^lox/lox^ mice, indicating enhanced BAT thermogenesis. By contrast, tail (Tail Tm) and lumbar (Lumbar Tm) dermal thermography were comparable between the two genotypes, indicating normal core body temperature and heat dissipation (Supplementary Fig. S4E, F). Consistent with increased thermogenesis, *Ucp-1* mRNA levels in the BAT were significantly elevated in NPY^cre/+^,SIK3^lox/lox^ mice compared to SIK3^lox/lox^ mice (Fig. 3C). Histology and western blot analysis further revealed that the elevated *Ucp-1* mRNA in the BAT was accompanied by an increase in UCP-1 protein levels (Fig. 3D–F). In addition, other critical thermogenic markers, including type 2 iodothyronine deiodinase (*Dio2*), an essential component of the thyroid-sympathetic circuitry in adaptive thermogenesis^62^, and cell death-inducing DNA fragmentation factor alpha-like effector A (*Cidea*), which promotes lipid droplet fusion to form multilocular adipocytes capable of rapidly consuming lipids to enhance thermogenesis^63^, were also significantly elevated in the BAT of NPY^cre/+^,SIK3^lox/lox^ mice (Fig. 3C). Collectively, these results suggest that SIK3 deficiency in NPY neurons increases thermogenesis by upregulating BAT activity.

In addition to BAT thermogenesis, the browning of white adipose tissues has been linked to increased energy expenditure and occurs predominantly in subcutaneous fat depots, such as iWAT^64^. To assess whether SIK3 deficiency enhances WAT browning, we examined the mRNA expression of several key brown fat-related genes in iWAT. We found the mRNA expression of thermogenic genes, including *Ucp-1, Dio2*, and *Cidea*, were significantly upregulated in iWAT of NPY^cre/+^,SIK3^lox/lox^ mice (Fig. 3G). Consistent with the mRNA data, UCP-1 protein levels were also significantly increased in the iWAT of NPY^cre/+^,SIK3^lox/lox^ mice (Fig. 3D, H and I). While the expression of mitochondrial biogenesis and function markers such as *Pgc1-α*, *Ppar-γ* and *Prdm16* in iWAT remained comparable between genotypes (Supplementary Fig. S4G–I), the gene expression of signature beiging/brite marker, *Tmem26*, was significantly upregulated in NPY^cre/+^,SIK3^lox/lox^ mice (Fig. 3G). To further assess whether increased BAT thermogenesis and WAT browning led to alterations in adipocyte morphology, we conducted histological analysis on iWAT of SIK3^lox/lox^ and NPY^cre/+^,SIK3^lox/lox^ mice. H&E staining revealed a significantly smaller mean adipocyte size in both iWAT (Fig. 3J, K) and eWAT (Fig. 3L, M) of NPY^cre/+^,SIK3^lox/lox^ mice compared to SIK3^lox/lox^. In terms of adipocyte distribution, our results show that NPY^cre/+^,SIK3^lox/lox^ mice have a significantly higher number and percentage of smaller adipocytes in both iWAT (Fig. 3N, O) and eWAT tissues (Fig. 3P, Q). Taken together, these results demonstrate that deletion of SIK3 in NPY neurons promotes both BAT thermogenesis and WAT browning, together contributing to increased energy expenditure and reduced adiposity.

### Reduced orexigenic neuropeptides and enhanced SNS activity in NPY neuron-specific SIK3 deficient mice

**Fig. 4:**
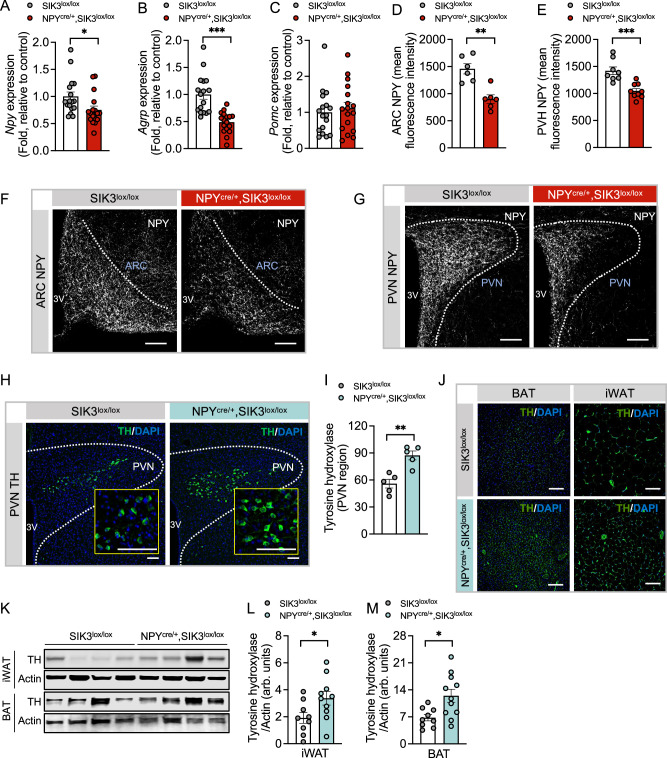
Reduced orexigenic neuropeptides and enhanced SNS activity in NPY neuron-specific SIK3 deficient mice. **A** *Npy*, (**B**) *Agrp*, and (**C**) *Pomc* neuropeptide expression in the whole hypothalamus of HFD-fed SIK3^lox/lox^ and NPY^cre/+^,SIK3^lox/lox^ mice. *n* = 17 biologically independent samples per group. Data are mean ± s.e.m. *P = 0.029 for (**A**); ***P < 0.001 for (**B**), by two-tailed Student’s *t* test. Data are from at least two independent experiments. **D**, **F** ARC NPY innervation. *n* = 6 biologically independent samples per group. Data are mean ± s.e.m. **P = 0.0011, by two-tailed Student’s *t* test. **E**, **G** PVN NPY innervation. Quantified as mean fluorescence intensity. *n* = 8-9 biologically independent samples per group. Data are mean ± s.e.m. ***P = 0.0004, by two-tailed Student’s *t* test. **F** Representative image of ARC NPY immunostaining. **G** Representative image of PVN NPY immunostaining. Scale bar, 100 µm. **H**, **I** Immunostaining of tyrosine hydroxylase (TH) in the PVN. Scale bar, 100 µm. *n* = 5 biologically independent samples per group. Data are mean ± s.e.m. **P = 0.0019, by two-tailed Student’s *t* test. **J** Immunostaining of TH in BAT and iWAT. Scale bar, 100 µm. *n* = 5 biologically independent samples per group. **K**–**M** Immunoblot and densitometry analyses of TH in BAT and iWAT of HFD-fed SIK3^lox/lox^ and NPY^cre/+^,SIK3^lox/lox^ mice. *n* = 9–11 biologically independent samples per group. Data are mean ± s.e.m. *P = 0.039 for (**L**); *P = 0.011 for (**M**), by two-tailed Student’s *t* test. Source data are provided as a Source Data file.

To investigate the underlying mechanism causing the altered energy metabolism in our HFD-fed NPY^cre/+^,SIK3^lox/lox^ mice, we next examined the expression of *Npy*, *Agrp* and *Pomc* in the hypothalamus of NPY^cre/+^,SIK3^lox/lox^ and SIK3^lox/lox^ mice. Lack of SIK3 in NPY neurons led to a significant downregulation of orexigenic *Npy* and *Agrp* expression in NPY^cre/+^,SIK3^lox/lox^ mice, whereas hypothalamic *Pomc* mRNA expression was comparable between the two genotypes (Fig. 4A–C). Previous studies have shown hypothalamic NPY reduces BAT thermogenesis and WAT browning through the sympathetic nervous system. Specifically, ARC-derived NPY inhibits the activity of Tyrosine Hydroxylase (TH) neurons in the PVN, which subsequently dampens sympathetic nervous system (SNS)-mediated BAT thermogenesis^65^. Consistent with this, we observed a significant reduction in NPY innervating the ARC (Fig. 4D, F) and the PVN (Fig. 4E, G) of HFD-fed NPY^cre/+^,SIK3^lox/lox^ mice when compared to SIK3^lox/lox^ mice. To determine whether reduced NPY innervation in the ARC and PVN leads to increased SNS activity in NPY^cre/+^,SIK3^lox/lox^ mice, we assessed TH expression in the PVN. Our immunofluorescence analysis revealed that TH expression was significantly elevated in the PVN of the HFD-fed NPY^cre/+^,SIK3^lox/lox^ compared to SIK3^lox/lox^ mice (Fig. 4H, I). Consistent with the enhancement of SNS activity towards peripheral tissues, NPY^cre/+^,SIK3^lox/lox^ mice also exhibited significantly higher TH expression in both iWAT and BAT (Fig. 4J–M). To further support that the observed phenotype in NPY^cre/+^,SIK3^lox/lox^ mice was attributable to altered NPY activity, we selectively overexpressed NPY in HFD-fed NPY^cre/+^,SIK3^lox/lox^ mice via bilateral administration of a Cre-dependent AAV construct (AAV-CAG > FLEX-NPY-P2A-GFP) (Supplementary Fig. S5A–D), allowing targeted restoration of NPY expression within the arcuate nucleus (Supplementary Fig. S5E). Notably, restoring NPY expression abolished the reduction in body weight and adiposity observed in NPY^cre/+^,SIK3^lox/lox^ (Supplementary Fig. S5F–H) by decreasing energy expenditure compared to control mice (Supplementary Fig. S5I–L). These findings indicate that resistance to diet-induced weight gain is mediated, at least in part, by dampened NPY activity, confirming a cell-autonomous role for SIK3 in regulating NPY neuron function. Coupled with a reduction in energy intake, these results causally link SIK3 deficiency in NPY neurons to enhanced SNS-mediated BAT thermogenesis and iWAT browning, culminating in increased energy expenditure and reductions in adiposity and body weight.

### Improved glycaemic control in NPY neuron-specific SIK3 deficient mice

**Fig. 5:**
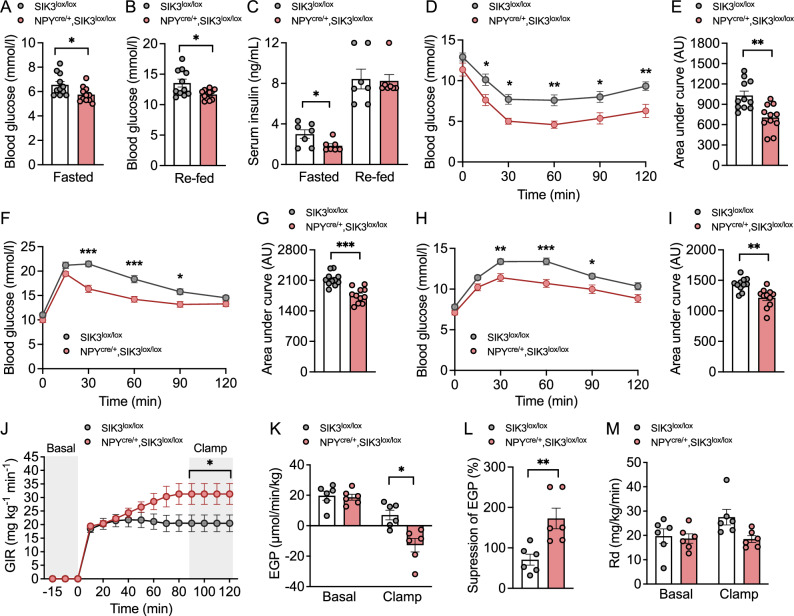
Improved glycemic control in NPY neuron-specific SIK3 deficient mice. **A** 16 h fasted blood glucose. **B** 30 mins re-fed blood glucose. *n* = 11 biologically independent samples per group. Data are mean ± s.e.m. *P = 0.025 for (A); *P = 0.013 for (**B**), by two-tailed Student’s *t* test. **C** 16 h fasted and 30 mins re-fed serum insulin levels. *n* = 7 biologically independent samples per group. Data are mean ± s.e.m. *P = 0.029, by two-tailed Student’s *t* test. **D**, **E** Intraperitoneal insulin tolerance test on 4 h fasted mice [0.75 IU/kg body weight]. **F**, **G** Intraperitoneal glucose tolerance test on 6 h fasted mice [1 g/kg body weight]. **H**, **I** Intraperitoneal pyruvate tolerance test on 16 h fasted mice [1 g/kg body weight]. Blood glucose levels during each tolerance test were monitored at 0, 15, 30, 60, 90, and 120 min. Results are expressed over the time course as the area under the curve. *n* = 11 biologically independent samples per group. Data are mean ± s.e.m. *P < 0.05; **P < 0.01; ***P < 0.001, by two-tailed Student’s *t* test for (**E**, **G**,** I**) or two-way ANOVA with Sidak’s multiple comparisons for (**D**,** F**, **H**). Data are from at least two independent experiments. Hyperinsulinaemic-euglycaemic clamps were performed on HFD-fed SIK3^lox/lox^ and NPY^cre/+^,SIK3^lox/lox^ mice. **J** Glucose infusion rate (GIR). **K** Endogenous glucose production (EGP). **L** % of EGP Suppression. **M** Glucose disposal rate (Rd). *n* = 6 biologically independent samples per group. Data are mean ± s.e.m. *P < 0.05 for (**J**), by two-way ANOVA with Sidak’s multiple comparisons; *P = 0.024 for (**K**); **P = 0.005 for (**L**), by two-tailed Student’s *t* test. Source data are provided as a Source Data file.

Given that reduced adiposity is associated with improved glycaemic control, we next measured several glucose homeostasis parameters in SIK3^lox/lox^ and NPY^cre/+^,SIK3^lox/lox^ mice. Both fasted and re-fed blood glucose levels were significantly lower in NPY^cre/+^,SIK3^lox/lox^ mice (Fig. 5A, B). The reduction in fasting blood glucose levels was accompanied by a significant decrease in fasted insulin levels, indicative of improved insulin sensitivity (Fig. 5C). No significant difference was observed in re-fed insulin levels between NPY^cre/+^,SIK3^lox/lox^ and SIK3^lox/lox^ mice, suggesting that pancreatic β-cell insulin secretion was not altered by SIK3 deficiency in NPY neurons (Fig. 5C). To assess insulin sensitivity and glucose tolerance directly, we performed insulin and glucose tolerance tests. NPY^cre/+^,SIK3^lox/lox^ mice exhibited significantly increased insulin responsiveness compared to SIK3^lox/lox^ mice, with blood glucose levels reaching statistical significance at the 30-, 60-, and 120-minute time points post-insulin injection (Fig. 5D, E). In addition, whole-body glucose clearance following administration of a bolus of glucose was also significantly improved in NPY^cre/+^,SIK3^lox/lox^ mice (Fig. 5F, G). Although Cre-mediated recombination occurred in pancreatic islets of NPY^cre/+^,SIK3^lox/lox^ mice, glucose-stimulated insulin secretion remained unchanged between NPY^cre/+^,SIK3^lox/lox^ and SIK3^lox/lox^ mice, indicating unaltered β-cell function (Supplementary Fig. S6A B). The significantly lower blood glucose levels in response to pyruvate administration suggests that NPY^cre/+^,SIK3^lox/lox^ mice exhibited reduced hepatic glucose production (Fig. 5H, I). To comprehensively assess the contribution of individual tissues in the regulation of glucose homeostasis, a hyperinsulinemic-euglycemic clamp experiment was performed on HFD-fed NPY^cre/+^,SIK3^lox/lox^ and SIK3^lox/lox^ mice (Supplementary Fig. S6C). Significantly increased glucose infusion rates (GIR) were observed in NPY^cre/+^,SIK3^lox/lox^ mice, indicating enhanced whole-body insulin sensitivity (Fig. 5J). This improvement in insulin responsiveness was accompanied by a reduction in endogenous hepatic glucose production (Fig. 5K, L), without affecting glucose disposal rate (Fig. 5M). While 2-DG glucose uptake data from the clamps showed no significant difference in glucose uptake in muscle and heart (Supplementary Fig. S6D), there was a significant increase in 2-DG glucose uptake in iWAT (Supplementary Fig. S6E), which is consistent with the enhanced iWAT browning phenotype in NPY^cre/+^, SIK3^lox/lox^ mice. Collectively, these results suggest that the enhancement in whole body glucose homeostasis in mice deficient in SIK3 in NPY neurons may be attributable to improved insulin sensitivity and suppression in hepatic glucose production.

To determine sex specificity, we performed parallel metabolic phenotyping in female NPY^cre/+^,SIK3^lox/lox^ and SIK3^lox/lox^ mice under HFD. Female NPY^cre/+^,SIK3^lox/lox^ mirrored the male phenotype, exhibiting reduced weight gain and adiposity (Supplementary Fig. S6F–I), preserved lean mass (Supplementary Fig. S6J, K), driven by increased energy expenditure, while food intake, activity and RER remained unchanged (Supplementary Fig. S6L–O). In addition female NPY^cre/+^,SIK3^lox/lox^ mice also exhibited significantly improved glycemic control (Supplementary Fig. S6P, Q) and insulin responsiveness (Supplementary Fig. S6R, S). These findings demonstrate that NPY-neuron specific SIK3 deletion confers resistance to diet-induced obesity and improves glucose homeostasis in a sex-independent manner.

### CRTC1 and HDAC5 in the hypothalamus are downstream targets of SIK3

**Fig. 6:**
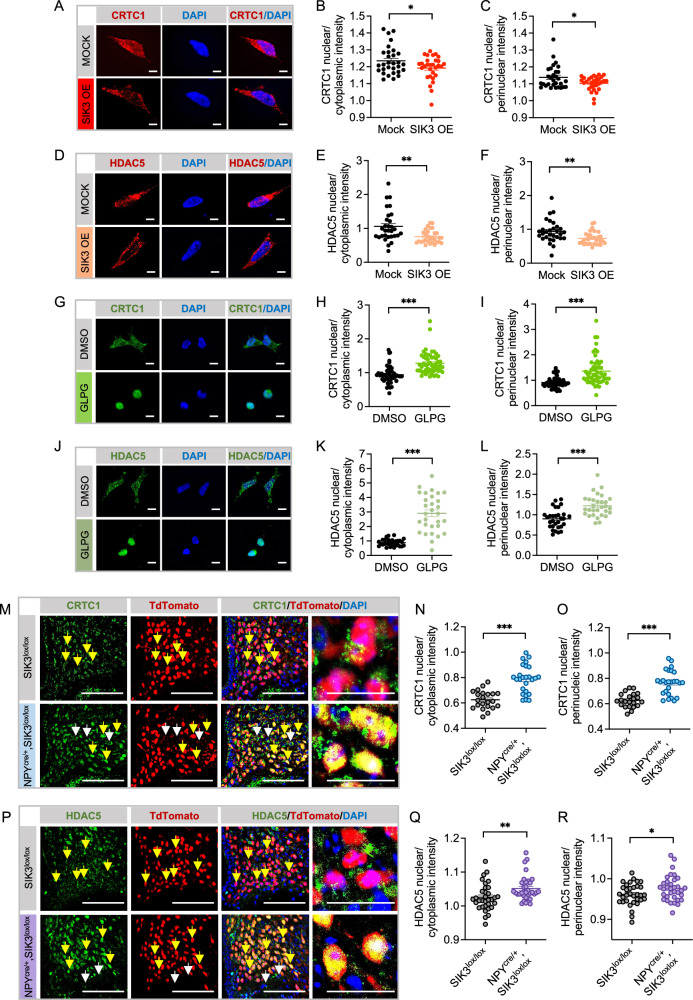
CRTC1 and HDAC5 in the hypothalamus are downstream targets of SIK3. **A** CRTC1 immunostaining in wild-type/mock and SIK3 overexpressed (SIK3 OE) SK-N-SH-SY5Y neuroblastoma cells. Results analyzed as: (**B**) CRTC1 Nuclear/Cytoplasmic ratio and (**C**) CRTC1 Nuclear/Perinuclear ratio in Mock and SIK3 OE cells. Each dot corresponds to the nuclear/perinuclear-to-cytoplasmic CRTC1 intensity ratio in discrete areas of coverslips. Scale bar, 5 µm. *n* = 30 per group. **D** HDAC5 immunostaining in Mock and SIK3 OE SK-N-SH-SY5Y neuroblastoma cells. Results analyzed as: (**E**) HDAC5 Nuclear/Cytoplasmic ratio and (**F**) HDAC5 Nuclear/Perinuclear ratio in Mock and SIK3 OE cells. Scale bar, 5 µm. *n* = 30 per group. Data are mean ± s.e.m. *P = 0.034 for (**B**); *P = 0.016 for (**C**); **P = 0.0014 for (**E**); **P = 0.0048 for (**F**), by two-tailed Student’s *t* test. *n* corresponds to the nuclear/perinuclear-to-cytoplasmic CRTC1/HDAC5 intensity ratio in discrete areas of coverslips. Data are derived from three independent experiments. **G** CRTC1 immunostaining in DMSO and GLPG3970 treated SK-N-SH-SY5Y neuroblastoma cells. Results analyzed as: (**H**) CRTC1 Nuclear/Cytoplasmic ratio and (**I**) CRTC1 Nuclear/Perinuclear ratio in DMSO and GLPG3970 treated cells. Scale bar, 5 µm. *n* = 48-53 per group (**J**) HDAC5 immunostaining in DMSO and GLPG3970 treated SK-N-SH-SY5Y neuroblastoma cells. Results analyzed as: (**K**) HDAC5 Nuclear/Cytoplasmic ratio and (**L**) HDAC5 Nuclear/Perinuclear ratio in DMSO and GLPG3970 treated cells. Scale bar, 5 µm. n = 30 per group. Data are mean ± s.e.m. ***P < 0.001 for (**H**,** I**, **K**, **L**), by two-tailed Student’s *t* test. *n* corresponds to the nuclear/perinuclear-to-cytoplasmic CRTC1/HDAC5 intensity ratio in discrete areas of coverslips. Data are derived from three independent experiments. **M** Immunostaining of CRTC1 in HFD-fed SIK3^lox/lox^ and NPY^cre/+^;SIK3^lox/lox^ mice. (Yellow arrow represents CRTC1 staining localized on NPY neurons. The white arrow represents CRTC1 staining non-localized to NPY neurons). **N** CRTC1 Nuclear/Cytoplasmic ratio and (**O**) CRTC1 Nuclear/Perinuclear ratio. Scale bar, 100 µm and 25 µm (zoomed-in images). *n* = 22-26 per group. Data are mean ± s.e.m. ***P < 0.001, by two-tailed Student’s *t* test. *n* corresponds to the nuclear/perinuclear-to-cytoplasmic CRTC1 intensity ratio in one brain section. Data were derived from 4-5 biologically independent replicates. **P** HDAC5 immunostaining in HFD-fed SIK3^lox/lox^ and NPY^cre/+^,SIK3^lox/lox^ mice. (Yellow arrow represents HDAC5 staining localized on NPY neurons. The white arrow represents HDAC5 staining non-localized to NPY neurons). **Q** HDAC5 Nuclear/Cytoplasmic ratio. **R** HDAC5 Nuclear/Perinuclear ratio. Scale bar, 100 µm and 25 µm (zoomed-in images). *n* = 33-34 per group. Data are mean ± s.e.m. **P = 0.007 for (**Q**); *P = 0.040 for (**R**), by two-tailed Student’s *t* test. *n* corresponds to the nuclear/perinuclear-to-cytoplasmic HDAC5 intensity ratio in one brain section. Data were derived from 4-5 biologically independent replicates. Source data are provided as a Source Data file.

Two well established downstream targets of the SIK family are the CRTCs (CRTC1, CRTC2, and CRTC3) and class IIa HDACs (HDAC4, 5, 7, and 9)^26,36,66^. Among these isoforms, CRTC1 and HDAC5 are highly expressed in the brain, particularly in the hypothalamus^67–72^. Both CRTC1 and HDAC5 have been implicated in the regulation of food intake and energy balance, and a deficiency in either CRTC1 or HDAC5 has been associated with the development of hyperphagia and obesity^70–72^. This led us to hypothesize that CRTC1 and HDAC5 may be a downstream target of SIK3 in the context of the central regulation of body weight and energy homeostasis. We examined SIK3’s capacity to regulate CRTC1 and HDAC5 in SK-N-SH-SY5Y neuroblastoma cells in vitro. Overexpression of SIK3 in SK-N-SH-SY5Y cells (Supplementary Fig. S7A, B) resulted in cytoplasmic retention or a reduction in nuclear levels of CRTC1 (Fig. 6A–C) and HDAC5 (Fig. 6D–F). To complement these overexpression studies, we pharmacologically inhibited SIK3 using the selective SIK2/3 inhibitor, GLPG3970 in vitro in SK-N-SH-SY5Y neuroblastoma cells. By contrast, SIK3 inhibition by GLPG3970 resulted in enhanced nuclear accumulation of CRTC1 (Fig. 6G–I) and HDAC5 (Fig. 6J–L) in SK-N-SH-SY5Y cells. Consistently, SIK3 inhibition by GLPG3970 also resulted in enhanced nuclear accumulation of CRTC1 (Supplementary Fig. S7C–E) and HDAC5 (Supplementary Fig. S7F–H) in the MBH in vivo. This finding is consistent with the notion that SIK3 inhibition promotes the dephosphorylation of CRTC1 and HDAC5, enabling these two downstream substrates to enter the nucleus and exert their effect on regulating transcription factors. Consistent with our in vitro findings, we observed that CRTC1 (Fig. 6M–O) and HDAC5 (Fig. 6P–R) nuclear-to-cytoplasmic intensity was significantly elevated in the ARC of NPY^cre/+^,SIK3^lox/lox^ mice. Further immunostaining analysis revealed that CRTC1 and HDAC5 nuclear translocation predominantly occurred in NPY-expressing neurons in the ARC of NPY^cre/+,Ai9/+^,SIK3^lox/lox^ mice, where SIK3 was specifically ablated (Fig. 6M, P). In contrast, nuclear accumulation of CRTC1 and HDAC5 was markedly reduced in non-NPY-expressing neurons (Fig. 6M, P). Collectively, these results suggest that SIK3 phosphorylates and inhibits CRTC1 and HDAC5 in hypothalamic neurons and the protective effects against diet-induced obesity observed by SIK3 deficiency in NPY neurons were, at least in part, mediated through the CRTC1 and HDAC5 signaling cascade.

## Enhanced central leptin and insulin signaling in NPY neuron-specific SIK3 deficient mice

**Fig. 7:**
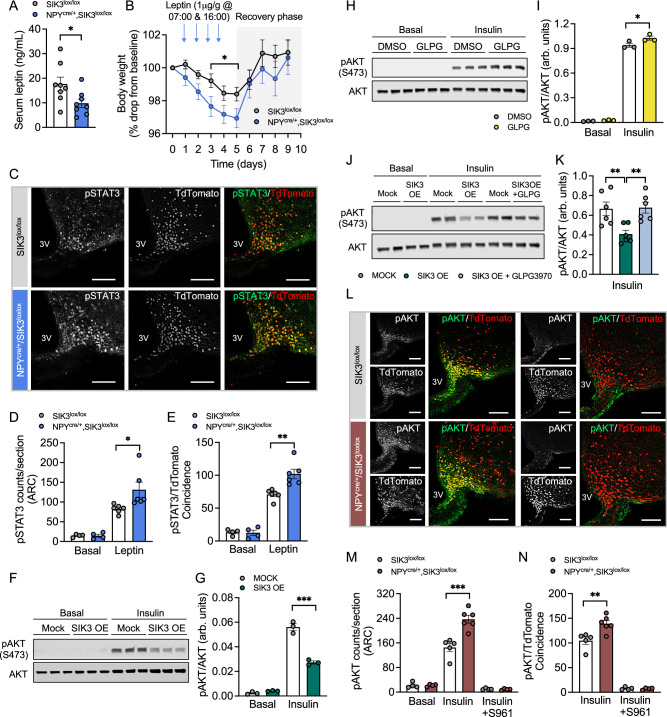
Enhanced central leptin and insulin signaling in NPY neuron-specific SIK3 deficient mice. **A** Serum leptin levels from HFD-fed SIK3^lox/lox^ and NPY^cre/+^,SIK3^lox/lox^ mice. *n* = 8 biologically independent samples per group. Data are mean ± s.e.m. *P = 0.034, by two-tailed Student’s *t* test. **B** HFD-fed SIK3^lox/lox^ and NPY^cre/+^,SIK3^lox/lox^ mice were intraperitoneally injected with 1 µg/g leptin twice daily for 4 days. % Drop of body weight from baseline were monitored. Area under the curve was calculated. *n* = 5-6 biologically independent samples per group. Data are mean ± s.e.m. *P < 0.05, by one-tailed Student’s *t* test. **C**–**E** SIK3^lox/lox^ and NPY^cre/+^,SIK3^lox/lox^ mice were fasted overnight and intraperitoneally injected with saline or 1 µg/g leptin for 45 min. Brains were extracted for ARC p-STAT3 (Tyr 705) immunostaining and p-STAT3 positive NPY neurons quantified across the rostral-caudal extent of the hypothalamus. Scale bar, 100 µm. *n* = 4-6 biologically independent samples per group. Data are mean ± s.e.m. *P = 0.035 for (**D**); **P = 0.0028 for (**E**), by two-tailed Student’s *t* test. **F**, **G** Immunoblot and densitometry analysis of p-AKT (Ser 473), total AKT in wild-type/Mock and SIK3 overexpressed (SIK3 OE) SK-N-SH-SY5Y cells stimulated with or without insulin for 15 min. Data are from three independent experiments. Data are mean ± s.e.m. ***P = < 0.001, by two-tailed Student’s *t* test. **H**, **I** Immunoblot and densitometry analysis of p-AKT (Ser 473), total AKT in DMSO and GLPG3970 treated SK-N-SH-SY5Y cells stimulated with or without insulin for 15 min. Data are from three independent experiments. Data are mean ± s.e.m. *P = 0.035, by two-tailed Student’s *t* test. **J**, **K** Immunoblot and densitometry analysis of p-AKT (Ser 473), total AKT in wild-type/Mock, SIK3 OE and SIK3 OE + GLPG3970 treated SK-N-SH-SY5Y neuroblastoma cells stimulated with or without insulin for 15 min. *n* = 6 per group from three independent experiments. Data are mean ± s.e.m. **P = 0.008 (Mock vs SIK3 OE); **P = 0.002 (SIK3 OE vs SIK3 OE + GLPG), by two-tailed Student’s *t* test. (**L**) (M) (N) SIK3^lox/lox^ and NPY^cre/+^,SIK3^lox/lox^ mice were fasted overnight and intraperitoneally injected with saline or insulin (2.5 IU/kg body weight) for 15 min, injected with or without S961 IR antagonist for 1 hour. Brains were extracted for ARC p-AKT (Ser 473) immunohistochemistry and p-AKT positive NPY neurons quantified across the rostral-caudal extent of the hypothalamus. Scale bar, 100 µm.* n* = 4-6 biologically independent samples per group. Data are mean ± s.e.m. ***P < 0.001 for (**M**); **P = 0.006 for (**N**), by two-tailed Student’s *t* test. Source data are provided as a Source Data file.

Increased central leptin signaling and sensitivity is associated with reduced appetite and increased energy expenditure, as well as enhanced sympathetic activity to adipose tissue, leading to lower adiposity and circulating leptin levels^53,73–75^. Previous studies have demonstrated that both genetic ablation and pharmacological inhibition of CRTC1 and HDAC5 in the hypothalamus lead to hyperphagia and exacerbated diet-induced obesity^71,72^. These effects were primarily attributed to impaired central leptin signaling^71,72^. Consistent with increased SIK3 expression in obesity, we found that phosphorylation of CRTC1 (Ser151) was increased in the ARC of leptin receptor-deficient *db/db* mice compared to their lean *db/+* counterparts (Supplementary Fig. S7I, J). Having identified hypothalamic CRTC1 and HDAC5 as downstream targets of SIK3, we next examined the effects of SIK3 deficiency on leptin signaling and sensitivity. We found serum leptin levels were significantly decreased in HFD-fed NPY^cre/+^,SIK3^lox/lox^ mice (Fig. 7A), indicating enhanced leptin sensitivity. To directly examine leptin sensitivity, we administered leptin to NPY^cre/+^,SIK3^lox/lox^ and SIK3^lox/lox^ mice in the morning (at 07:00) and the late afternoon (at 16:00) for 4 consecutive days and recorded daily body weights. The effect of leptin was enhanced in NPY^cre/+^,SIK3^lox/lox^ mice as evidenced by a significant reduction in body weight compared to SIK3^lox/lox^ mice (Fig. 7B). Furthermore, the heightened leptin sensitivity coincided with enhanced hypothalamic leptin signaling as demonstrated by significantly elevated leptin-induced STAT3 Y705 phosphorylation in the ARC of NPY^cre/+^,SIK3^lox/lox^ mice compared to controls (Fig. 7C–E). These results demonstrate that SIK3 deficiency in NPY neurons enhances central leptin signaling, serving as a critical mechanism for the protective effects against diet-induced weight gain.

In addition to leptin, insulin acts on hypothalamic neurons to suppress hepatic glucose production, increase brown adipose tissue activity and thermogenesis, and promote the browning of WAT^76–80^. Moreover, considering SIK2 is a negative regulator of insulin signaling in adipocytes^37^, we hypothesized this could also be the underlying mechanism for the enhanced energy expenditure and improved glucose homeostasis observed in NPY^cre/+^,SIK3^lox/lox^ mice. Accordingly, we assessed whether increased expression of SIK3 suppresses insulin signaling in control and SIK3-overexpressing SK-N-SH-SY5Y neuroblastoma cells. We found that overexpression of SIK3 significantly reduced insulin signaling, as indicated by the markedly decreased levels of AKT Ser473 phosphorylation, as assessed by immunoblotting (Fig. 7F, G). By contrast, pharmacological inhibition of SIK3 in SK-N-SH-SY5Y cells using the SIK2/3 inhibitor GLPG3970 resulted in a significant increase in AKT Ser473 phosphorylation (Fig. 7H, I). Importantly, the suppression of insulin-induced AKT phosphorylation due to SIK3 overexpression was reversed when the cells were treated with GLPG3970. (Fig. 7J, K). Consistently, insulin-induced AKT Ser473 phosphorylation was significantly increased in the ARC of HFD-fed NPY^cre/+^,SIK3^lox/lox^ mice compared to SIK3^lox/lox^ mice, while at baseline AKT activation levels were comparable between the two groups (Fig. 7L–N). Conversely, the enhancement of insulin-induced AKT phosphorylation in NPY^cre/+^,SIK3^lox/lox^ mice was abolished when the mice were treated with S961, a biosynthetic insulin receptor antagonist (Fig. 7L–N). These results indicate that hypothalamic SIK3 signaling is associated with the negative regulation of central leptin and insulin signaling. This regulation occurs, at least in part, through the phosphorylation and inhibition of key co-regulators, including CRTC1 and HDAC5. The inhibition of SIK3, which enhances central leptin and insulin signaling, may explain how SIK3 deficiency in NPY neurons protects against diet-induced weight gain and improves glycaemic control.

## Ablation of SIK3 in POMC neurons does not affect energy homeostasis and glucose metabolism

To assess the significance of SIK3 signaling in anorexigenic POMC neurons in regulating body weight and energy balance, we performed similar metabolic characterization in SIK3^lox/lox^ and POMC^cre/+^,SIK3^lox/lox^ mice. While SIK3 deficiency in orexigenic NPY neurons elicited protective effects against diet-induced weight gain, SIK3^lox/lox^ and POMC^cre/+^,SIK3^lox/lox^ mice exhibited comparable body weight and adiposity in response to high-fat feeding (Supplementary Fig. S8A–C). Indistinguishable body weight and body composition (lean and fat masses) between the two groups were confirmed by equivalent weights during weekly body weight monitoring and EchoMRI assessments (Supplementary Fig. S8B–E) as well as comparable weights of epididymal and inguinal fat depots (Supplementary Fig. S8F, G). Consistent with unaltered adiposity, indirect calorimetry analysis revealed no significant differences in energy expenditure (Supplementary Fig. S8H, I), ambulatory activity (Supplementary Fig. S8J), respiratory exchange ratio (Supplementary Fig. S8K, L), or food intake (Supplementary Fig. S8M, N) between SIK3^lox/lox^ and POMC^cre/+^,SIK3^lox/lox^ mice. Moreover, fasted blood glucose levels, whole-body glucose clearance, insulin sensitivity, and hepatic glucose production were also comparable between the two groups (Supplementary Fig. S8O–R). Taken together, these findings suggest that the deletion of SIK3 in POMC neurons does not play a regulatory role in whole-body energy or glucose homeostasis within the hypothalamus.

## Pharmacological inhibition of SIK3 promotes weight loss and improves metabolic health outcomes

**Fig. 8:**
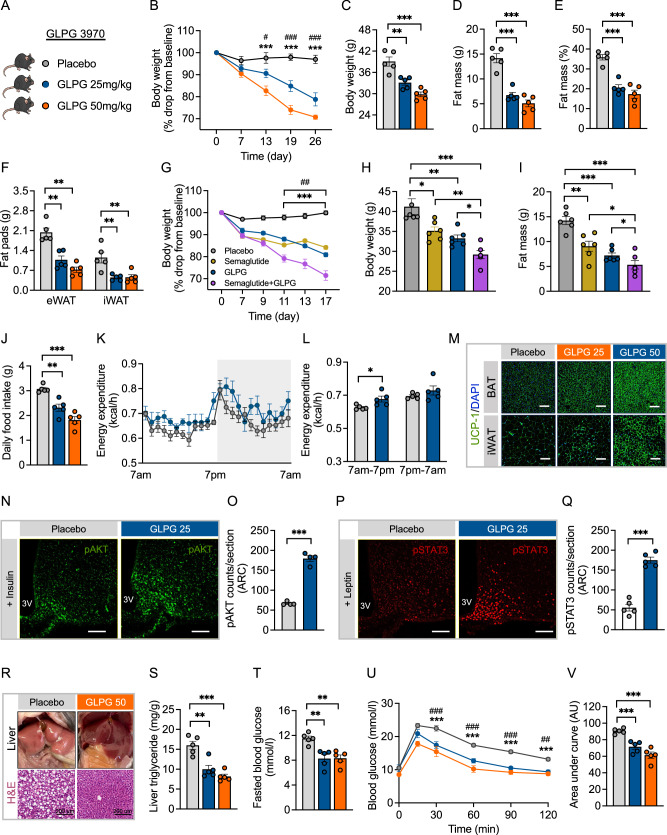
Pharmacological inhibition of SIK3 promotes weight loss and improves metabolic health outcomes. **A** Schematic illustrating three experimental mouse groups: Placebo, GLPG3970 25 mg/kg, and GLPG3970 50 mg/kg, with color-coded symbols indicating the respective treatments. Treatment diagram Created in BioRender. Onda, D. A. (2026) https://BioRender.com/mv2x6id. **B** Body weight % drop from baseline. *n* = 5 biologically independent samples per group. Data are mean ± s.e.m. ^#^*P* < 0.05; ^###^*P*  < 0.001; ***P  < 0.001, by two-way ANOVA with Sidak’s multiple comparisons. (*Placebo vs GLPG3970 50 mg/kg; ^#^Placebo vs GLPG3970 25 mg/kg). **C** Body weight. **D** Fat mass. **E** Fat mass % normalized to body weight. **F** Dissected weights of individual fat depots from eWAT and iWAT in HFD-fed C57BL/6 mice treated with Placebo, GLPG3970 25 mg/kg, or GLPG3970 50 mg/kg orally twice daily for 26 days. *n* = 5 biologically independent samples per group. Data are mean ± s.e.m. **P  < 0.01; ***P  ≤ 0.001, by two-tailed Student’s *t* test. **G** Body weight % drop from baseline. *n* = 6 biologically independent samples per group. Data are mean ± s.e.m. **P  < 0.01; ***P  < 0.001, by two-way ANOVA with Sidak’s multiple comparisons. **H** Body weight and (**I**) Fat mass of HFD-fed mice treated with GLPG3970 25 mg/kg twice every 24 h alone or in combination with 10 nmol/kg Semaglutide once every 48 h. *n* = 6 biologically independent samples per group. Data are mean ± s.e.m. *P < 0.05; **P  < 0.01; ***P < 0.001, by two-tailed Student’s *t* test. **J** Daily food intake. *n *= 5 biologically independent samples per group. Data are mean ± s.e.m. **P = 0.0014; ***P < 0.001, by two-tailed Student’s *t* test. **K**, **L** Energy expenditure. *n* = 5 biologically independent samples per group. Data are mean ± s.e.m. *P = 0.026, by two-tailed Student’s *t* test. **M** UCP-1 immunostaining of BAT and iWAT. Scale bar, 100 µm. *n* = 5 biologically independent samples per group.** N**, **O** Insulin-stimulated p-AKT (Ser 473) in the ARC of Placebo and GLPG3970 treated mice. Scale bar, 100 µm. *n* = 4 biologically independent samples per group. Data are mean ± s.e.m. ***P < 0.001, by two-tailed Student’s *t* test. **P**, **Q** Leptin-stimulated p-STAT3 (Tyr 705) immunostaining in the ARC of Placebo and GLPG3970 treated mice. Scale bar, 100 µm. *n* = 5 biologically independent samples per group. Data are mean ± s.e.m. ***P < 0.001, by two-tailed Student’s* t* test. **R** Representative images and H&E staining of liver tissues. Scale bar, 200 µm.* n* = 5 biologically independent samples per group. **S** Liver triglyceride levels. *n* = 5 biologically independent samples per group. Data are mean ± s.e.m. **P = 0.003; ***P < 0.001, by two-tailed Student’s *t* test. **T** 6 h fasting blood glucose. *n* = 5 biologically independent samples per group. Data are mean ± s.e.m. **P < 0.01, by two-tailed Student’s *t* test. **U**, **V** Glucose tolerance test of HFD-fed Placebo, 25 mg/kg, and 50 mg/kg GLPG3970 treated mice. *n* = 5 biologically independent samples per group. Data are mean ± s.e.m. ***P < 0.001; ^##^*P* < 0.01; ^###^*P* < 0.001, by two-way ANOVA with Tukey’s multiple comparisons. (*Placebo vs GLPG3970 50 mg/kg; ^#^Placebo vs GLPG3970 25 mg/kg). Source data are provided as a Source Data file.

To explore the translational potential of SIK3 inhibition as a strategy for treating metabolic disease, we employed two small-molecule pharmacological approaches, YKL-05-099^81^ and GLPG3970^82^. The preclinical pan-SIK inhibitor YKL-05-099 (SIK3 IC_50_: 30 nM, SIK2 IC_50_: 40 nM, SIK1 IC_50_: 10 nM) demonstrates blood–brain barrier penetrance as confirmed by mass spectrometry (Supplementary Fig. S9A, B). Importantly, YKL-05-099 (18 mg/kg) treatment induced significant body-weight loss in diet-induced obese (DIO) mice compared with placebo (Supplementary Fig. S9C, D), driven by reduced adiposity with preservation of lean mass (Supplementary Fig. S9E–H). This effect was primarily attributable to a significant reduction in food intake (Supplementary Fig. S9I–L), likely mediated by decreased hypothalamic expression of the orexigenic peptides *Agrp* and *Npy* in YKL-05-099–treated mice (Supplementary Fig. S9M, O). Building on these promising findings, we next evaluated GLPG3970, a clinical candidate with enhanced translational relevance. GLPG3970 (Fig. 8A) is an orally bioavailable and highly potent SIK2/3 inhibitor (SIK3 IC_50_: 3.8 nM, SIK2 IC_50_: 7.8 nM, SIK1 IC_50_: 282 nM) with an acceptable safety profile in humans^82^. Using liquid chromatography-tandem mass spectrometry (LC-MS), GLPG3970 was detected in the hypothalamus of mice 2 hours post-oral administration of 50 mg/kg GLPG3970, confirming its ability to cross the blood-brain barrier to inhibit SIK3 in the hypothalamus (Supplementary Fig. S10A). To assess the therapeutic benefits of pharmacological SIK3 inhibition, GLPG3970 was administered orally at doses of 50 mg/kg and 25 mg/kg to DIO mice for up to 26 days (Supplementary Fig. S10B). GLPG3970-treated mice demonstrated significant dose-dependent body weight loss compared to the placebo group (Fig. 8B, C and Supplementary Fig. S10C). Body weight, expressed as a percentage drop from baseline, was significantly reduced in the 50 mg/kg GLPG3970-treated group at 13, 19, and 26 days of treatment (Fig. 8B, C). Most notably, by the 26th day, the 50 mg/kg GLPG3970-treated group exhibited an approximately 30% reduction in body weight, while the 25 mg/kg group showed up to a ~ 20% reduction compared to the placebo group (Fig. 8B, C). This reduction in body weight in GLPG3970-treated DIO mice was predominantly due to fat mass loss (Fig. 8D, E). The reduction in adiposity was further confirmed by a significant decrease in the weights of white adipose tissue depots, specifically the epididymal and inguinal fat pads (Fig. 8F and Supplementary Fig. S10D). While the 50 mg/kg GLPG3970-treated group showed a slight reduction in lean mass ( ~ 4% decrease) compared to placebo, lean mass was comparable between the placebo and 25 mg/kg GLPG3970-treated mice (Supplementary Fig. S10E, F). Notably, lean mass % normalized to body weight was significantly elevated in GLPG3970-treated mice (Supplementary Fig. S10F). The beneficial effect of GLPG3970 was attributed, at least in part, to the inhibition of SIK3 in orexigenic NPY neurons, as mice lacking SIK3 in NPY neurons exhibited only a ~ 10% reduction in body weight compared to controls (Supplementary Fig. S10G).

The current first-line anti-obesity medications belong to a class of drugs known as GLP-1 agonizts^83^. Given that Semaglutide, a type of GLP-1 receptor agonist, and GLPG3970 have different routes of administration and, importantly, mechanisms of action, we next evaluated the weight-lowering effects of the GLP-1 receptor agonist Semaglutide against GLPG3970 in DIO mice. Our study revealed that Semaglutide treatment (10 nmol/kg, administered subcutaneously once every 48 h) resulted in weight loss comparable to that of GLPG3970 (25 mg/kg) (Fig. 8G, H). Importantly, obese mice receiving the combined treatment of Semaglutide and GLPG3970 exhibited a significantly greater reduction in body weight compared to the single treatments, showing an approximately 30% decrease compared to placebo (Fig. 8G, H). In contrast, treatment with either Semaglutide or GLPG3970 alone resulted in a weight loss of only ~ 17% (Fig. 8G, H). This reduction in body weight coincided with a significant decrease in fat (Fig. 8I) and lean mass (Supplementary Fig. S10H) in the Semaglutide and GLPG3970 co-treated group. Taken together, these results suggest that targeting SIK3 may be an effective strategy to promote weight loss and could offer synergistic effects as an adjuvant to current first-line treatments for obesity.

Mechanistically, GLPG3970 treatment suppressed food intake (Fig. 8J), increased energy expenditure (Fig. 8K, L), without affecting ambulatory activity (Supplementary Fig. S10I) or metabolic fuel preferences (Supplementary Fig. S10J). In agreement with the increase in energy expenditure observed in GLPG3970-treated DIO mice, there was a significant decrease in adipocyte size (Supplementary Fig. S10K) and an increase in UCP-1 (Fig. 8M) and TH (Supplementary Fig. S10L) expression in both BAT and iWAT. Pair-feeding placebo mice to match the intake of GLPG3970-treated mice showed that treated mice still exhibited modestly greater weight loss than pair-fed controls (Supplementary Fig. S10M, N), indicating that the effect is likely due to a combination of reduced food intake and increased energy expenditure. Consistent with its reported anti-inflammatory actions, GLPG3970 lowered hypothalamic expression of *Tnfα*, *Il6* and *Ikbkb*, indicating that SIK3 inhibition reduces central inflammatory signaling and may contribute to the associated metabolic benefits (Supplementary Fig. S11A–E). These effects were accompanied by increased central insulin (Fig. 8N, O) and leptin signaling (Fig. 8P, Q) in the ARC of GLPG3970-treated mice compared to placebo. In line with elevated central leptin signaling, GLPG3970 treatment lowered fasting serum leptin (Supplementary Fig. S11F) and co-administration of leptin with GLPG3970 exhibited a marked reduction in body weight that exceeded the effect of either treatment alone (Supplementary Fig. S11G). This weight reduction was accompanied by a significant decrease in fat mass and, to a lesser extent, lean mass (Supplementary Fig. S11H, I). These results suggest that pharmacological inhibition of SIK3 by GLPG3970 increases central insulin and leptin signaling, potentially promoting BAT thermogenesis and iWAT browning and thereby contributing to enhanced energy expenditure. This improved central responsiveness to leptin may support the use of SIK3 inhibition as an adjunctive strategy to restore leptin sensitivity and promote sustained weight loss.

Furthermore, GLPG3970-treated mice also exhibited a series of improvements in metabolic health outcomes. H&E histological examination revealed that lipid deposition in the livers of GLPG3970-treated mice was significantly reduced compared to the placebo group (Fig. 8R). This change in liver morphology coincided with a reduction in liver triglyceride levels (Fig. 8S). These results indicate that inhibition of SIK3 by GLPG3970 alleviates liver steatosis in DIO mice. Additionally, GLPG3970 treatment resulted in significant improvements in glycaemic control. Fasting blood glucose (Fig. 8T) and insulin levels (Supplementary Fig. S11J) were significantly lower in GLPG3970-treated mice compared with the placebo group, suggesting an increase in insulin sensitivity. Consistent with this notion, insulin tolerance tests revealed that GLPG3970-treated DIO mice exhibited markedly improved insulin responsiveness, as evidenced by lower blood glucose levels over the 120-minute duration and when quantified as area under the curve (Supplementary Fig. S11K, L) Furthermore, GLPG3970-treated mice also exhibited significant improvements in whole-body glucose tolerance, underscoring the benefit of pharmacological SIK3 inhibition to improve glycaemic control in DIO mice (Fig. 8U, V). In addition, when challenged with pyruvate, GLPG3970 treated mice showed significantly reduced blood glucose levels indicating reduced hepatic glucose production (Supplementary Fig. S11M, N). Collectively, these findings provide critical preclinical proof-of-concept evidence and establish the therapeutic potential and utility of targeting SIK3 for the treatment of metabolic disease.

## Discussion

While emerging evidence has demonstrated the role of SIKs in regulating various metabolic functions^29,47^, the specific role of hypothalamic SIK3, the predominant SIK isoform in the brain, in controlling body weight and energy balance remains unknown. This study provides evidence that hypothalamic SIK3 signaling regulates energy homeostasis and may contribute to the development of obesity. Selective ablation of SIK3 in orexigenic NPY neurons mitigated diet-induced obesity, and neuron-specific functional rescue via targeted NPY restoration reversed the reduced weight gain in the knockout models, underscoring the causal role of SIK3-NPY signaling within this circuit. Similarly, pharmacological inhibition of SIK3 by GLPG3970 led to reduced body weight and adiposity, improved glycemic control, and alleviated liver steatosis in obese and diabetic mice. The protective effects of SIK3 inhibition were mediated, at least in part, through the enhancement of central leptin and insulin signaling. This, in turn, improved hunger and satiety regulation, increased energy expenditure and reduced hepatic glucose production, collectively resulting in reduced adiposity and improved glycemic control. These findings highlight hypothalamic SIK3 signaling as a critical regulatory node in energy homeostasis and glucose metabolism.

The ARC of the hypothalamus contains two key opposing neuronal populations: orexigenic NPY/AgRP expressing neurons and anorexigenic POMC/CART expressing neurons^18^. These neurons play a critical role in maintaining energy homeostasis by responding to metabolic cues and integrating hormonal signals from peripheral metabolic organs^17^. To fully understand the role of hypothalamic SIK3 in the control of energy metabolism, it is crucial to identify the types of neurons involved in the SIK3-mediated effects on hunger/satiety and glucose regulation. To address this, we generated two mouse models with the selective deletion of SIK3 in NPY or POMC neurons. Notably, these mice were viable and exhibited no overt phenotype with no evidence of developmental or reproductive impairments, in contrast to global SIK3 knockout mice, which exhibit skeletal abnormalities and dwarfism^59^. These models provide an ideal system to study the role of hypothalamic SIK3 in regulating energy metabolism. There were no metabolic or phenotypic differences between wild-type and NPY-neuron-specific SIK3-deficient mice under chow-fed conditions, apart from a modest increase in energy expenditure during the light cycle. However, metabolic differences emerged when the mice were challenged with metabolic stress induced by HFD. We found that SIK3 deficiency in NPY neurons confers resistance to diet-induced obesity, an effect likely driven by reduced NPY expression across the ARC–PVN axis. Consistent with this mechanism, targeted restoration of NPY specifically in the ARC reversed the attenuated weight-gain phenotype in SIK3-deficient mice, reinforcing the central role of SIK3 signaling in orexigenic NPY neurons in governing energy balance. However, deletion of SIK3 in POMC neurons had no metabolic phenotype. The ARC POMC/CART neurons are involved in suppressing feeding and increasing energy expenditure by releasing α-MSH, a product of POMC processing, which activates melanocortin-4 receptors (MC4Rs)^84^. The lack of a metabolic phenotype in POMC-neuron-specific SIK3-deficient mice may be attributed to redundancy and/or the compensatory mechanisms from other SIK3-expressing neuronal populations, such as NPY neurons. NPY neurons also co-express AgRP and the neurotransmitter γ-aminobutyric acid (GABA)^85^, and several mechanisms exist by which NPY/AgRP/GABA neurons can directly and indirectly negatively regulate the activity and function of POMC neurons^86^. Specifically, (1) NPY can act on Y1^87^ and Y2^88^ receptors to inhibit POMC neurons; (2) the co-released GABA acts as an inhibitor of POMC neurons^89^; and (3) AgRP acts as an endogenous antagonist of α-MSH, blocking its action on postsynaptic MC3R and MC4R receptors^90^. Hence, these findings suggest that the NPY system plays a predominant role in mediating the effects of SIK3 on the central regulation of energy homeostasis.

Previous studies have identified SIK3 as a critical regulator of the sleep-wake cycle and sleep need^31,91,92^. Mutations or gain-of-function alleles of SIK3 result in increased non-rapid eye movement (NREM) sleep duration and elevated slow-wave activity through hyperphosphorylation of downstream substrates such as HDAC4^44,93–95^, and SNIPPs^96^. Recent human studies demonstrate that the hSIK3-N783Y mutation is associated with natural short sleep and lower SIK3 kinase activity^97^. This leads to decreased total sleep duration and altered sleep intensity, highlighting its conserved role in regulating sleep-wake homeostasis across species^97^. Importantly, in our study, NPY neuron–specific SIK3 knockout mice did not exhibit overt circadian abnormalities, as evidenced by comparable ambulatory activity patterns between wild-type and knockout mice across both the light and dark cycle. This indicates that the metabolic phenotype observed in our model is not secondary to altered sleep–wake regulation but instead reflects a direct role of SIK3 in NPY neuron–mediated energy homeostasis.

Central leptin and insulin resistance are hallmarks of obesity^74,98,99^. Leptin and insulin have been shown to act on hypothalamic neurons to enhance adaptive thermogenesis and promote WAT browning, serving as key mechanisms for reducing adiposity^53^. Consistent with enhanced central leptin and insulin signaling, our studies provide direct evidence that SIK3 deficiency in NPY neurons promotes sympathetic nerve activity (SNA)-dependent WAT browning and thermogenesis. Several neuronal populations in the hypothalamus have been reported to regulate thermogenesis and WAT browning through the sympathetic nervous system^65,100,101^. For instance, retrograde tracing studies have identified forebrain neurons projecting to BAT, primarily originating from the PVN^102^. The PVN contains inhibitory GABAergic inputs, which play a crucial role in modulating sympathetic outflow. Inhibition of GABAergic receptors in the PVN has been shown to increase sympathetic nerve activity^103–105^. Notably, NPY-expressing neurons in the ARC are one of the key sources of GABAergic input to the PVN^65^. Increased ARC NPY levels or activation of these neurons suppresses BAT thermogenesis, promotes fat storage and contributes significantly to the development of obesity^65^. In contrast, our studies revealed that NPY neuron SIK3-deficient mice exhibit reduced hypothalamic NPY expression and diminished innervation in the ARC and PVN. This was accompanied by a significant elevation in TH, a key enzyme in catecholamine synthesis, in the PVN, inguinal WAT and BAT. Additionally, these mice displayed upregulation of UCP1 and other thermogenic/browning gene markers, resulting in enhanced WAT browning and thermogenesis. This suggests that the absence of SIK3 in NPY neurons disrupts NPY’s inhibitory effects on the PVN, thereby disinhibiting SNS activation. This heightened SNS activity promotes thermogenesis and WAT browning, ultimately leading to reduced adiposity. Collectively, these results underscore the pivotal role of SIK3 in NPY neurons in regulating energy balance and adiposity by modulating leptin and insulin signaling and driving SNS-mediated thermogenesis.

The regulatory mechanisms underlying SIK3’s effects on leptin and insulin signaling are likely mediated through its interaction with well-established downstream effectors, including the transcriptional co-regulators CRTC1 and HDAC5, which regulate the expression of genes involved in appetite control and energy expenditure^36,71,72,106–108^. Consistent with this, our in vitro results demonstrated that overexpression of SIK3 promoted the cytoplasmic sequestration of both HDAC5 and CRTC1 in SK-N-SH-SY5Y neuroblastoma cells. While SK-N-SH-SY5Y cells provided a tractable system for initial mechanistic and localization studies, we acknowledge their limitations in modeling hypothalamic neuron physiology. Future studies using primary hypothalamic neuronal cultures will therefore be warranted to validate and extend these findings in a more physiologically relevant context. Nonetheless, in line with these results, both genetic inhibition of SIK3 in NPY neurons mice and pharmacological inhibition with GLPG3970 promoted nuclear translocation of HDAC5 and CRTC1, reinforcing their role as downstream effectors of SIK3 and contributing to the metabolic phenotype observed in these mice. The role of the CRTC1 signaling pathway in the central regulation of energy balance and glucose homeostasis has been previously demonstrated in animal models. For instance, CRTC1 knockout mice exhibit hyperphagia and an obese phenotype^71^. Additionally, in leptin-deficient *ob/ob* mice, hypothalamic CRTC1 is phosphorylated and inactivated^71^. Similarly, HDAC5 has been shown to be expressed in specific hypothalamic neurons and can be activated by leptin^72^. Furthermore, HDAC5 knockout mice show significant weight gain in response to HFD feeding due to hyperphagia^72^. Mechanistically, HDAC5 regulates the post-translational modification of STAT3, a key downstream effector of leptin signaling, indicating that HDAC5 is a critical component of the leptin signaling pathway^72,109^. These findings suggest impaired HDAC5 and CRTC1 signaling, due to elevated SIK3 activity, may contribute to disrupted central leptin and insulin signaling. Consistent with CRTC1 and HDAC5 being downstream targets of hypothalamic SIK3, our results demonstrate both genetic and pharmacological inhibition of SIK3 increases leptin-induced STAT3 activation and insulin-induced AKT signaling in cells and mice. These findings suggest that inhibiting SIK3 may restore central leptin and insulin sensitivity through effects on CRTC1 and HDAC5, potentially protecting against diet-induced weight gain and promoting weight loss in obesity, although additional studies are needed to establish direct mechanistic evidence.

Our preclinical studies further demonstrated that GLPG3970 significantly decreases body weight and adiposity while preserving lean mass in obese mice. In previous clinical development, the potent and selective SIK2/3 inhibitor GLPG3970, developed by Galapagos NV, was generally well tolerated and demonstrated preliminary efficacy in patients with psoriasis, with > 30% of patients achieving ~ 50 % reduction in the Psoriasis Area and Severity Index (PASI) compared to none in the placebo arm (Galapagos press releases). In a Phase 2a trial in ulcerative colitis patients, some improvements in endoscopic and histologic parameters were observed in GLPG3970-treated patients, although the primary Mayo Clinic Score endpoint did not significantly differ from the placebo group (Galapagos press releases). Similarly, no meaningful clinical benefit was observed in a Phase 2a rheumatoid arthritis trial. Across studies, GLPG3970 was generally safe, with mostly mild to moderate adverse events (Galapagos press releases). These findings support the rationale for SIK2/3 inhibition and provide a translational context for its investigation in metabolic disease such as obesity and its associated complications^82^. Treatment with GLPG3970 also improved glycaemic control and reduced liver steatosis. Mechanistically, our data revealed GLPG3970 crosses the blood-brain barrier and dose-dependently suppresses food intake. These beneficial effects were associated with enhanced central leptin and insulin signaling. Additionally, the potential of SIK3 inhibition as an obesity therapy was reinforced in co-treatment studies. When GLPG3970 was administered alongside the GLP-1 receptor agonist Semaglutide at suboptimal doses, an additive weight-lowering effect was observed. Although NPY neurons represent key neurons through which SIK3 regulates energy balance, the metabolic effects of GLPG3970 cannot be attributed exclusively to SIK3 inhibition within this neuronal population. GLPG3970 was administered orally and reaches both the central nervous system and peripheral organs, raising the likelihood of targeting multiple central and peripheral sites. SIK3 is widely expressed across numerous hypothalamic and extrahypothalamic regions, including the PVN, VMH, DMH, ventral tegmental area and nucleus accumbens^110^, and therefore GLPG3970 may influence broader neural circuits governing appetite and energy expenditure. In addition, non-neuronal cell types such as tanycytes, astrocytes and microglia^111^, all of which express SIK3, may contribute to changes in central nutrient sensing and neuroinflammatory tone. Beyond the CNS, SIK3 is highly expressed in peripheral metabolic tissues, including the liver and skeletal muscle^34^, and pharmacological inhibition by GLPG3970 in these tissues could enhance energy expenditure independently of NPY neuron signaling. Thus, while NPY neuron–specific SIK3 deletion partially blunts the efficacy of GLPG3970, the compound’s potent systemic effects likely reflect integrated actions across multiple neuronal and peripheral cell types rather than a solely NPY-dependent mechanism.

Although GLPG3970 demonstrates strong potency towards SIK3 (IC₅₀ = 3.8 nM), it is also a robust inhibitor of SIK2 (IC₅₀ = 7.8 nM)^82^, and therefore its metabolic effects cannot be attributed solely to SIK3 inhibition. SIK2 is broadly expressed in metabolic tissues and certain hypothalamic neuronal populations, yet its role in central energy regulation remains poorly defined. Given the modest ~2-fold selectivity of GLPG3970 for SIK3 over SIK2^82^, inhibition of SIK2 may also contribute to the observed improvements in diet-induced obesity and glycemic control. While whole-body SIK2 knockout mice show resistance to diet-induced obesity, this does not directly indicate a role in driving weight loss^112^. Together, these findings highlight the complementary contributions of SIK2 and SIK3 and underscore the need for future studies using selective inhibitors or tissue-specific SIK2 or combined SIK2/SIK3 deletion models to define the roles of these kinases in mediating the therapeutic actions of GLPG3970. It is also important to note that while SIK3 is highly conserved between mouse and human, species- and tissue-specific differences in SIK isoform expression pattern, particularly in metabolic tissues, may affect translational relevance.

Although NPY neuron–specific SIK3 deletion protects against diet-induced obesity, it only partially recapitulates the metabolic phenotypes seen in global SIK3 knockout mice^59^. Whole-body SIK3 knockout produces far more pronounced reductions in body weight and adiposity, but interpretation is confounded by perinatal lethality, developmental defects and reduced lean mass in these mice. In contrast, NPY neuron–specific SIK3 knockout mice develop normally and show no abnormalities on chow, allowing clearer attribution of phenotypes emerging under high-fat diet to neuron-specific SIK3 deletion. The incomplete suppression of GLPG3970’s weight-lowering effects in NPY-neuron specific SIK3 knockout mice indicates that NPY neurons are an important but not exclusive site of SIK3 action. Additional hypothalamic and extra-hypothalamic neurons, glial cells and peripheral metabolic tissues likely contribute to the global SIK3 knockout phenotype. These findings reinforce the idea that SIK3 regulates energy balance through multiple central and peripheral pathways and that NPY neurons account for only a part of its systemic function. Future studies using targeted SIK3 deletion across specific neuronal populations, hypothalamic nuclei, and metabolic organs will be needed to define the tissue-specific mechanisms underlying SIK3-dependent control of body weight and energy homeostasis.

In summary, this study provides strong evidence that SIK3 signaling is associated with regulating the hypothalamic control of energy balance and suggests that elevated SIK3 is causally linked to the development of obesity. We show that genetic deletion of SIK3 in orexigenic neurons or treatment with pharmacological inhibitors results in significant therapeutic benefits, including reduced body weight and adiposity, improved glycaemic control and reduced liver steatosis in obese and diabetic mice. Targeting SIK3 represents a promising strategy for developing alternative therapeutics to promote weight loss while preserving lean mass, improve weight management, and reduce obesity-related comorbidities. Furthermore, combining SIK3 inhibition with GLP-1 therapy (e.g., Semaglutide) could offer additive effects to further enhance weight loss and achieve more sustainable, long-term results in weight management. The SIK2/3 inhibitor GLPG3970 used in this study or any SIK3-targeted small-molecule clinical candidate that has demonstrated an acceptable safety profile in humans could represent a promising candidate for treating metabolic diseases.

## METHODS

### Experimental animals

Experiments were performed in accordance with protocols approved by the St Vincent’s Hospital Animal Ethics Committee (AEC No. 017/19, 027/22, 023/23). All mice were housed in a temperature-controlled room of 22 °C with a 12 h light/dark cycle (lights on from 0700-1900 hours) and were allowed free access to food and water. C57BL/6 J mice were sourced from The Walter and Eliza Hall Institute (Victoria, Australia). SIK3^lox/lox^ mice were from N. Tsumaki, Graduate School of Medicine and Frontier Biosciences, Osaka University, Japan. NPY^cre/+^ mice and B6.BKS(D)Lepr db/J heterozygous (*db/+*) mice were obtained from H. Herzog, St Vincent’s Center for Applied Medical Research (Sydney, NSW, Australia). POMC^IRES,Cre/+^ mice^58^ were from B. Lowell, Beth Israel Deaconess Medical Center, Boston, Massachusetts. B6.Cg-Gt(ROSA)26Sor^tm9(CAG-tdTomato)Hze/^J mice were from N. Sims, St. Vincent’s Institute of Medical Research, Australia. To generate NPY and POMC neuron-specific SIK3 deficient mice, SIK3^lox/lox^ were mated with NPY^cre/+^ and POMC^IRES,Cre/+^ mice to generate NPY^cre/+^,SIK3^lox/lox^ and POMC^cre/+^,SIK3^lox/lox^. NPY^cre/+^,SIK3^lox/lox^ and POMC^cre/+^,SIK3^lox/lox^ mice were then subsequently crossed with Gt(ROSA)26Sor^tm9^ to generate NPY^cre/+,Ai9^,SIK3^lox/lox^ and POMC^cre/+,Ai9^,SIK3^lox/lox^ mice. All experimental mice used in this study were on a C57BL/6 J background. Age- and sex- matched mice were used for all experiments. All experimental interventions were performed in male rodents aged 8–10 weeks old unless stated otherwise. Mice were fed a standard chow (19% protein, 4.6% fat and 4.8% crude fiber) or a high-fat diet (23%fat, 45% of total energy from fat; SF04-027; Specialty Feeds, Australia) for the entirety of the experiments except during experiments where fasting is required.

### in vivo Pharmacological studies

C57BL/6 mice were fed a high-fat diet (23% fat; 45% of total energy from fat; SF04-027, Specialty Feeds, Australia) for 10 weeks to induce obesity. Mice received GLPG3970 (synthesized by ADKL Labs, Australia) by oral gavage twice daily between 7-8 a.m. and 4-5 p.m. (administered as a single double dose on weekend) at either 25 mg/kg per dose (50 mg/kg total daily) or 50 mg/kg per dose (100 mg/kg total daily), while control mice were gavaged with an equivalent volume of vehicle [1% DMSO in PBS (v/v)]. For the YKL-05-099 (commercially purchased from MedChem Express, USA) study, mice received YKL-05-099 via oral gavage once daily at 18 mg/kg per dose, while control mice were gavaged with an equivalent volume of vehicle. For the GLPG3970 and Semaglutide (commercially purchased from MedChem Express, USA) combination study, mice were either gavaged with GLPG3970 25 mg/kg per dose twice daily (50 mg/kg total daily) or Semaglutide at 10 nmol/kg subcutaneously once every 48 hours and a combination of both GLPG3970 and Semaglutide for 17 days. For the GLPG3970 and leptin (recombinant, PeproTech/Thermo Fisher Scientific, USA) combination study, mice received GLPG3970 25 mg/kg per dose twice daily (50 mg/kg total daily) orally or leptin 1 μg/g intraperitoneally twice daily and a combination of both GLPG3970 and leptin for 14 days. The treatment duration and metabolic assessments for each experiment are described in the corresponding sections. For pair-feeding studies, 24 h food intake was measured in GLPG3970-treated mice, and a parallel cohort of placebo-treated mice was pair-fed to match the daily caloric intake of the GLPG3970 group, allowing the effects of the drug to be distinguished from those of reduced food consumption.

### Genotyping

DNA was extracted from tail biopsies or tissue samples using 250 µL tail digestion buffer (100 mM Tris-HCl, pH 8.5, 5 mM EDTA, 0.2% (w/v) SDS, 200 mM NaCl) containing 20 μg proteinase K (Meridian Bioscience, USA) and incubated overnight at 55 °C. Supernatant was collected, and genomic DNA was precipitated using 100% isopropanol. DNA was amplified by PCR using MyTaq HS Red Mix (BIO-25048, Meridian Bioscience, USA) using the following primers to detect SIK3 flox (Fwd: primer 5’ CAGCGGAGGAAACTGGATT, Rev: primer 5’ TTAGCCAGGGGTACTCAGGA), SIK3 Del-1f (5′-ACGCACTGATGCCACTGTAG-3′), NPY-cre (Fwd NPY/F3: GGTCGGGGCAAGTGGTCTTC, Rev/Cre GS R2) CCCCAGAAATGCCAGATTACGTAT). POMC-cre (Fwd WT/POMC-3: CATCTGGTCACGTGATAGTGTAA, Rev WT/POMC-5: CGAGCGGCCATTAGGCTT, Rev Cre/Cre3: TGCGAACCTCATCACTCGTT, Ai9 (OIMR9105) 5’ -CTGTTCCTGTACGGCATGG-3’, OIMR9020) 5’-AAGGGAGCTGCAGTGGAGTA-3’ OIMR9103) 5’-GGCATTAAAGCAGCGTATCC-3’ OIMR9021) 5’-CCGAAAATCTGTGGGAAGTC-3’ alleles. Routine genotyping for homozygous *db/db* mice was conducted using TaqMan SNP genotyping assay (AHLIKIN, Thermo Fisher Scientific, USA).

### Metabolic phenotype analysis

Metabolic assessments were conducted in the Bioresources Center (St Vincent’s Hospital Melbourne). Body composition, such as whole-body fat mass and lean mass was measured at 8, and 18 weeks of age, unless otherwise stated, using nuclear Magnetic Resonance Imaging (whole body composition analyzer, EchoMRI, USA). Whole body glucose homeostasis was assessed by injecting glucose (1 g/kg D-glucose, Sigma Aldrich, USA), insulin (1.2 IU/kg Actrapid, Novo Nordisk Pharmaceuticals, Denmark), and pyruvate (1 g/kg, Sigma Aldrich, USA) into the intraperitoneal cavity on 6 h fasted conscious mice at 8- and 18-weeks of age. Blood glucoses were then monitored at 0, 15, 30, 60, 90- and 120 min following intraperitoneal injection. Blood glucose levels were determined using blood collected from tail tip and measured with Accu-Check Performa glucose meter (Roche, Switzerland). Area under the curve (AUC) was calculated using Prism software to determine glucose tolerance. Overnight (16 h) fasted and 30 min re-fed blood were collected by retro-orbital bleeding. Serum was collected and stored at − 20 °C. Plasma insulin levels were measured from serum obtained from the 16 h fasted and 30 mins re-fed mice using a commercially available insulin ELISA assay: mouse specific ultrasensitive insulin ELISA (Alpco Diagnostics, USA). Plasma leptin concentrations were quantified using a high-sensitivity leptin ELISA (Crystal Chem, USA) according to the manufacturer’s instructions. Concentration of triglyceride in liver tissues and serum were measured using the triglyceride colorimetric assay kit (Cayman Chemical, USA), according to the manufacturer’s protocols. Physical activity, food intake and energy expenditure measures were determined using a computer-controlled indirect calorimetry system (Promethion®, Sable Systems, USA). The calorimetry system consists of 16 metabolic cages, reflecting their home cages, which are equipped with indirect open circuit calorimetry, food consumption and activity monitors. Mice were acclimated for 24 h and then monitored for 48–72 h. Energy expenditure and the respiratory exchange ratio were calculated from the gas exchange data (rate of oxygen consumption (VO2) in ml/min and rate of carbon dioxide emission (VCO2) in ml/min). Gas exchange data was used to calculate carbohydrate and fat oxidation rates. Respiratory exchange ratio (RER) reflects the percentage use of fuel substrate at the cellular level at any time (carbohydrate or lipids). RER of 0.7 for fats, 0.8 for proteins, 1.0 for carbohydrates. Ambulatory movements were measured by pedestrian meters, which sums all of the directed ambulatory locomotion of 1 cm/sec or above within the x,y,z beam-brake system, excluding the wheel activity. Feeding was determined by measuring the mass of food consumed. To account for the difference in body composition between the study groups, analysis of energy expenditure was adjusted using ANCOVA using body weight as a covariate.

### Stereotaxic surgery

Animals were anesthetized with 4–5% isofluorane (Pharmachem, Australia) and maintained at 1.5–2% throughout the surgical procedure. After loss of reflexes, animals were fixed in an Ultra Precise Rotational Stereotaxic Instrument (69100, RWD Life Sciences, China). Isoflurane was delivered through a nose cone mounted on the stereotaxic apparatus. The head was cleaned with betadine, and a small incision was made to expose the skull. At defined positions, small holes were made using a dental drill. To over express NPY in NPY neurons, 18-week-old HFD-fed SIK3^lox/lox^ and NPY^Cre/+^;SIK3^lox/lox^ mice were stereotaxically injected with AAV vectors expressing Cre-dependent AAV construct (AAV-CAG > FLEX-NPY-P2A-GFP). Injections were delivered bilaterally into the ARC (coordinates, bregma: anterior-posterior, − 1.70 mm; dorsal-ventral, − 5.85 mm; lateral ± 0.20 mm, 300 nl per side, injected at a rate of 30 nl/min). After the injection, the scalp was sutured. Post-operation animals received analgesia with Carprofen via subcutaneous injection at 5 mg/kg once after surgery. Mice wounds, movement, wellbeing and body weight were continuously monitored at 20 mins, 40 mins, 120 mins, 16 h, 48 h, and 7 days post-surgery.

### Determination of body and bat temperature

To assess changes in BAT activity and thermogenesis, infrared thermography was used to measure dermal temperature changes in the interscapular BAT regions. The FLIRT1010 thermal imaging camera (FLIR Systems, Australia) was mounted onto a tripod at a fixed 70 cm distance to the cage. Mice were anaesthetized intraperitoneally, shaved in the interscapular BAT region. Images were taken in the prone position. Photos of the lumbar and tail regions per mice were also collected for normalization. Temperatures °C were analyzed using the FLIR Research IT Max 4 program (FLIR Systems, Australia). Peak temperatures within the interscapular BAT region were determined and normalized against the peak temperature of the tail region^113^.

### Immunohistochemistry

Mice were anesthetized and transcardially perfused with heparinised saline [10,000 units/l^−1^ porcine heparin in 0.9% (w/v) NaCl] (Sigma Aldrich, USA) followed by 4% (w/v) neutral buffered formalin (Amber Scientific, Australia). Brains were post-fixed for 16 h at 4 °C and moved to 30% sucrose in PBS for 3 days at 4 °C. Fixed brains were frozen in dry ice then embedded in Tissue-Tek O.C.T. compound (ProScieTech, Australia). Using cryostat (Leica CM1860, Germany), brains were sectioned into 30 µM thickness 120 µM apart in the coronal plane throughout the rostral-caudal area of the hypothalamus. Sectioned brains were stored in cryoprotectant (30% ethylene glycol, 20% glycerol in 1x PBS) at − 20 °C. Brain sections were removed from the freezer and allowed to equilibrate at room temperature for 30 minutes. Sections were washed with 1 x PBS 3 × 10 min each on an orbital shaker using low speed at room temperature. Blocked for 2 h in 1x PBS, 0.3% Triton X-100, and 3% normal goat serum (Thermo Fisher Scientific, USA). For detection of SIK3 (CST, 39477, 1:300), NPY (Abcam, Ab30914, 1:500), tyrosine hydroxylase (Abcam, Ab6211, 1:500), pAKT Ser473 (CST, 9271, 1:300), pSTAT3 Tyr705 (CST, 9145, 1:300), and pCRTC1/TORC1 Ser151 (CST, 3359, 1:300), sections were incubated with their respective primary antibodies overnight at 4 °C in 1x PBS with 0.3% Triton X-100 and 3% normal goat serum. After washing with 1 x PBS with 0.3% Triton X-100 for 10 mins 3 times, sections were incubated in goat anti-rabbit-Alexa Fluor 488 (Thermo Fisher Scientific, A32731, 1:500 or 1:1000), goat anti-mouse-Alexa Fluor 488 (Thermo Fisher Scientific, A28175, 1:500), or in goat anti-rabbit-Alexa Fluor 596 (Thermo Fisher Scientific A-11012 1:500), in 1 x PBS with 0.3% Triton X-100 (Merck) and 3% normal goat serum (Thermo Fisher Scientific) for 2 h at room temperature. Sections were mounted with Fluoroshield™ with DAPI (Sigma Aldrich, F6057) mounting media and visualized with Leica Thunder Imager microscope (Leica Microsystems, Germany). Images were captured using LAS-X Life Science Microscope Software (Leica Microsystems, Germany). Images were processed and analyzed using Image J2 Version 2.14/1.54 f (NIH).

For inguinal WAT, epididymal WAT, interscapular BAT and liver immunohistochemistry, tissues were immediately dissected, weighed, and fixed in 4% (w/v) neutral buffered formalin solution for 72 h at room temperature. Tissues were embedded in paraffin. Tissues were sectioned in 5 µM thickness with every 10^th^ section collected. For Haematoxylin and Eosine histology staining, sections were sent to the Melbourne histology platform (The University of Melbourne) for processing. For the detection of UCP-1 and Tyrosine Hydroxylase in ingWAT and BAT, sections were subjected to antigen retrieval. Sections were incubated at room temperature for 2 h with 1x PBS with 0.3% Triton X-100 and 5% normal goat serum were incubated with rabbit anti-UCP1 (CST, [E9Z2V] XP 72298, 1:300), or rabbit anti-tyrosine hydroxylase (Abcam, Ab6211, 1:200) in 1 x PBS with 0.3% Triton X-100 and 5% normal goat serum overnight at 4 °C. Samples were then washed with PBS-T and subsequently incubated with goat-anti-rabbit Alexa Fluor 488 (Thermo Fisher Scientific, A28175, 1:500) for 2 h in 1x PBS with 0.3% Triton X-100 and 5% normal goat serum overnight at room temperature. Sections were washed and mounted with Fluoroshield™ with DAPI (Sigma Aldrich, F6057) mounting media and visualized with Leica Thunder Imager microscope (Leica Microsystems, Germany). Images were captured using LAS-X Life Science Microscope Software (Leica Microsystems, Germany). Images were processed and analyzed using Image J2 Version 2.14/1.54 f (NIH).

### Determination of adipocyte size and number

For histopathological analysis of inguinal WAT and epididymal WAT, tissues were dissected, weighed, and immediately fixed in 4% neutral buffered formalin solution. Adipose tissues were then embedded in paraffin and sectioned at a thickness of 5 µM. Sections were stained with H&E and scanned using an IX81 inverted microscope (Olympus, Japan). Briefly, WAT sections were scanned at 20 x magnifications. Images were then processed and quantified using Image J2 Version 2.14/1.54 f (NIH). WAT Images were calibrated; background was subtracted, and excess noise removed. Images were then subjected thresholding and converted to a binary format to allow labeling and identification of adipose size and shape. Elimination of non-adipose tissue was conducted by excluding adipocyte size below 350 µM^2^. Adipocyte sizes were then calculated as the mean and subjected to size frequency distribution analysis^114^.

### CRTC1 and HDAC5 nuclear-cytoplasmic immunohistochemistry

SK-N-SH-SY5Y (ATCC) were cultured in coverslips. Treated with 10 µM GLPG3970 or DMSO (Sigma Aldrich, USA) for 24 h or control versus SIK3 overexpression. Fasted in 2.8 mM low glucose media for 4 hours. Stimulated with 20 mM glucose for 1 h. Washed with warm PBS. Fixed in 4% neutral buffered formalin (Amber Scientific, Australia) for 15 min at 37 °C. Cells were rinsed with ice cold PBS. Permeabilised in 0.3% Triton-x-100 (Sigma Aldrich, USA) in PBS for 10 min. Incubated in 150 mM glycine in PBS for 10 minutes. Washed with PBS 3 times. Blocked for 2 h in 5% NGS in PBS (Invitrogen, USA). Cells were incubated overnight in the blocking solution with the following primary antibodies: CRTC1 (CST, 2501, 1:300), HDAC5 (CST, 20458, 1:300) at 4 °C. The next day, cells were washed with PBS 3 times and incubated with the following secondary antibodies: goat anti-rabbit AF-488 (Invitrogen, 1:500) or goat anti-rabbit AF-594 (Invitrogen, 1:500) for 2 h at room temperature. Cells were washed with PBS 3 times and mounted with DAPI (F6057 Sigma Aldrich, USA). Images were visualized with Leica Thunder Imager Microscope (Leica Microsystems, Germany). Images were captured using Las-X Life Science Microscope Software (Leica Microsystems, Germany). Nucleus-to-cytoplasm and nucleus-to-perinucleus ratio analyzed using CellProfiler (Version 3.1.9, Broad institute)^115^. At least 800-1000 cells were analyzed per conditions.

### Functional p-AKT and p-STAT3 immunohistochemistry

Mice were fasted for 16 h and injected intraperitoneally with saline or 2.5 IU insulin (Actrapid Novo Nordisk Pharmaceuticals, Denmark) for 15 min, or saline or leptin 1 µg/g leptin (recombinant, PeproTech, USA) for 45 min. Mice were anaesthetized and transcardially perfused as described above after stimulation with 4% neutral buffered formalin. Brains were post-fixed overnight in 4 °C and moved to 30% sucrose (Sigma-Aldrich) in PBS for 3 days. Brains were then frozen in dry ice then embedded in Tissue-Tek O.C.T. compound (ProScieTech, Australia). Using cryostat (Leica CM1860, Germany), brains were sectioned into 30 µm thickness 120 µm apart in the coronal plane throughout the rostral-caudal area of the hypothalamus. Sectioned brains were stored in cryoprotectant (30% ethylene glycol, 20% glycerol in 1x PBS) at − 20 °C. Brain sections were washed in PBS-T(Triton) 3 times for 10 minutes and then incubated in 0.3% glycine in PBS for 10 min, washed 3 times for 5 mins with PBS-T. Then incubated in 0.03% SDS in PBS for 10 min, washed 3 times for 10 min, then blocked in 3% NGS in PBS-T for 2 h at room temperature and incubated overnight with rabbit anti-p-AKT (Ser473) [CST, 9271, 1:300], or rabbit anti-pSTAT3 (Tyr705) [CST, 9145, 1:300] in 3% NGS in PBS-T at 4 °C. The next day, sections were washed 3 times with PBS-T then incubated in goat anti-rabbit-Alexa Fluor 488 (Thermo Fisher Scientific, A32731, 1:500) for 2 h at room temperature. Stained sections were mounted with Fluoroshield™ with DAPI (Sigma-Aldrich, F6057) mounting media and visualized with Leica Thunder Imager microscope (Leica Microsystems, Germany). Images were captured using LAS-X Life Science Microscope Software (Leica Microsystems, Germany). Images were processed and analyzed using Image J2 Version 2.14/1.54 f (NIH).

### Immunoblotting

Whole hypothalamus, inguinal WAT, epididymal WAT, interscapular BAT, and neuroblastoma SK-N-SH-SY5Y cells were homogenized by mechanical scraping or via TissueLyser II (100–120/220–240 V, 50/60 Hz) (QIAGEN, The Netherlands) using 5 mm beads with 50-500 µL cell lysis buffer (50 mM Tris (pH 7.5), 10% glycerol, 50 mM NaF, 5 mM NaPPi, supplemented with 1% (v/v) Triton X-100 and with 1 tablet per 10 mL cOmplete, mini, EDTA-free protease inhibitor cocktail (04693159001 Roche, Switzerland). Approximately 10-30 µg of protein were resolved by 10% SDS-PAGE (Bio-Rad, USA), transferred to Immobilon-FL-PVDF membrane (EMD Millipore, USA). Membranes were blocked in PBS + 0.1% Tween-20 (PBS-T) with 5% non-fat milk for 1 hour at room temperature and immunoblotted with antibodies against SIK3 (CST, 39477, 1:1000), SIK1 (pT182) + SIK2 (pT175) + SIK3 (pT163) (Abcam, Ab199474, 1:1000), p-AKT (Ser473) [CST, 9271, 1:1000], AKT (CST, 2920, 1:1000), UCP1 (CST, [E9Z2V] XP 72298, 1:1000), tyrosine hydroxylase (Abcam, Ab6211, 1:1000), and beta-actin (CST, 4970, 1:1000) and alpha-tubulin (CST, 3873, 1:1000), diluted in PBS-T, overnight at 4 °C shaking. After PBS-T washes, membranes were incubated with anti-rabbit or anti-mouse IgG secondary antibodies fluorescently labeled with IR680 or IR800 dye (LI-COR Biosciences, USA) for 1 h at room temperature. Membranes were then washed and visualized using the Odyssey Infrared Imaging System with densitometry analyses performed with Image Studio Lite software (LI-COR Biosciences, USA).

### Cell culture and transfections

SK-N-SH-SY5Y neuroblastoma cells were kindly gifted by A/Prof. Jon Oakhill (St. Vincent’s Institute of Medical Research, Melbourne, Australia). Cells were maintained in DMEM/F-12 (11320-033, Gibco, USA) supplemented with 10% FBS and penicillin/streptomycin (100 ng/mL). To generate pCDNA5/FRT/TO‑Flag‑SIK3: the coding sequence of SIK3 was amplified by PCR and fused in-frame with a Flag tag (DYKDDDDK) at N-terminus. The Flag-SIK3 insert was cloned into the pcDNA5/FRT/TO vector (Thermo Fisher Scientific, USA) using restriction enzymes. All constructs were sequence-verified to confirm integrity and correct reading frame. For plasmid propagation: NEB-5α competent cells were mixed with ~ 20 ng of pCDNA5/FRT/TO‑Flag‑SIK3 and incubated in ice for 30 min. After gentle mixing, cells were then heat shocked for 45 s at 42 °C and placed straight back in ice. 70 µL of Soc media was then added and incubated at 37 °C for 1 h. Transformed NEB-5α competent cells were then plated on Luria-Bertani broth (LB) agar plates containing ampicillin at 100 ng/µL as a selective marker. A single colony was sub-cultured into 150 mL of LB media and grown at 37 °C shaking in an orbital shaker at 140 rpm for 16 h. 500 µL of cell media was mixed into a sterile Eppendorf tube containing 500 µL of glycerol and stored in − 80 °C. Bacterial cells were harvested by centrifugation at 6000 × *g* for 10 min at 4 °C. Supernatant was discarded and pellet was stored in − 20 °C. Pelleted bacterial cells were subjected to cDNA midi-prep as per HiSpeed Plasmid Midi Kit 25 protocol (QIAGEN, The Netherlands). SK-N-SH-SY5Y cells were subsequently plated in 6 well plates and transfected with 2 μg pCDNA5/FRT/TO-Flag-SIK3 expression construct using FuGENE® HD (Promega) according to the manufacturer’s protocols. After 72 hours of transfection, cells were serum starved for 16 hours and stimulated with insulin 100 nmol insulin (Actrapid, Novo Nordisk Pharmaceuticals, Denmark) for 15 minutes or PBS for control. Basal and stimulated cells were harvested in ice cold whole cell lysis buffer containing 50 mM Tris pH 7.5, 150 mM NaCl, 10% (v/v) glycerol, 50 mM NaF, 5 mM sodium pyrophosphate (NaPPi), 1 mM EDTA, 1 mM EGTA, 1% (v/v) Triton X-100 (Merck) and cOmplete protease inhibitor cocktail (Roche, Switzerland).

### RNA-sequencing (RNA-seq) Analysis

The Seurat object containing the HypoMap single-nucleus RNA sequencing (snRNA-seq) dataset published by Steuernagel et al.^52^, was utilized to identify the expression of *Sik1*, *Sik2*, and *Sik3*. This was performed after sub setting the dataset using the Seurat package^116^ to include only cells from the standard chow diet group.

### Quantitative RT‒PCR

Total RNA of hypothalamus or adipose tissues were extracted using RNAzol reagent (Sigma Aldrich, USA) following the manufacturer’s instructions. Isolated RNA was reverse transcribed into cDNA using the Superscript IV First-strand Synthesis System (Thermo Fisher Scientific, USA) in a 20 µl reaction. Primer annealing and reverse transcription was carried out in a thermocycler (Bio-Rad, USA). Quantitative real-time PCR was conducted using Taqman® gene expression system (Thermo Fisher Scientific, USA). Quantitative real-time PCR was performed using the Light-cycler 480 real-time PCR system (Roche, Switzerland). Reactions were set up in a 384-well PCR plate^117,118^. The following TaqMan® Gene Expression assays were used with FAM-labeled minor groove binder (MGB) probes were used to quantify transcripts: *Sik1* (Mm00440317_m1), *Sik2* (Mm01170976_m1), *Sik3* (Mm00510487_m1), *Ucp1* (Mm01244861_m1), *Dio2* (Mm00515664_m1), *Cidea* (Mm00432554_m1), *Prdm16* (Mm00712556_m1), *Ppargc1a* (Mm01208835_m1), *Pparg* (Mm00440940_m1), *Tmem26* (Mm01173641_m1), *Npy* (Mm01410146_m1), *Agrp* (Mm00475829_g1), and *Pomc* (Mm00435874_m1). Inflammatory and signaling-related genes analyzed included *Tnfα* (Mm00443258_m1), *Ikkβ* (Mm01222247_m1), *Nfκb* (Mm00476361_m1), *Il6* (Mm00446190_m1) and *Il1b* (Mm00434228_m1). The relative gene expression of each gene of interest was performed under the assumption that the binding efficiency of the probes are equal. RT–PCR was performed using a standard cycling protocol comprising an initial denaturation at 95 °C for 10 min, followed by 40 cycles of 95 °C for 15 s and 60 °C for 60 s. Relative transcript abundance was calculated using the 2^–ΔΔCt^ method. *Ppia* (Mm02342430_g) and *Gapdh* (Mm99999915_g1) were used as the housekeeping gene for the normalization of gene expression.

### Hyperinsulinaemic-euglycaemic clamps in conscious freely behaving mice

For hyperinsulinemic-euglycemic clamps, SIK3^lox/lox^ and NPY^cre/+^,SIK3^lox/lox^ mice were fed with HFD for 8 weeks and at 18 weeks of age mice were anaesthetized with isoflurane, subsequently the right jugular vein was catheterized for infusions as previously described^113,119^. Briefly, catheters were attached to an implant button (BMSW25, RWD Life Sciences, China). Implant buttons were sealed with a cap allowing for mouse to be housed in groups. Catheters were kept patent through daily flushing of 40 µL heparinized saline. On the day of the procedure, mice were fasted at 7:00 for 3.5 hours, then a primed (1 min, 1.25 µCi min^−1^) continuous infusion (0.05 µCi min^−1^) of [3-^3^H] glucose (NET331A001MC, PerkinElmer, USA) was administered to assess whole body glucose turnover^119^. After 90 minutes, mice were administered with 40 mU kg^−1^ bolus of insulin over 10 minutes followed by continuous insulin infusion at 4 mU kg^−1^ min^−1^ in gelofusine. Euglycemia detected at around ~8-10 mM blood glucose was maintained by a variable infusion of 30% glucose solution. Throughout the steady-state conditions (rate of appearance [Ra] = rate of disappearance [Rd]) and at 80, 90-, 100-, 110-, and 120-minute time points tail blood samples were collected to measure Ra and Rd as described above. At 120 minutes, a 13 µCi bolus of [^14^C]−2-deoxy-D-glucose (NEC495A250UC, PerkinElmer) was injected via the jugular vein. Blood samples were collected at 122, 125-, 135-, 145-, and 155-minute time points. Tissues were collected to assess glucose uptake^113,119^.

### Pancreatic islet isolation and glucose stimulated insulin secretion (GSIS) ex vivo

Mouse islets were isolated from male NPY^cre/+^,SIK3^lox/lox^ and SIK3^lox/lox^ mice. Briefly, mice were euthanized, and the abdominal cavity was opened to expose the pancreas and bile duct. The bile duct was cannulated with a 30‑gauge needle, and 3 ml of collagenase P solution (0.3 mg/ml) (Sigma Aldrich, USA) was slowly injected to inflate the pancreas. Excised pancreata were incubated in prewarmed Hanks’ Balanced Salt Solution (HBSS) containing CaCl_2_ and HEPES at 37 °C for 12 min to digest exocrine tissue, with digestion times optimized per collagenase batch. Pancreata were then placed on ice, mechanically disrupted by shaking, and filtered through a 500 µm mesh to remove large tissue fragments. The filtrate was layered onto a discontinuous density gradient of Histopaque®‑1077 and RPMI 1640 medium (Sigma Aldrich, USA) and centrifuged at 1000 × *g* for 15 min at room temperature without brake. Islets were collected from the interface, washed twice with RPMI, and hand‑picked under a stereomicroscope using low‑retention pipette tips to ensure purity. The isolated mouse islets were cultured in Connaught Medical Research Laboratories (CMRL) 1066 medium (Invitrogen, Life Technologies, USA) supplemented with 10% fetal bovine serum (ThermoFisher Scientific), 100 U/ml penicillin, 100 mg/ml streptomycin (Thermo Fisher Scientific, USA), and 2 mmol/L l-glutamine (Thermo Fisher Scientific, USA). The isolated islets were incubated in a 37 °C, 5% CO2 humidified incubator for 60 min before use in downstream assays. GSIS was measured in isolated islets as previously described^120,121^. Briefly, groups of fifteen size-matched islets were preincubated in HEPES-buffered KREBS buffer containing 0.2% BSA and 2.8 mmol/L glucose for 1 h. Islets were then transferred to KREBS buffer containing either 2.8 mmol/L or 20 mmol/L glucose for an additional 1 h incubation at 37 °C. Culture media were collected, and insulin secretion was measured using a mouse insulin ELISA kit (ALPCO Diagnostics, USA).

### Liquid chromatography-tandem mass spectrometry (LC-MS/MS)

Wild-type C57BL/6 J mice were orally administered vehicle (DMSO) or GLPG3970 (50 mg/kg), or vehicle (Tris–HCl) or YKL-05-099 (18 mg/kg) 2 h before tissue collection. Mice were perfused with phosphate-buffered saline (PBS) by cardiac perfusion, after which the whole hypothalami were rapidly dissected. Tissues were homogenized in 300 µl of 75% methanol using a TissueLyser LT (QIAGEN, The Netherlands) at 50 Hz for 4 min. Homogenates were centrifuged at 12,000 × *g* for 5 min and the resulting supernatants were collected for liquid chromatography–mass spectrometry analysis. LC–MS/MS analyses were performed using a Nexera LC-30AD HPLC system (Shimadzu, Japan) coupled to a 5500 QTRAP mass spectrometer (Sciex, USA). Method optimization and compound detection were conducted by Dr Dingyi Yu at the St Vincent’s Institute of Medical Research. Parent standard solutions of GLPG3970 and YKL-05-099 were prepared in DMSO and serially diluted in Milli-Q water to generate linear calibration curves. Chromatographic separation was performed using an ACQUITY UPLC BEH C18 column (130 Å, 1.7 µm, 2.1 × 100 mm; Waters, USA) maintained at 40 °C. Reversed-phase chromatography was carried out with buffer A (90% methanol with 0.1% formic acid in Milli-Q water) and buffer B (0.1% formic acid in Milli-Q water) at a flow rate of 200 µl min⁻¹. The gradient program consisted of 20% B for 2 min, a linear increase to 90% B over 5 min, followed by a 5 min hold at 90% B. Samples and standards (10 µl) were injected using an autosampler maintained at 4 °C. Mass spectrometric identification and quantification were performed using multiple reaction monitoring (MRM) in positive ion mode. The monitored precursor-to-product ion transitions were as follows: GLPG3970, m/z 505.5 → 113.6 (quantifier, CE 60 V), m/z 505.5 → 70.0 (qualifier, CE 78 V) and m/z 505.5 → 84.1 (qualifier, CE 78 V); YKL-05-099, m/z 601.3 → 188.0 (quantifier, CE 45 V), m/z 601.1 → 434.3 (qualifier, CE 65 V) and m/z 601.1 → 348.0 (qualifier, CE 78 V). The dwell time was set to 50 ms for all transitions. The declustering potential and entrance potential were set to 120 V and 10 V, respectively. Ion source parameters were as follows: curtain gas 25 psi, ion spray voltage 5500 V, source temperature 400 °C, ion source gas 1 at 35 psi and ion source gas 2 at 35 psi. Data acquisition and analysis were performed using Analyst v1.6 software (Sciex, USA).

### RNAscope® In Situ Hybridization

For NPY and SIK3 mRNA detection via RNAscope, RNAscope duplex chromogenic assay (Advanced Cell Diagnostics, Inc, USA) — an in situ hybridization (ISH) technique to visualize multiple cellular mRNA targets in fresh frozen tissues to detect co expression of *Npy* and *Sik3* in adult mouse brain as described previously^122^. Tissues from mice at 15-16 weeks of age were used. Tissue sections (25 µm) were cut on a cryostat and thaw-mounted on Superfrost® slides (Menzel-Glaser Braunschweig, Germany), and dual labeled for the mRNA of *Npy* (#313321-C2) and *Sik3* (#526431) using RNAScope® 2.5 Duplex Detection kit (#322430) following the manufacturer’s protocol (Advanced Cell Diagnostics, Inc, USA).

### Quantification and statistical analysis

Data are presented as mean ± s.e.m. and were analyzed using GraphPad Prism 9. Immunofluorescence staining was visualized using a Leica THUNDER Imager Microscope (Leica Microsystems, Germany), and images were acquired using LAS X Life Science Microscope Software (Leica Microsystems, Germany). Schematic figures were Created in BioRender. Onda, D. A. (2026) https://BioRender.com/mv2x6id. Image processing and analysis were performed using ImageJ2 Version 2.14/1.54 f (NIH). For comparisons between two groups (for example, SIK3^lox/lox^ and SIK3^lox/lox^,NPY^cre/+^ or SIK3^lox/lox^,POMC^cre/^), one- or two-tailed Student’s t-tests were used as appropriate unless otherwise specified. For experiments involving two independent variables (for example, genotype and/or treatment), data were analysed using ordinary two-way ANOVA followed by Sidak’s or Tukey’s multiple-comparison tests. Energy expenditure data were adjusted for differences in body composition using analysis of covariance (ANCOVA) with total body mass as a covariate. Correlations were assessed using Spearman’s rank correlation coefficient, and simple linear regression was applied where appropriate. Statistical significance was defined as *P* ≤ 0.05 (*P ≤ 0.05, **P ≤ 0.01, ***P ≤ 0.001). Exact sample sizes (n) and statistical tests used are indicated in the corresponding figure legends.

### Reporting summary

Further information on research design is available in the Nature Portfolio Reporting Summary linked to this article.

## Supplementary information

Supplementary Information

Reporting Summary

Transparent Peer Review file

## Source data

Source Data

## Data Availability

The data supporting the findings of this study are available within the paper and its Supplementary Information files. Source data underlying all figures are provided with this paper. Source data are provided in this paper.

## Materials availability

This study did not generate new unique reagents.

## Code availability

This study did not generate any unique datasets or code.
