# Supplementary material for: Targeting hypothalamic SIK3 to promote weight loss and improve glycemic control in mice
Source: Nat Commun. 2026 Jun 2;17:8432. doi: 10.1038/s41467-026-73649-1 (PMC13478199; doi:10.1038/s41467-026-73649-1)
Supplement: Supplementary file 1 [file 41467_2026_73649_MOESM1_ESM.pdf]

Supplementary Information

Targeting Hypothalamic SIK3 to Promote Weight Loss and Improve Glycemic Control in Mice

Danise Ann Onda[1], Chieh-Hsin Yang[1], Callen Goldsmith[2], Cait A. Beddows[2], Rui Qi Teo[2], Lei Zhang[3], XiaoZhuo Yuan[1], Yifei Zhu[1], Man KS Lee[4], Ashley J Ovens[5], Dingyi Yu[6], Kei Sakamoto[7], John W Scott[6,8], Andrew J Murphy[9], Noriyuki Tsumaki[10], Herbert Herzog[3], Garron T Dodd[2,*], Kim Loh[1,11,12,*,#]

[1]Diabetes and Metabolic Disease, St. Vincent's Institute of Medical Research, Fitzroy, Victoria, Australia.
[2]Department of Anatomy and Physiology, The University of Melbourne, Melbourne, Victoria, Australia.
[3]St Vincent's Center for Applied Medical Research, Sydney, NSW, Australia.
[4]Division of Immunometabolism, Baker Heart and Diabetes Institute, Melbourne, Australia.
[5]Protein Engineering in Immunity & Metabolism, St. Vincent's Institute of Medical Research, Fitzroy, Victoria, Australia.
[6]St. Vincent's Institute of Medical Research, Fitzroy, Victoria, Australia.
[7]Novo Nordisk Foundation Center for Basic Metabolic Research, University of Copenhagen, Copenhagen 2200, Denmark.
[8]Drug Discovery Biology, Monash Institute of Pharmaceutical Sciences, Melbourne, Victoria, Australia.
[9]Division of Immunometabolism, Baker Heart and Diabetes Institute, Melbourne, Australia; Department of Diabetes, Monash University, Melbourne, Australia; Department of Cardiometabolic Health, University of Melbourne, Melbourne, Australia; Department of Medicine, University of Melbourne, Melbourne, Australia.
[10]Department of Tissue Biochemistry, Graduate School of Medicine and Frontier Biosciences, Osaka University, Osaka, Japan.
[11]Department of Medicine, University of Melbourne, Fitzroy, Victoria, Australia.
[12]Mary MacKillop Institute for Health Research, Australian Catholic University, Victoria, Australia.
*These authors contributed equally.

[#]Corresponding author:
Kim Loh, PhD
Diabetes and Metabolic Disease Laboratory
St Vincent's Institute of Medical Research
Email: kloh@svi.edu.au

Supplemental Figures and Figure Legends

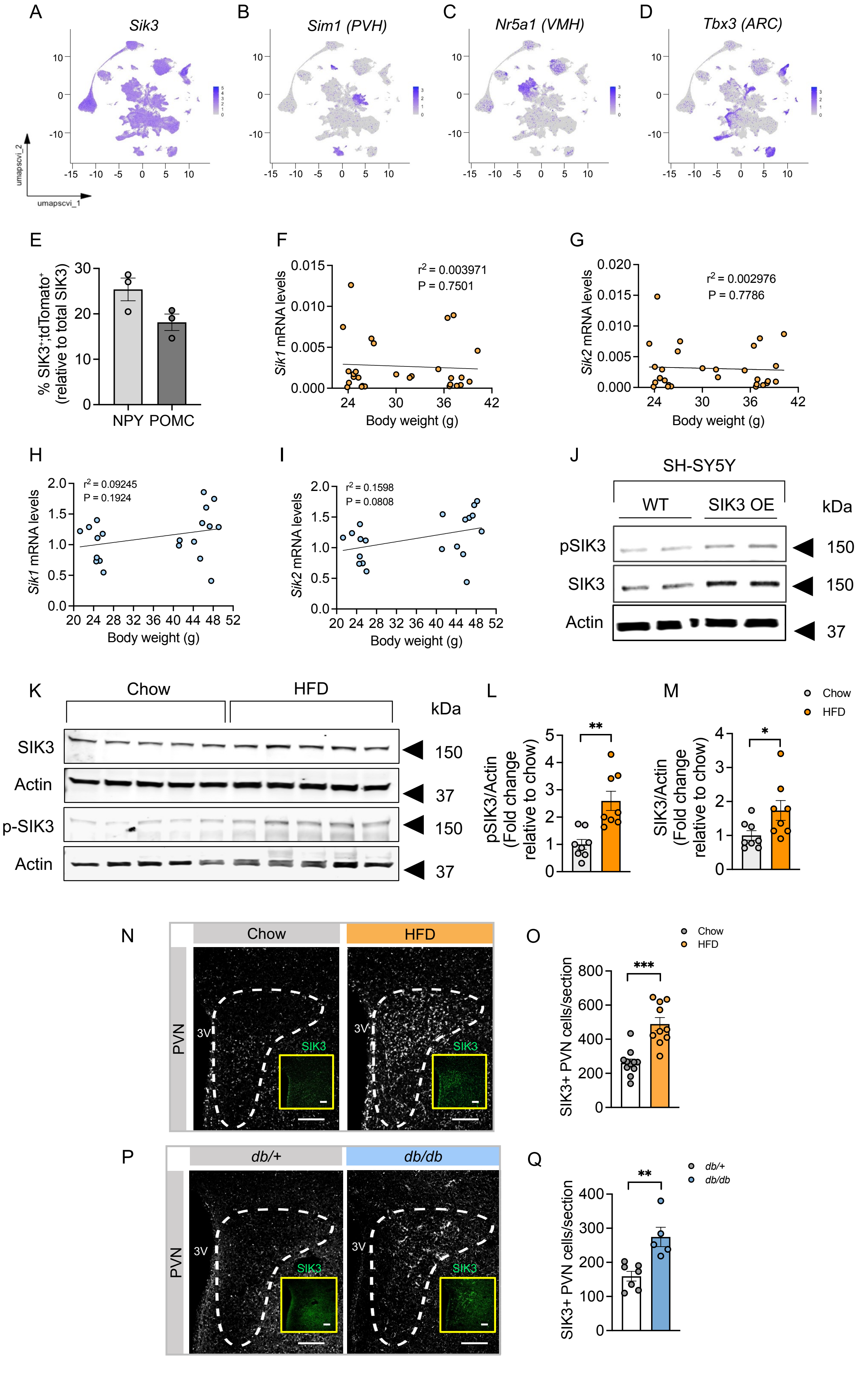

A
Sik3
B
Sim1 (PVH)
C
Nr5a1 (VMH)
D
Tbx3 (ARC)
umapscvi_2
umapscvi_1
E
% SIK3+;tdTomato+ (relative to total SIK3)
NPY
POMC
F
r2 = 0.003971
P = 0.7501
Sik1 mRNA levels
Body weight (g)
G
r2 = 0.002976
P = 0.7786
Sik2 mRNA levels
Body weight (g)
H
r2 = 0.09245
P = 0.1924
Sik1 mRNA levels
Body weight (g)
I
r2 = 0.1598
P = 0.0808
Sik2 mRNA levels
Body weight (g)
J
SH-SY5Y
WT
SIK3 OE
kDa
pSIK3
SIK3
Actin
150
150
37
K
Chow
HFD
kDa
SIK3
Actin
p-SIK3
Actin
150
37
150
37
L
pSIK3/Actin (Fold change relative to chow)
**
M
SIK3/Actin (Fold change relative to chow)
*
Chow
HFD
N
Chow
HFD
PVN
3V
SIK3
O
Chow
HFD
SIK3+ PVN cells/section
***
P
db/+
db/db
PVN
3V
SIK3
Q
db/+
db/db
SIK3+ PVN cells/section
**

Supplemental Figure 1. Identification of SIK3 positive cells in the other hypothalamic cells and nuclei of the murine brain.

(A) UMAP plot analysis of *Sik3* within different neuronal subtype populations in the hypothalamus. (B) (C) (D) *Sik3* is also highly expressed in neuronal cells expressing *Sim1*, *Nr5a1*, and *Tbx3*, which are established markers for neurons within the PVN, VMH, and ARC. (E) Percentage of $SIK3^+$ and tdTomato-NPY and POMC neurons (relative to total SIK3) in the ARC of chow-fed male $NPY^{cre/+;Ai9}$,$SIK3^{+/+}$ and $POMC^{cre/+;Ai9}$,$SIK3^{+/+}$. Scale bar, 100 μm. n = 3 biologically independent samples per group. (F) (G) Correlation analysis of *Sik1* and *Sik2* expression against changes in body weight in chow- and HFD-fed mice. n = 28 biologically independent samples per group. Data are mean ± s.e.m. $P > 0.05$, by simple linear regression. (H) (I) Correlation analysis of *Sik1* and *Sik2* expression against changes in body weight in lean (*db/*+) and obese (*db/db*) mice. n = 20 biologically independent samples per group. Data are mean ± s.e.m. $P > 0.05$, by simple linear regression. (J) Immunoblot of phosphorylated SIK3 and total SIK3 in WT and SIK3 overexpressing SK-N-SH-SY5Y neuroblastoma cells to validate specificity of pan p-SIK antibody. (K) Representative immunoblots of phosphorylated SIK3 and total SIK3 in chow and HFD hypothalamus. (L) Densitometry analysis of phosphorylated SIK3 in the hypothalamus of chow and HFD-fed mice. (M) Densitometry analysis of total SIK3 in the hypothalamus of chow and HFD-fed mice. n = 8 biologically independent samples per group. Data are mean ± s.e.m. $^{**}P = 0.002$ for (L); $^{*}P = 0.046$ for (M), by two-tailed Student's t-test. (N) (O) Immunostaining analysis of SIK3 in the PVN of chow and HFD-fed mice. Scale bar, 100 μm. n = 10-11 biologically independent samples per group. Data are mean ± s.e.m. $^{***}P < 0.001$, by two-tailed Student's t-test. (P) (Q) Immunostaining analysis of SIK3 in the PVN of *db/*+ and *db/db* mice. Scale bar, 100 μm. n = 5-7 biologically independent samples per group. Data are mean ± s.e.m. $^{**}P = 0.002$, by two-tailed Student's t-test. Source data are provided as a Source Data file.

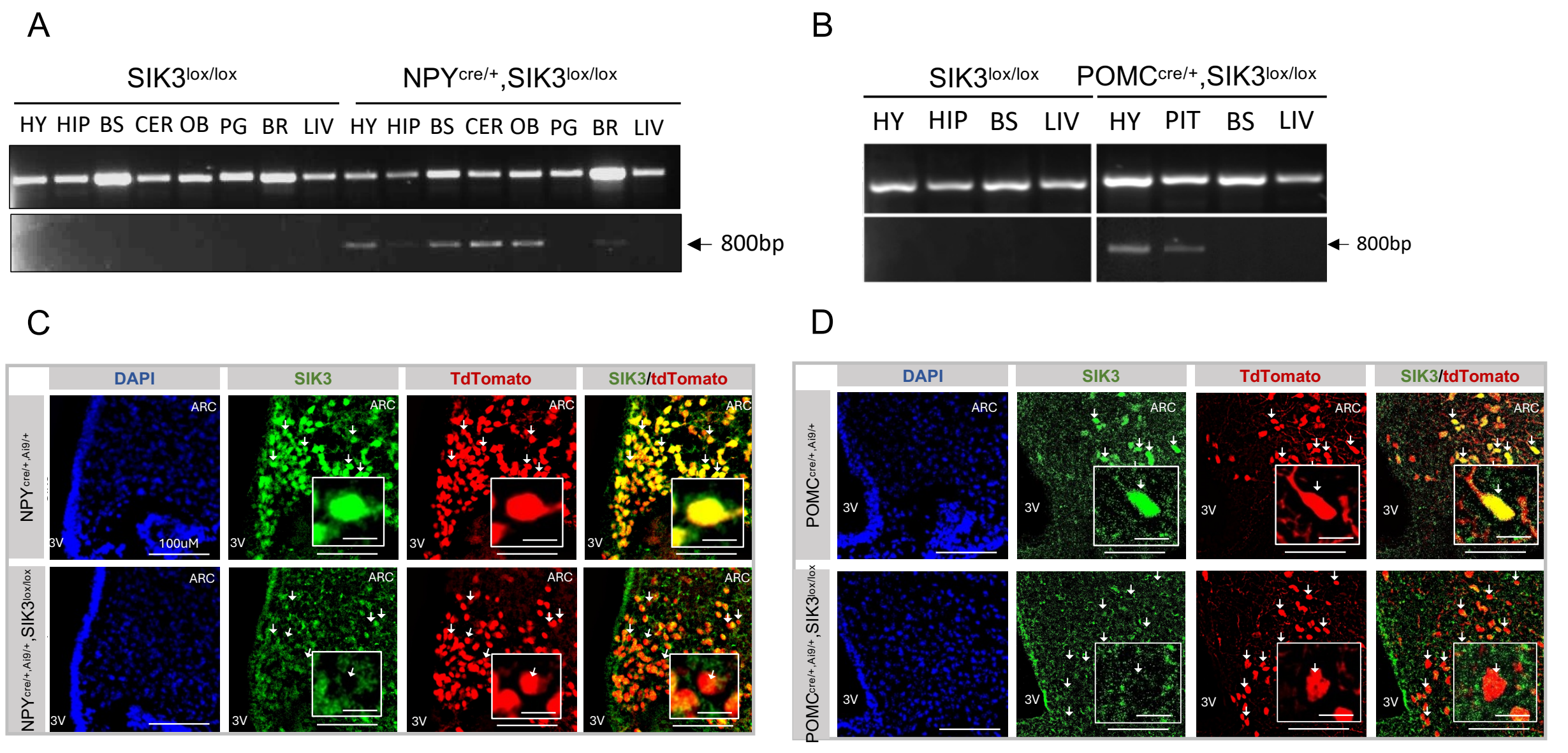

E

Breeding pair	N	Litter size	Genotype frequency: SIK3lox/lox	Genotype frequency: SIK3lox/lox;NPYCre/+	Male offspring	Female offsprings
Female SIK3lox/lox X Male SIK3lox/lox;NPYCre/+	7 breeding pairs	8.57 ± 0.57	4	4.57	53.33333333	46.66666667
Male SIK3lox/lox X Female SIK3lox/lox;NPYCre/+	7 breeding pairs	8.14 ± 0.50	4.2	3.8	59.64912281	40.35087719

F

Breeding pair	N	Litter size	Genotype frequency: SIK3lox/lox	Genotype frequency: SIK3lox/lox;POMC Cre/+	Male offspring	Female offspring
Female SIK3lox/lox X Male SIK3lox/lox;POMCCre/+	5 breeding pairs	8.57 ± 0.57	4	4.57	53.3333333	46.6666667
Male SIK3lox/lox X Female SIK3lox/lox;POMCCre/+	5 breeding pairs	8.14 ± 0.50	4.2	3.8	59.64912	40.3508772

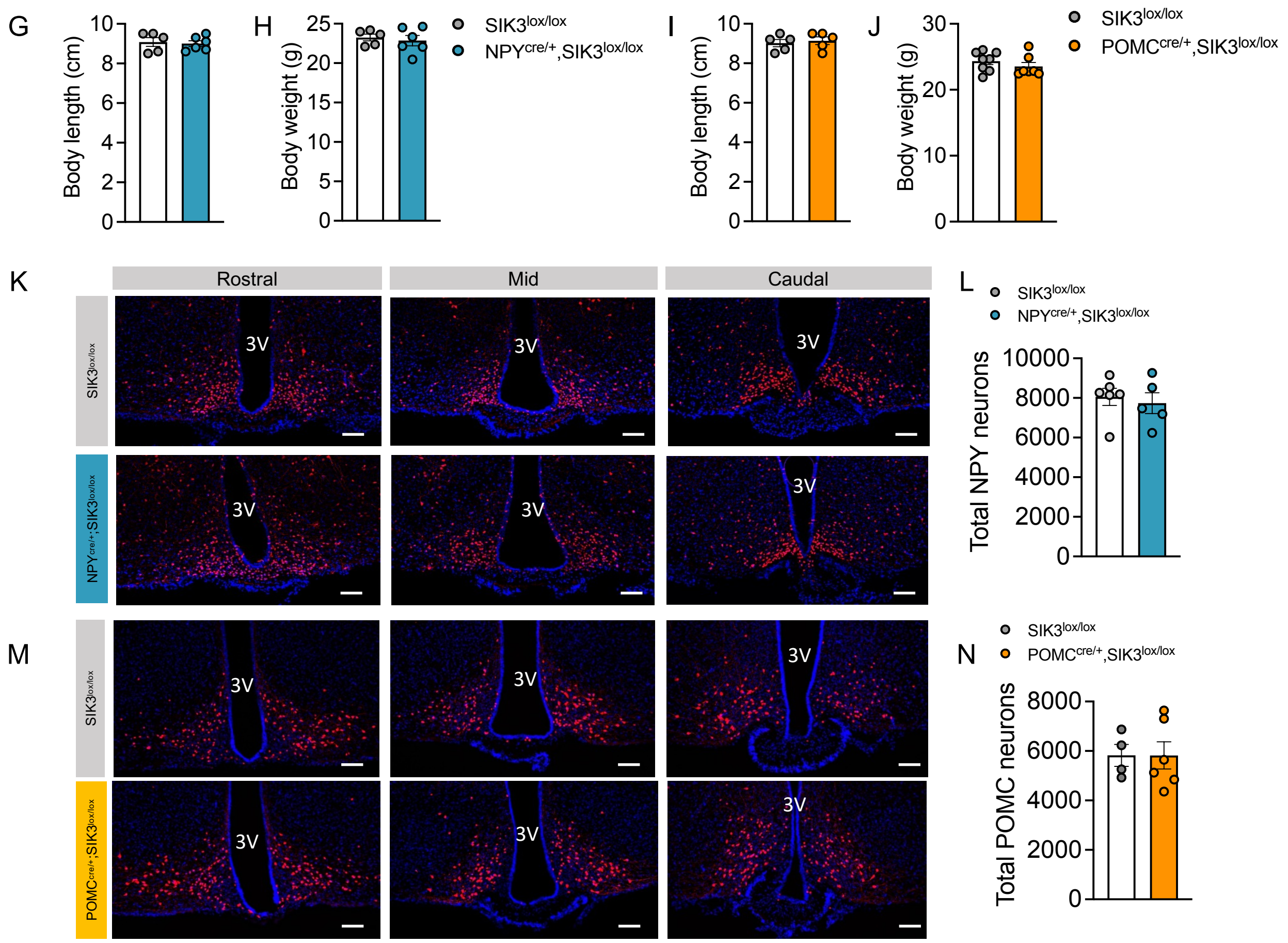

Supplemental Figure 2. Generation and validation of mice lacking SIK3 in NPY and POMC neurons

Recombination of the floxed SIK3 allele with (A) NPY$^{cre/+}$ and (B) POMC$^{cre/+}$ in hypothalamus (HY), hippocampus (HI), brain stem (BS), cerebrum (CER), olfactory bulb (OB), pituitary gland (PG), whole brain (BR), and liver (LIV) were assessed by PCR. (C) (D) Immunostaining analysis showing reduced colocalization of SIK3 (green) and NPY (Red) protein expression in the hypothalamus of NPY$^{cre/+;Ai9}$,SIK3$^{lox/lox}$ and POMC$^{cre/+;Ai9}$,SIK3$^{lox/lox}$. Scale bar, 100 μm and 12.5 μm (zoomed-in images). n = 3 biologically independent samples per group. (E) Breeding, genotype frequency, litter size, mortality, and percentage of male and female offspring born from SIK3$^{lox/lox}$ and NPY$^{cre/+}$,SIK3$^{lox/lox}$ mice. (F) Breeding, genotype frequency, litter size, mortality, and percentage of male and female offspring born from SIK3$^{lox/lox}$ and POMC$^{cre/+}$,SIK3$^{lox/lox}$ mice. n = 5-7 breeding pairs per group. (G) Body length (H) Body weight of SIK3$^{lox/lox}$ and NPY$^{cre/+}$,SIK3$^{lox/lox}$ mice. n = 5-6 biologically independent samples per group. Data are mean ± s.e.m. $P > 0.05$, by two-tailed Student's t-test. (I) Body length (J) Body weight of SIK3$^{lox/lox}$ and POMC$^{cre/+}$,SIK3$^{lox/lox}$ mice. n = 5-8 biologically independent samples per group. Data are mean ± s.e.m. $P > 0.05$, by two-tailed Student's t-test. (K) Representative sections of rostral, mid, and caudal ARC from NPY$^{cre/Ai9}$;SIK3$^{lox/lox}$ and NPY$^{cre/Ai9}$;SIK3$^{+/+}$. (L) Counts of NPY neurons within the ARC of NPY$^{cre/Ai9}$;SIK3$^{lox/lox}$ and NPY$^{cre/Ai9}$;SIK3$^{+/+}$. Scale bars: 100 μm. n = 5-6 biologically independent samples per group. Data are mean ± s.e.m. $P > 0.05$, by two-tailed Student's t-test. (M) Representative sections of rostral, mid, and caudal ARC from POMC$^{cre/Ai9}$;SIK3$^{lox/lox}$ and POMC$^{cre/Ai9}$;SIK3$^{+/+}$. (N) Counts of POMC neurons within the ARC of POMC$^{cre/Ai9}$;SIK3$^{lox/lox}$ and POMC$^{cre/Ai9}$;SIK3$^{+/+}$. Scale bars: 100 μm. n = 4-6 biologically independent samples per group. Data are mean ± s.e.m. $P > 0.05$, by two-tailed Student's t-test. Source data are provided as a Source Data file.

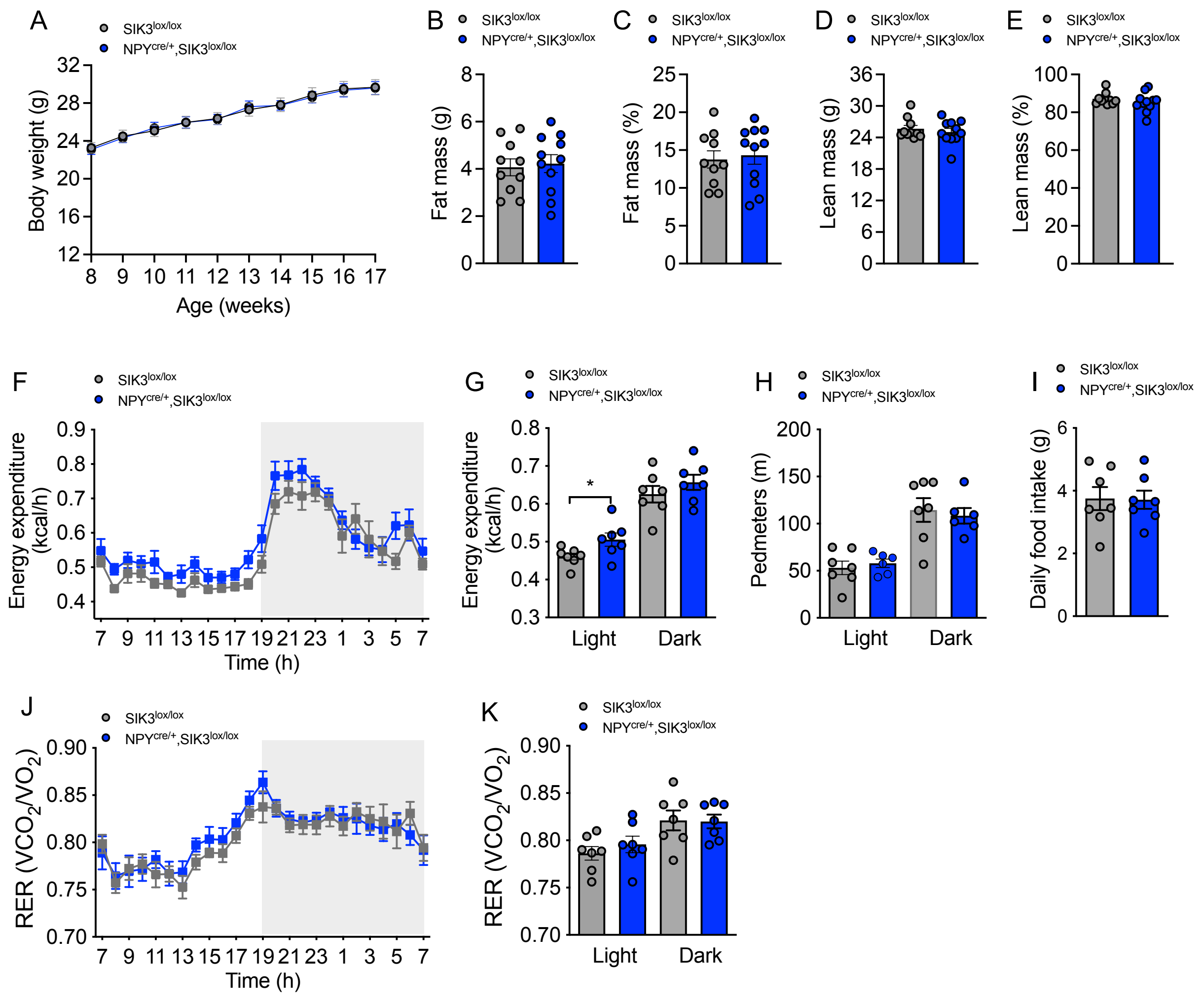

A
SIK3lox/lox
NPYcre/+,SIK3lox/lox
Body weight (g)
Age (weeks)
B
Fat mass (g)
C
Fat mass (%)
D
Lean mass (g)
E
Lean mass (%)
F
Energy expenditure (kcal/h)
Time (h)
G
Energy expenditure (kcal/h)
Light
Dark
H
Pedmeters (m)
I
Daily food intake (g)
J
RER (VCO2/VO2)
K

Supplemental Figure 3. Selective deletion of SIK3 in NPY neurons under SCD-fed conditions.

(A) Weekly body weight monitoring. n = 10-11 biologically independent samples per group. Data are mean ± s.e.m. $P > 0.05$, by two-way ANOVA with Sidak's multiple comparisons. (B) Fat mass (C) Fat mass % normalized to body weight (D) Lean mass (E) Lean mass % normalized to body weight of SCD-fed $SIK3^{lox/lox}$ and $NPY^{cre/+}$,$SIK3^{lox/lox}$ mice. n = 10-11 biologically independent samples per group. Data are mean ± s.e.m. $P > 0.05$, by two-tailed Student's t-test. (F) (G) Energy expenditure. (H) Pedometers. (I) Daily food intake. (J) (K) Respiratory exchange ratio (RER). n = 6-7 biologically independent samples per group. Data are mean ± s.e.m. $^{*}P = 0.039$ for (G), by two-tailed Student's t-test. Source data are provided as a Source Data file.

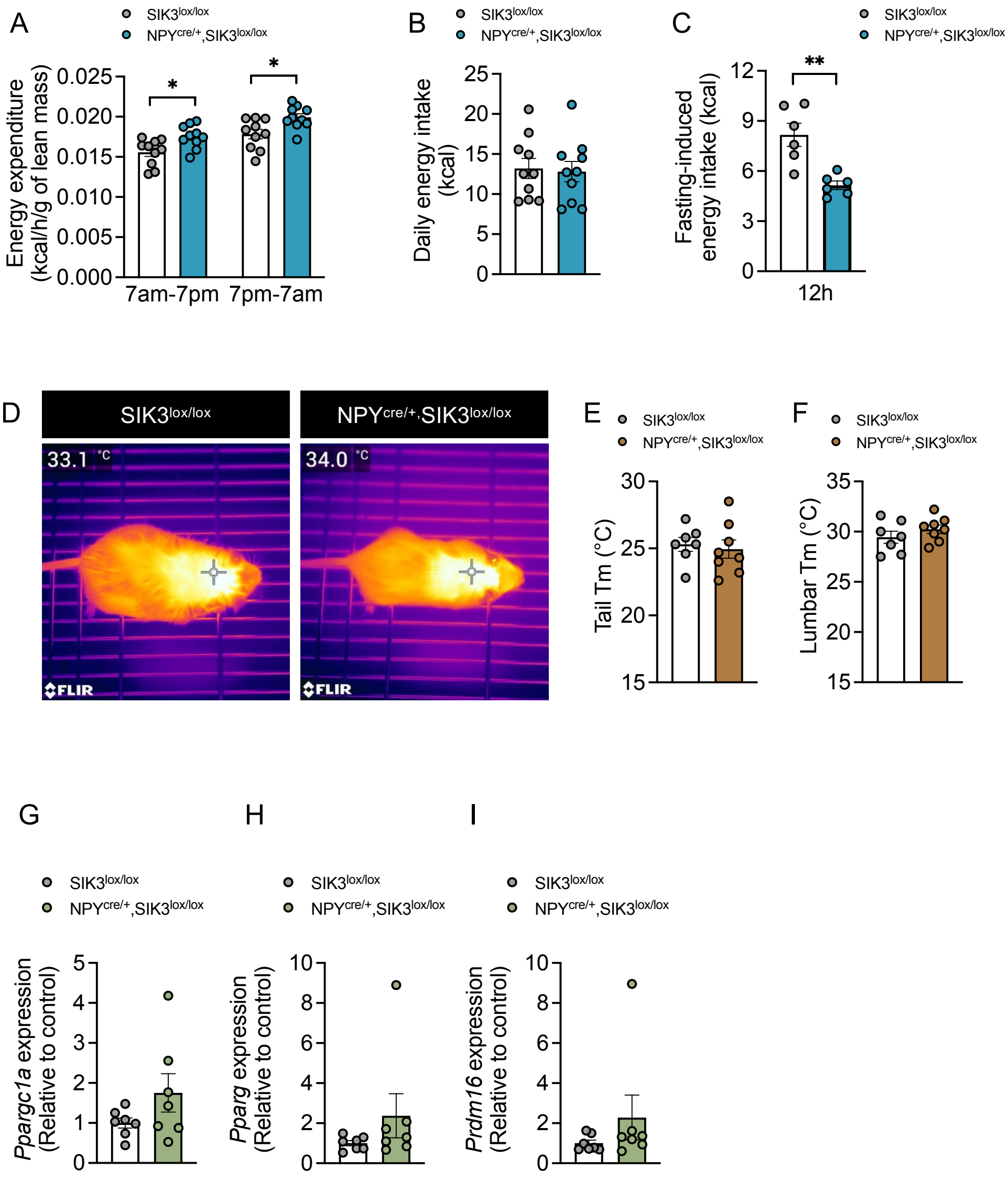
A
SIK3$^{lox/lox}$
NPY$^{cre/+}$,SIK3$^{lox/lox}$
Energy expenditure (kcal/h/g of lean mass)
0.025
0.020
0.015
0.010
0.005
0.000
*
*
7am-7pm
7pm-7am
B
SIK3$^{lox/lox}$
NPY$^{cre/+}$,SIK3$^{lox/lox}$
Daily energy intake (kcal)
25
20
15
10
5
0
C
SIK3$^{lox/lox}$
NPY$^{cre/+}$,SIK3$^{lox/lox}$
Fasting-induced energy intake (kcal)
12
9
6
3
0
**
12h
D
SIK3$^{lox/lox}$
NPY$^{cre/+}$,SIK3$^{lox/lox}$
33.1 °C
34.0 °C
FLIR
FLIR
E
SIK3$^{lox/lox}$
NPY$^{cre/+}$,SIK3$^{lox/lox}$
Tail Tm (°C)
30
25
20
15
F
SIK3$^{lox/lox}$
NPY$^{cre/+}$,SIK3$^{lox/lox}$
Lumbar Tm (°C)
35
30
25
20
15
G
SIK3$^{lox/lox}$
NPY$^{cre/+}$,SIK3$^{lox/lox}$
Ppargc1a expression (Relative to control)
5
4
3
2
1
0
H
SIK3$^{lox/lox}$
NPY$^{cre/+}$,SIK3$^{lox/lox}$
Pparg expression (Relative to control)
10
8
6
4
2
0
I
SIK3$^{lox/lox}$
NPY$^{cre/+}$,SIK3$^{lox/lox}$
Prdm16 expression (Relative to control)
10
8
6
4
2
0

Supplemental Figure 4. Dermal thermography and mitochondrial biogenesis markers in HFD-fed NPY neuron SIK3 deficient mice

(A) Energy expenditure normalised to lean mass from HFD-fed $SIK3^{lox/lox}$ and $NPY^{cre/+}$,$SIK3^{lox/lox}$ mice. (B) Daily energy intake. n = 10 biologically independent samples per group. Data are mean ± s.e.m. *$P < 0.05$ for (A), by two-tailed Student's t-test. (C) Fasting-induced energy intake. n = 6 biologically independent samples per group. Data are mean ± s.e.m. **$P = 0.005$, by two-tailed Student's t-test. (D) Representative infrared thermal image of the shaved interscapular BAT, lumbar back, and tail regions of HFD-fed $SIK3^{lox/lox}$ and $NPY^{cre/+}$,$SIK3^{lox/lox}$ mice. (E) Absolute tail dermal temperature (F) Absolute dermal lumbar temperature. n = 7-8 biologically independent samples per group. Data are mean ± s.e.m. $P > 0.05$, by two-tailed Student's t-test. (G) (H) (I) mRNA expression levels of mitochondrial function/biogenesis markers in iWAT of HFD-fed $SIK3^{lox/lox}$ and $NPY^{cre/+}$,$SIK3^{lox/lox}$ mice. n = 7 biologically independent samples per group. Data are mean ± s.e.m. $P > 0.05$, by two-tailed Student's t-test. Source data are provided as a Source Data file.

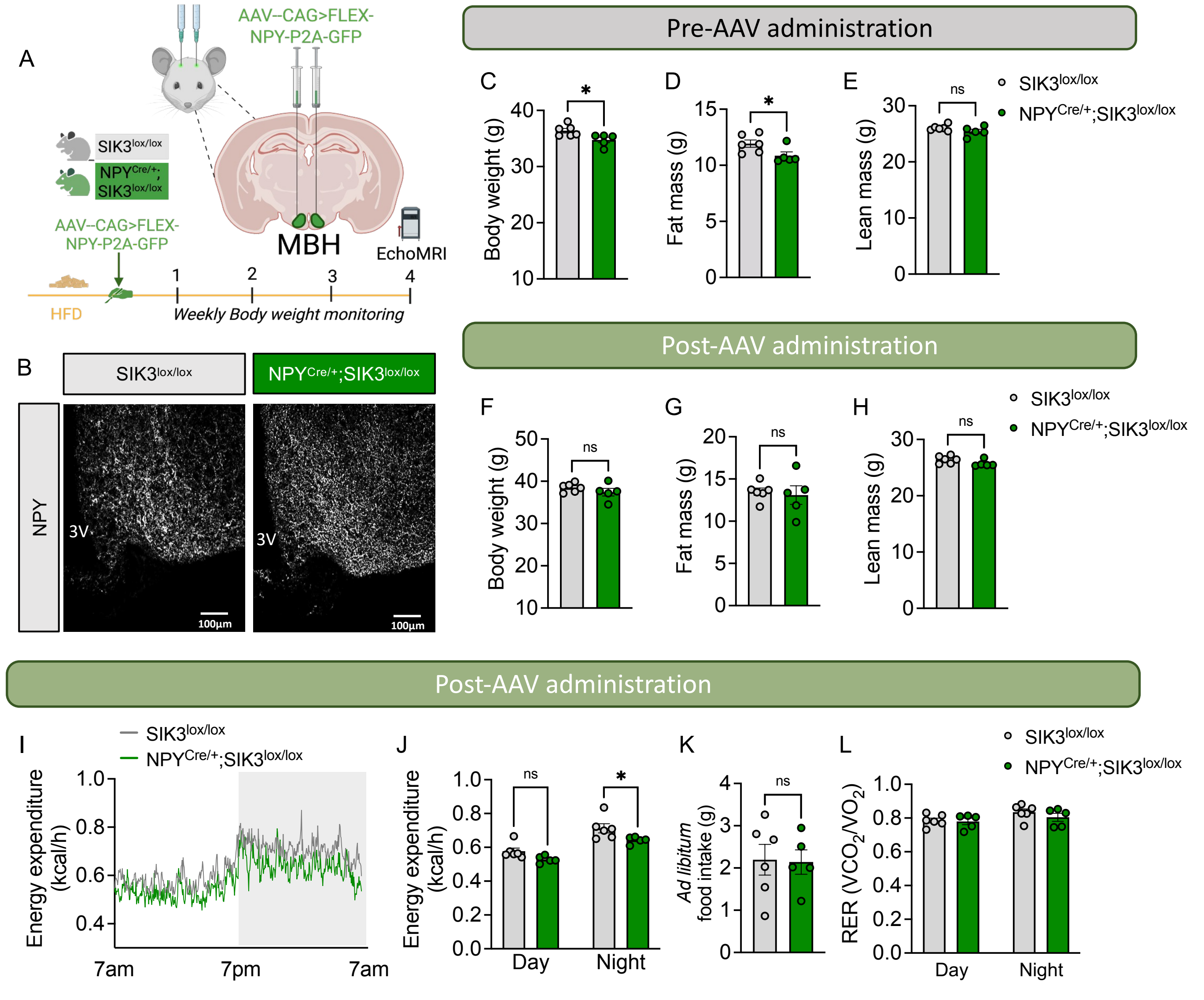

A
AAV--CAG>FLEX-NPY-P2A-GFP
SIK3lox/lox
NPYCre/+;SIK3lox/lox
AAV--CAG>FLEX-NPY-P2A-GFP
MBH
EchoMRI
HFD
1 2 3 4
Weekly Body weight monitoring
Pre-AAV administration
C D E
Body weight (g)
Fat mass (g)
Lean mass (g)
ns
SIK3lox/lox
NPYCre/+;SIK3lox/lox
B
SIK3lox/lox
NPYCre/+;SIK3lox/lox
NPY
3V
100µm
Post-AAV administration
F G H
Body weight (g)
Fat mass (g)
Lean mass (g)
ns
Post-AAV administration
I J K L
SIK3lox/lox
NPYCre/+;SIK3lox/lox
Energy expenditure (kcal/h)
7am 7pm 7am
Day Night
Ad libitum food intake (g)
RER (VCO2/VO2)

Supplemental Figure 5. Targeted restoration of NPY in the ARC of $NPY^{cre/+}$;$SIK3^{lox/lox}$ mice reversed the reduced body weight observed in HFD-fed mice.

(A) Schematic diagram of experimental design. Intra ARC injection diagram created in BioRender. Onda, D. A. (2026) https://BioRender.com/mv2x6id. (B) Representative images of NPY in the ARC of $SIK3^{lox/lox}$ and $NPY^{cre/+}$,$SIK3^{lox/lox}$ mice. Scale bar 100 µm. Pre-AAV administration measurements of (C) body weight, (D) fat mass and (E) lean mass in HFD-fed $SIK3^{lox/lox}$ and $NPY^{cre/+}$,$SIK3^{lox/lox}$ mice. n = 5-6 biologically independent samples per group. Data are mean ± s.e.m. *P = 0.026; *P = 0.048, by two-tailed Student's t-test. Four weeks post-AAV administration measurements of (F) body weight, (G) fat mass and (H) lean mass. n = 5-6 biologically independent samples per group. Data are mean ± s.e.m. $P > 0.05$, by two-tailed Student's t-test. (I)(J) Energy expenditure, (K) 24 hours *ad libitum* food intake and (L) respiratory exchange ratio of HFD-fed $SIK3^{lox/lox}$ and $NPY^{cre/+}$,$SIK3^{lox/lox}$ mice. n = 5-6 biologically independent samples per group. Data are mean ± s.e.m. *P = 0.018, by two-way ANOVA with Sidak's multiple comparisons. Source data are provided as a Source Data file.

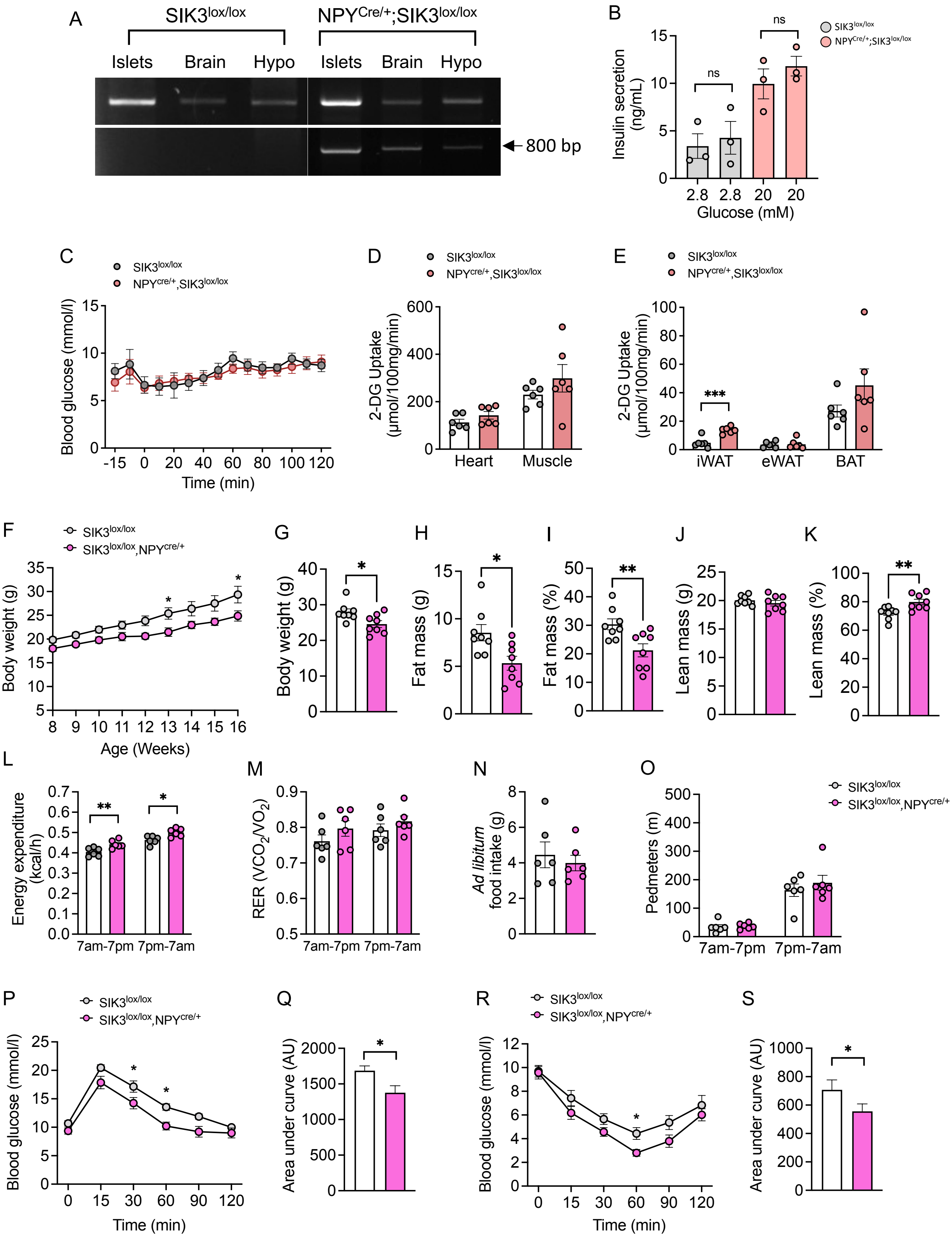
A
SIK3lox/lox
NPYCre/+;SIK3lox/lox
Islets Brain Hypo Islets Brain Hypo
800 bp
B
Insulin secretion (ng/mL)
Glucose (mM)
ns
SIK3lox/lox
NPYCre/+;SIK3lox/lox
C
Blood glucose (mmol/l)
Time (min)
NPYcre/+,SIK3lox/lox
D
2-DG Uptake (µmol/100mg/min)
Heart
Muscle
E
iWAT
eWAT
BAT
F
Body weight (g)
Age (Weeks)
SIK3lox/lox,NPYcre/+
G
Body weight (g)
H
Fat mass (g)
I
Fat mass (%)
J
Lean mass (g)
K
Lean mass (%)
L
Energy expenditure (kcal/h)
7am-7pm 7pm-7am
M
RER (VCO2/VO2)
N
Ad libitum food intake (g)
O
Pedmeters (m)
P
Blood glucose (mmol/l)
Time (min)
Q
Area under curve (AU)
R
Blood glucose (mmol/l)
Time (min)
S
Area under curve (AU)

Supplemental Figure 6. Assessment of blood glucose, peripheral glucose uptake, DNA recombination, and islet insulin secretion in NPY neurons SIK3 knockout mice

(A) Recombination of the floxed SIK3 allele (~800 bp) with $NPY^{Cre/+}$ in the pancreatic islets, brain, and hypothalamus assessed by PCR. (B) Insulin secretion under basal (2.8 mM) and high (20 mM) glucose stimulation from islets derived from $SIK3^{lox/lox}$ and $NPY^{Cre/+}$;$SIK3^{lox/lox}$ mice. n = 3 biologically independent samples per group. Data are mean ± s.e.m. *P* > 0.05, by two-way ANOVA with Sidak's multiple comparisons. (C) Blood glucose of HFD-fed $SIK3^{lox/lox}$ and $NPY^{cre/+}$,$SIK3^{lox/lox}$ mice. (D) 2-DG glucose uptake into muscle and heart. (E) 2-DG glucose uptake into iWAT, eWAT and BAT fat pads. n = 6 biologically independent samples per group. Data are mean ± s.e.m. ****P* < 0.001 for (E), by two-tailed Student's t-test. (F) Weekly body weight monitoring of HFD-fed $SIK3^{lox/lox}$ and $NPY^{Cre/+}$;$SIK3^{lox/lox}$ female mice. n = 8-9 biologically independent samples per group. Data are mean ± s.e.m. **P* < 0.05, by two-way ANOVA with Sidak's multiple comparisons. (G) Body weight post 8 weeks HFD-feeding. (H) Whole body fat mass. (I) Fat mass % normalized to body weight. (J) Whole body lean mass. (K) Lean mass % normalized to body weight. n = 8 biologically independent samples per group. Data are mean ± s.e.m. **P* = 0.022 for (G); **P* = 0.012 for (H); ***P* = 0.008 for (I); ***P* = 0.007, by two-tailed Student's t-test. (L) Energy expenditure. (M) Respiratory exchange ratio. (N) *Ad libitum* food intake. (O) Pedmeters. n = 6 biologically independent samples per group. Data are mean ± s.e.m. **P* = 0.0104 for (L); ***P* = 0.008 for (L), by two-tailed Student's t-test. (P)(Q) Intraperitoneal glucose tolerance test on 6 hour fasted mice [1 g/kg body weight]. (R)(S) Intraperitoneal Insulin tolerance test on 4 hour fasted mice [0.75 IU/kg body weight]. n = 8-9 biologically independent samples per group. Data are mean ± s.e.m. **P* < 0.05, by two-tailed Student's t-test or two-way ANOVA with Sidak's multiple comparisons. Source data are provided as a Source Data file.

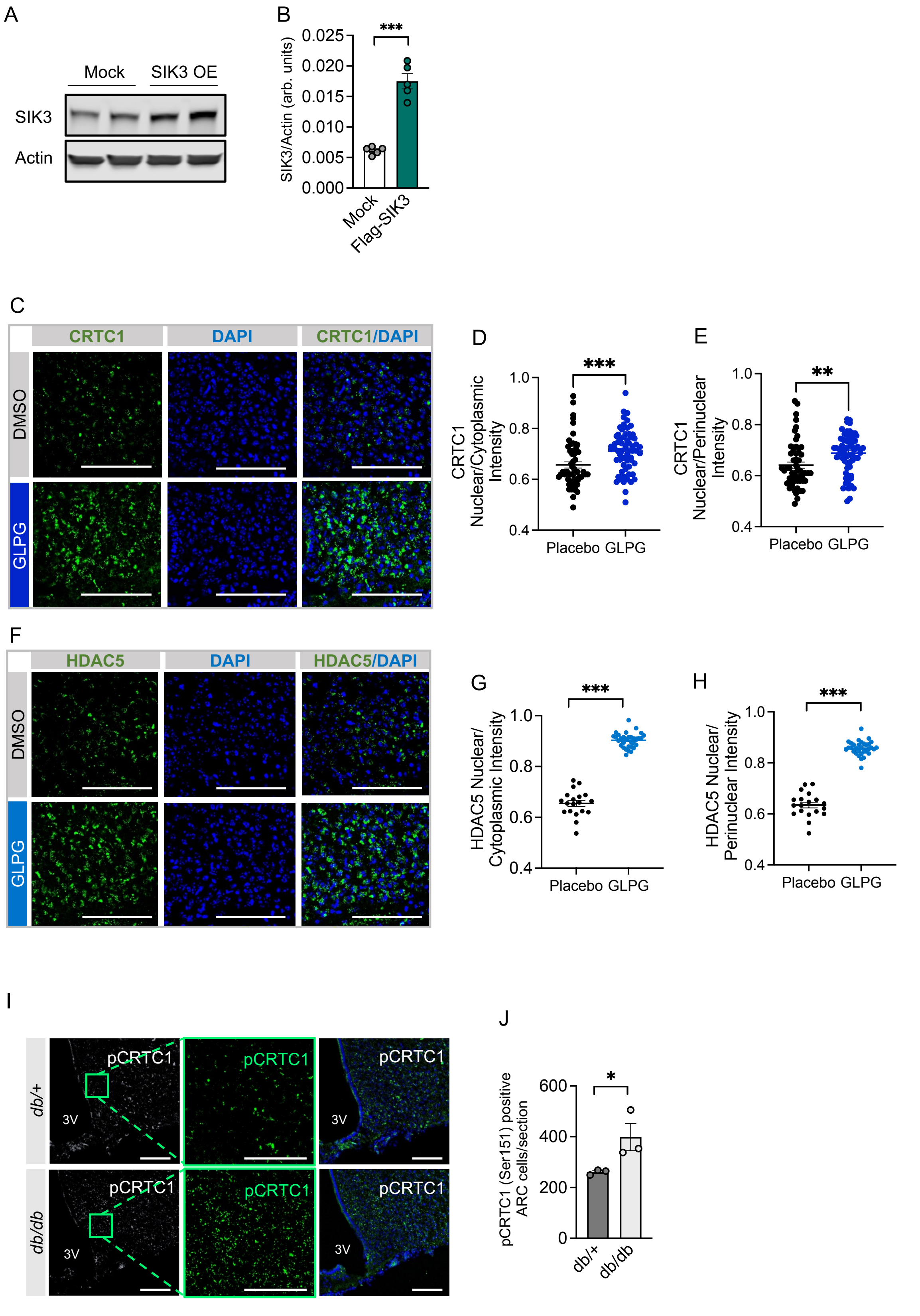
A
Mock
SIK3 OE
SIK3
Actin
B
***
SIK3/Actin (arb. units)
0.025
0.020
0.015
0.010
0.005
0.000
Mock
Flag-SIK3
C
CRTC1
DAPI
CRTC1/DAPI
DMSO
GLPG
D
***
CRTC1
Nuclear/Cytoplasmic
Intensity
1.0
0.8
0.6
0.4
Placebo GLPG
E
**
CRTC1
Nuclear/Perinuclear
Intensity
1.0
0.8
0.6
0.4
Placebo GLPG
F
HDAC5
DAPI
HDAC5/DAPI
DMSO
GLPG
G
***
HDAC5 Nuclear/
Cytoplasmic Intensity
1.0
0.8
0.6
0.4
Placebo GLPG
H
***
HDAC5 Nuclear/
Perinuclear Intensity
1.0
0.8
0.6
0.4
Placebo GLPG
I
db/+
db/db
pCRTC1
3V
J
*
pCRTC1 (Ser151) positive
ARC cells/section
600
400
200
0
db/+
db/db

Supplemental Figure 7. Validation of SIK3 overexpression in SK-N-SH-SY5Y neuroblastoma cells and CRTC1 and HDAC5 immunostaining in placebo and GLPG3970 treated DIO mice.

(A) Immunoblot and (B) densitometry analysis of SIK3 in wild-type/Mock and SIK3 overexpressed (SIK3 OE) SK-N-SH-SY5Y neuroblastoma cells. n = 5 biologically independent samples per group. Data are mean ± s.e.m. ***$P < 0.001$, by two-tailed Student's t-test. (C) Immunostaining of CRTC1 in placebo and GLPG3970 treated HFD-fed mice. (D) CRTC1 Nuclear/Cytoplasmic ratio and (E) CRTC1 Nuclear/Perinuclear ratio. Scale bar, 100 μm. n = 57-64 per group. Data are mean ± s.e.m. **$P = 0.002$ for (E); ***$P < 0.001$ for (D), by two-tailed Student's t-test. *n* corresponds to the nuclear/perinuclear-to-cytoplasmic CRTC1 intensity ratio in one brain section. Data were derived from 5-6 biologically independent samples per group. (F) HDAC5 immunostaining in placebo and GLPG3970 treated HFD-fed mice. (G) HDAC5 Nuclear/Cytoplasmic ratio. (H) HDAC5 Nuclear/Perinuclear ratio. Scale bar, 100 μm. n = 19-32 per group. Data are mean ± s.e.m. ***$P < 0.001$, by two-tailed Student's t-test. *n* corresponds to the nuclear/perinuclear-to-cytoplasmic HDAC5 intensity ratio in one brain section. Data were derived from 5-6 biologically independent samples per group. (I) (J) Immunostaining of pCRTC1 (Ser151) in the ARC of lean (*db/+*) and obese (*db/db*) mice. Scale bar, 100 μm. n = 3 biologically independent samples per group. Data are mean ± s.e.m. *$P = 0.03$, by one-tailed Student's t-test. Source data are provided as a Source Data file.

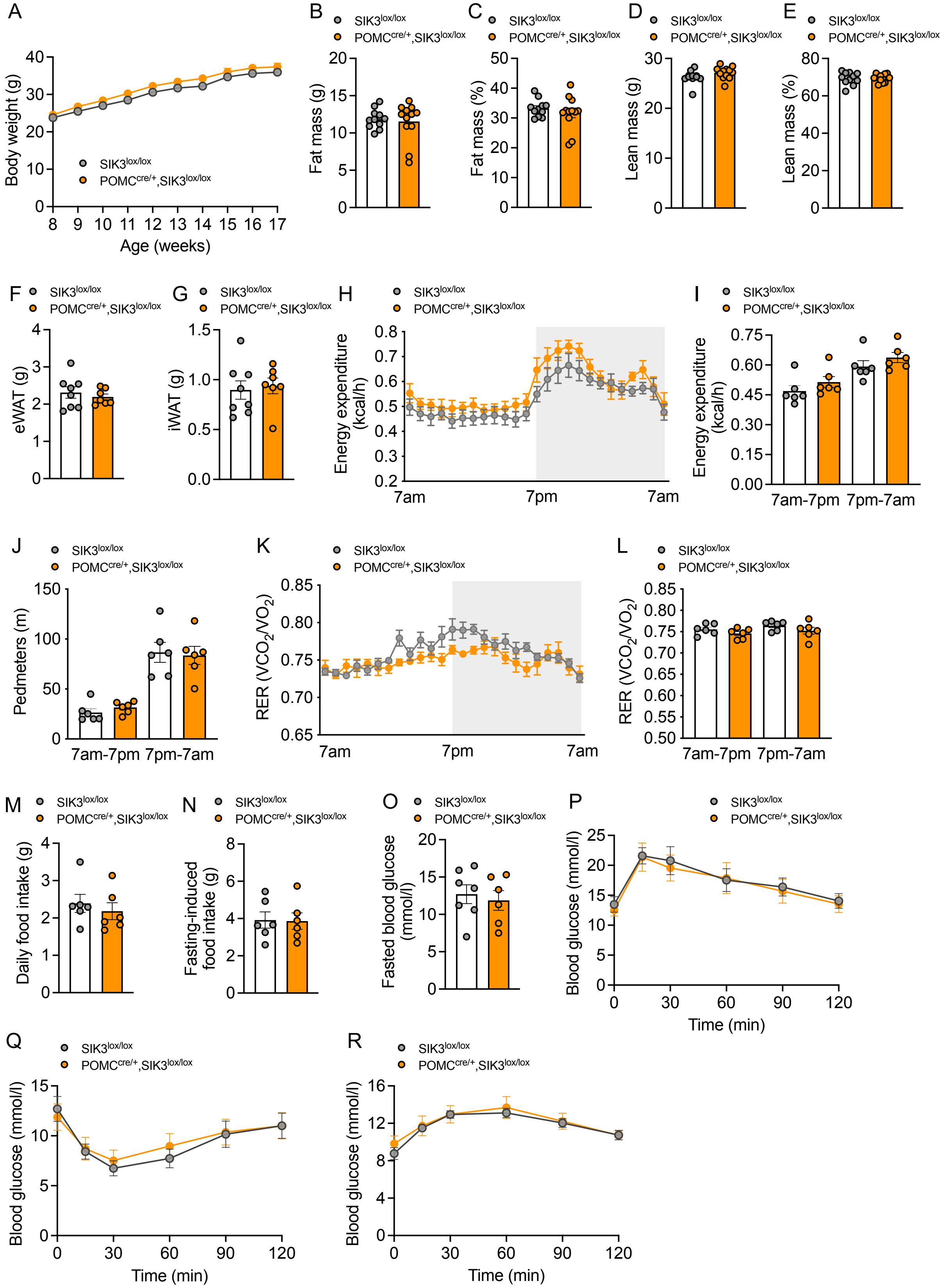

A
B
C
D
E
F
G
H
I
J
K
L
M
N
O
P
Q
R
SIK3lox/lox
POMCcre/+,SIK3lox/lox
Body weight (g)
Age (weeks)
Fat mass (g)
Fat mass (%)
Lean mass (g)
Lean mass (%)
eWAT (g)
iWAT (g)
Energy expenditure (kcal/h)
7am
7pm
7am-7pm
7pm-7am
Pedmeters (m)
RER (VCO2/VO2)
Daily food intake (g)
Fasting-induced food intake (g)
Fasted blood glucose (mmol/l)
Blood glucose (mmol/l)
Time (min)

Supplemental Figure 8. Ablation of SIK3 in POMC neurons does not affect energy homeostasis and glucose metabolism

(A) Weekly body weight. (B) Fat mass. (C) Fat mass % normalized to body weight. (D) Lean mass. (E) Lean mass % normalized to body weight. n = 11-12 biologically independent samples per group. Data are mean ± s.e.m. $P > 0.05$, by two-tailed Student's t-test or two-way ANOVA, with Sidak's multiple comparisons. (F) (G) Dissected weights of individual fat pads from eWAT and iWAT. n = 8-7 biologically independent samples per group. Data are mean ± s.e.m. $P > 0.05$, by two-tailed Student's t-test. (H) (I) Energy expenditure. (J) Pedmeters. (K) (L) RER. (M) Daily food intake. (N) Fasting-induced food intake. (O) 16 h fasted blood glucose. (P) Glucose tolerance, (Q) insulin tolerance, and (R) pyruvate tolerance of HFD-fed $SIK3^{lox/lox}$ and $POMC^{cre/+}$,$SIK3^{lox/lox}$ mice. n = 6 biologically independent samples per group. Data are mean ± s.e.m. $P > 0.05$, by two-tailed Student's t-test or two-way ANOVA with Sidak's multiple comparisons. Source data are provided as a Source Data file.

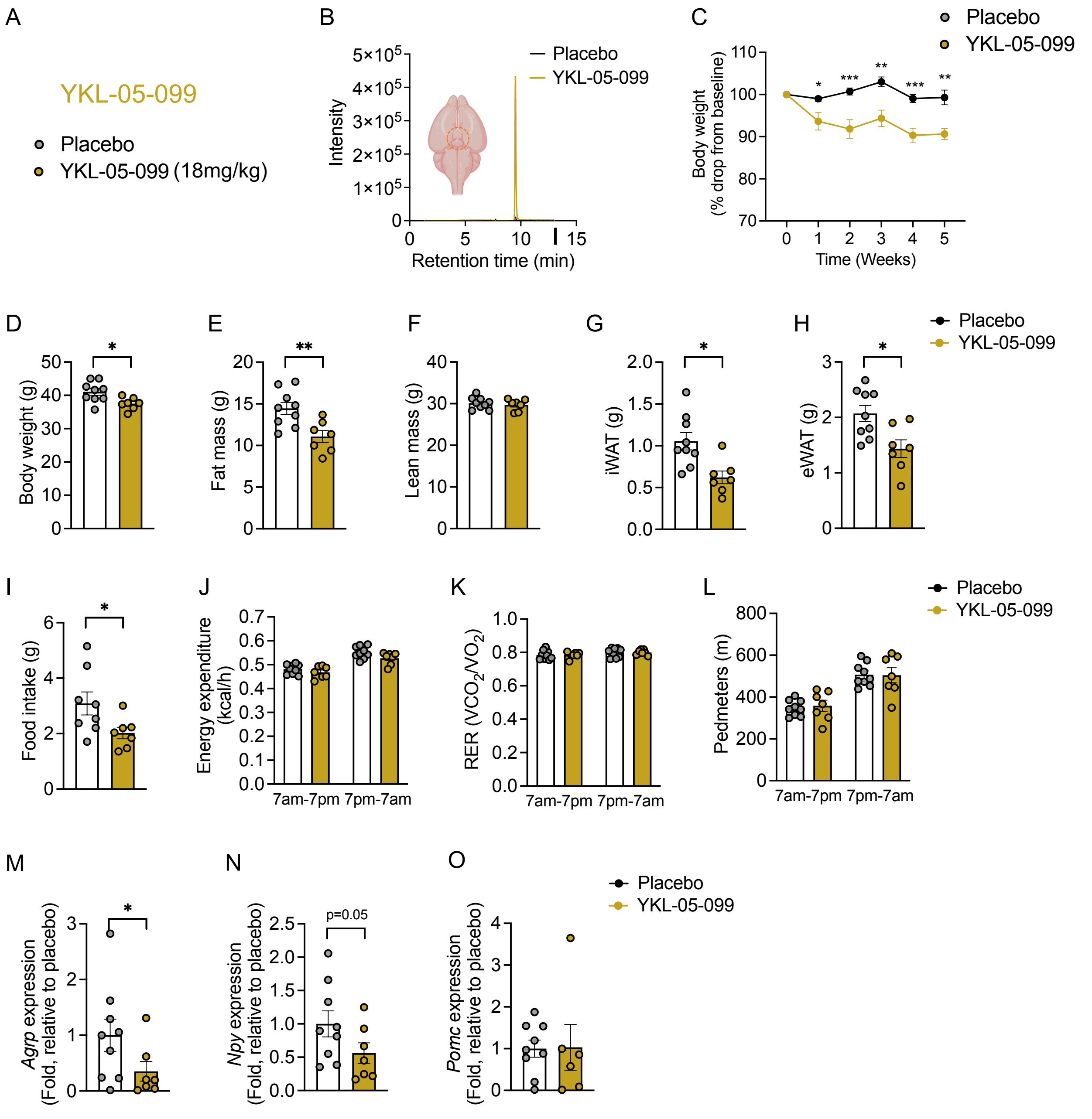
A
YKL-05-099
Placebo
YKL-05-099 (18mg/kg)
B
Intensity
Retention time (min)
Placebo
YKL-05-099
C
Placebo
YKL-05-099
Body weight (% drop from baseline)
Time (Weeks)
D
Body weight (g)
E
Fat mass (g)
F
Lean mass (g)
G
iWAT (g)
H
eWAT (g)
Placebo
YKL-05-099
I
Food intake (g)
J
Energy expenditure (kcal/h)
7am-7pm 7pm-7am
K
RER (VCO2/VO2)
7am-7pm 7pm-7am
L
Pedmeters (m)
7am-7pm 7pm-7am
Placebo
YKL-05-099
M
Agrp expression (Fold, relative to placebo)
N
Npy expression (Fold, relative to placebo)
p=0.05
O
Pomc expression (Fold, relative to placebo)
Placebo
YKL-05-099

Supplemental Figure 9. The pan-SIK inhibitor YKL-05-099 promotes weight loss and suppresses food intake in HFD-fed obese mice.

(A) Schematic illustrating two experimental mouse groups: Placebo and YKL-05-099 (18 mg/kg, oral, once daily for 5 weeks), with color-coded symbols indicating the respective treatments. (B) Qualitative chromatograms showing detection of YKL-05-099 in mouse hypothalamus. Brain diagram created in BioRender. Onda, D. A. (2026) https://BioRender.com/mv2x6id. (C) Body weight % drop from baseline. n = 7-9 biologically independent samples per group. Data are mean ± s.e.m. $^*P < 0.05$; $^{**}P < 0.01$; $^{***}P < 0.001$, by two-way ANOVA with Sidak's multiple comparisons. (D) Body weight. (E) Fat mass. (F) Lean mass. Dissected weights of individual fat depots from (G) iWAT (H) eWAT. n = 7-9 biologically independent samples per group. Data are mean ± s.e.m. $^*P < 0.05$; $^{**}P < 0.01$; by two-tailed Student's t-test. (I) Daily food intake. (J) Energy expenditure. (K) Respiratory exchange ratio. (L) Pedmeters. n = 7-9 biologically independent samples per group. Data are mean ± s.e.m. $^*P = 0.046$ for (I), by two-tailed Student's t-test. Whole hypothalamic (M) *Agrp*, (N) *Npy,* (O) *Pomc* gene expression in HFD-fed C57BL/6 mice treated with placebo or YKL-05-099. n = 7-9 biologically independent samples per group. Data are mean ± s.e.m. $^*P = 0.048$ for (M), by one-tailed Student's t-test. Source data are provided as a Source Data file.

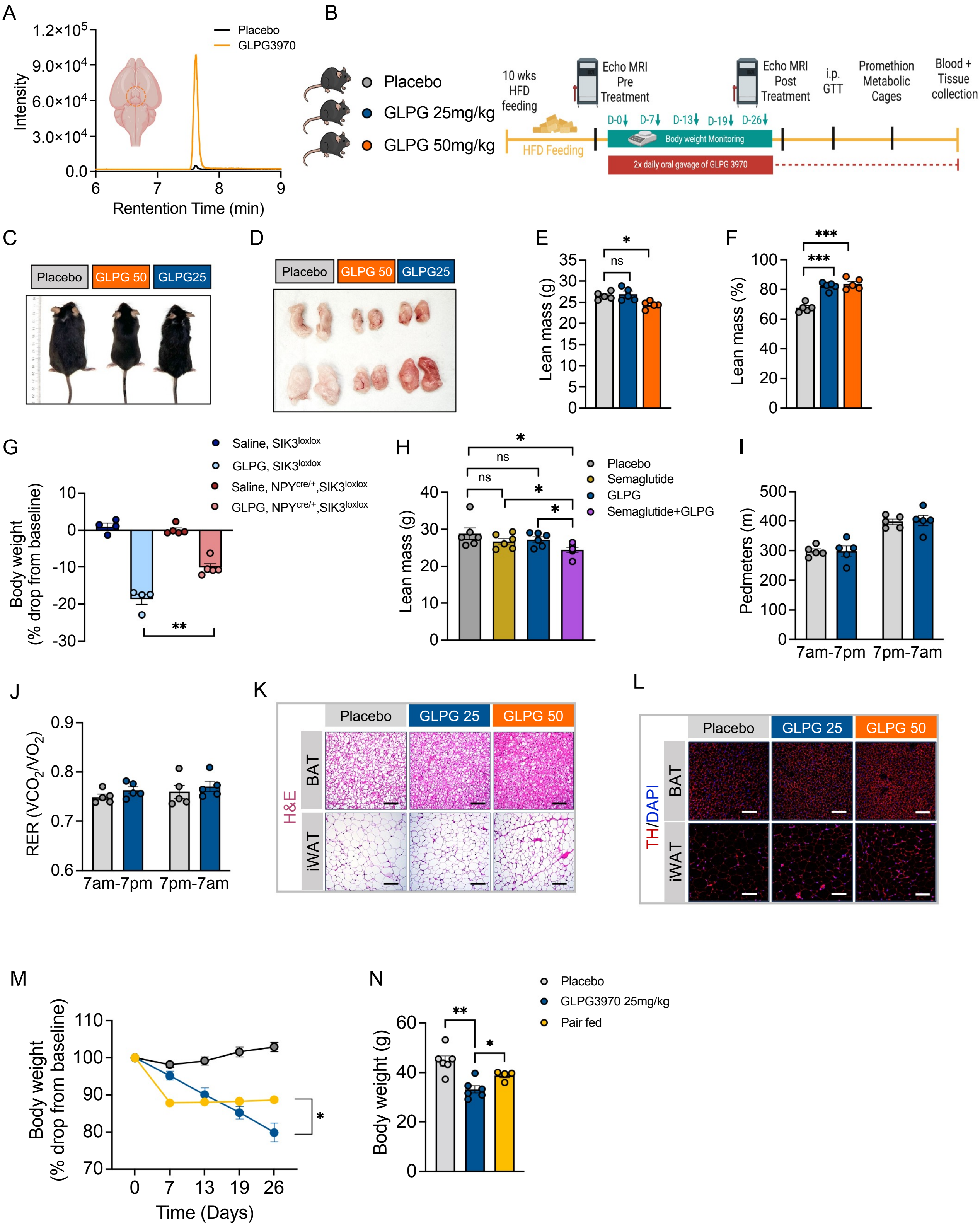

A
Intensity
Rentention Time (min)
Placebo
GLPG3970
B
Placebo
GLPG 25mg/kg
GLPG 50mg/kg
10 wks HFD feeding
Echo MRI Pre Treatment
Echo MRI Post Treatment
i.p. GTT
Promethion Metabolic Cages
Blood + Tissue collection
HFD Feeding
D-0 D-7 D-13 D-19 D-26
Body weight Monitoring
2x daily oral gavage of GLPG 3970
C
Placebo GLPG 50 GLPG25
D
Placebo GLPG 50 GLPG25
E
Lean mass (g)
F
Lean mass (%)
G
Saline, SIK3loxlox
GLPG, SIK3loxlox
Saline, NPYcre/+,SIK3loxlox
GLPG, NPYcre/+,SIK3loxlox
Body weight (% drop from baseline)
H
Placebo
Semaglutide
GLPG
Semaglutide+GLPG
Lean mass (g)
I
Pedmeters (m)
7am-7pm 7pm-7am
J
RER (VCO2/VO2)
7am-7pm 7pm-7am
K
Placebo GLPG 25 GLPG 50
H&E
BAT
iWAT
L
Placebo GLPG 25 GLPG 50
TH/DAPI
BAT
iWAT
M
Body weight (% drop from baseline)
Time (Days)
N
Placebo
GLPG3970 25mg/kg
Pair fed
Body weight (g)

Supplemental Figure 10. Pharmacological inhibition of SIK3 by GLPG3970 promotes weight loss and improves metabolic health outcomes

(A) Qualitative chromatograms showing detection of GLPG3970 in mouse hypothalamus. Brain diagram created in BioRender. Onda, D. A. (2026) https://BioRender.com/mv2x6id. (B) Experimental design: WT C57BL/6 mice were fed with HFD for 10 weeks until they reach ~40g. Mice were then treated with Placebo or 25 mg/kg or 50 mg/kg GLPG3970 orally twice daily for 26 days. Treatment regimen and experimental design diagram created in BioRender. Onda, D. A. (2026) https://BioRender.com/mv2x6id. (C) Representative image of HFD-fed mice after 26 days of GLPG3970 treatment. (D) Representative image of individual fat pads from eWAT and iWAT. (E) Lean mass (F) Lean mass % normalized to body weight of HFD-fed WT mice post GLPG3970 treatment. n = 5 biologically independent samples per group. Data are mean ± s.e.m. *$P = 0.045$, by two-tailed Student's t-test. (G) Body weight expressed as % drop from baseline of HFD-fed $SIK3^{lox/lox}$ and $NPY^{cre/+}$,$SIK3^{lox/lox}$ mice treated with Placebo or 25 mg/kg GLPG3970 orally twice daily for 26 days. n = 4-5 biologically independent samples per group. Data are mean ± s.e.m. **$P = 0.0014$, by two-tailed Student's t-test. (H) Lean mass of HFD-fed mice treated with 25 mg/kg GLPG3970 twice every 24 hours alone or in combination with 10 nmol/kg Semaglutide once every 48 hours. n = 6 biologically independent samples per group. Data are mean ± s.e.m. *$P < 0.05$, by two-tailed Student's t-test. (I) Pedmeters (J) RER n = 5 biologically independent samples per group. Data are mean ± s.e.m. $P > 0.05$, two-tailed Student's t-test. (K) H&E staining of iWAT and BAT. Scale bar, 100 μm. (L) Immunostaining of TH in iWAT and BAT. Scale bar, 100 μm. Placebo-treated mice were pair-fed (PF) to the food intake of GLPG3970-treated mice. (M) (N) Body weight expressed as % drop from baseline. n = 4-6 biologically independent samples per group. Data are mean ± s.e.m. *$P = 0.025$; **$P = 0.0016$ for (N), by two-tailed Student's t-test. *$P < 0.05$, by two-way ANOVA with Tukey's multiple comparisons for (M). Source data are provided as a Source Data file.

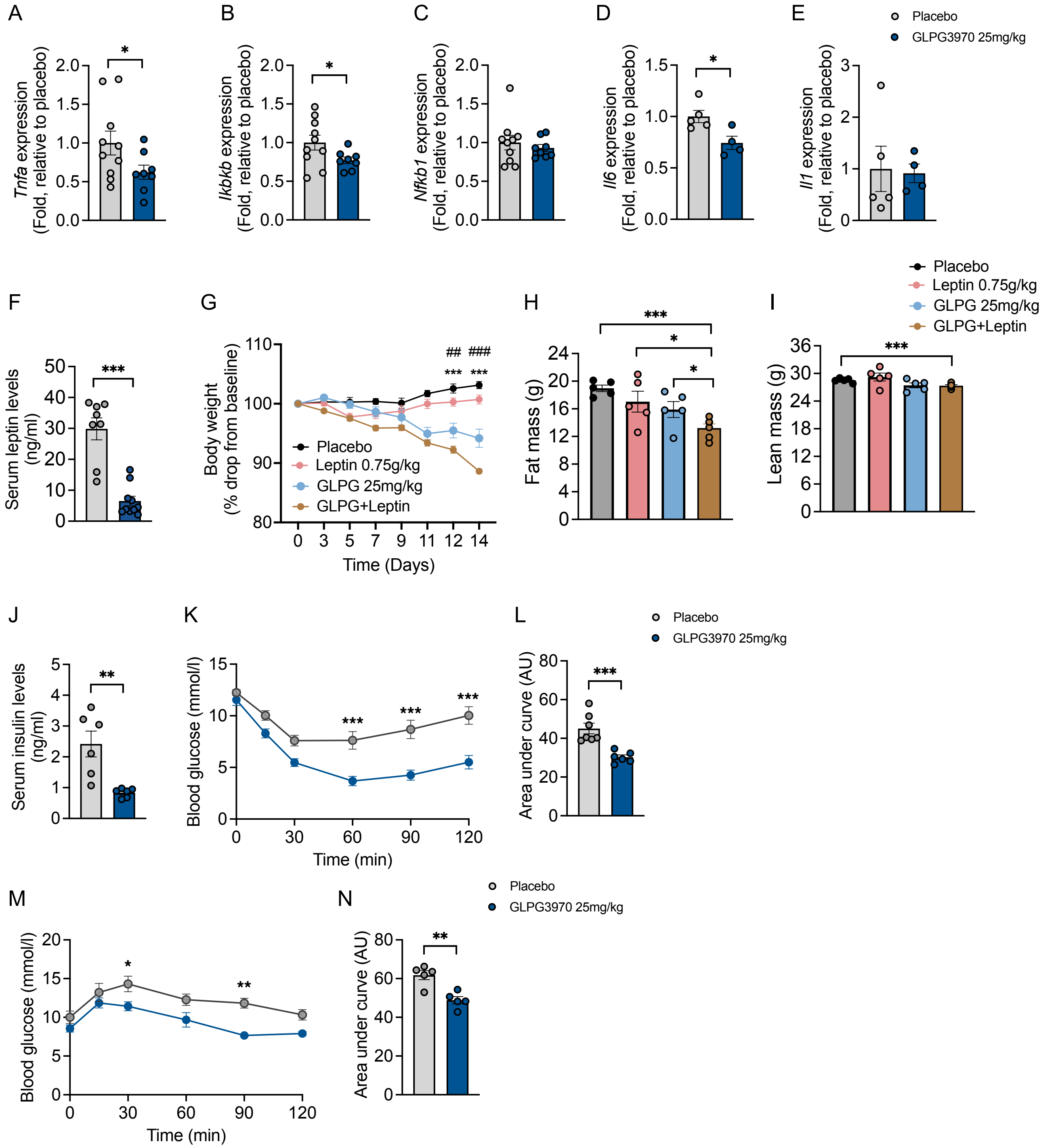

A
B
C
D
E
Placebo
GLPG3970 25mg/kg
Tnfa expression
(Fold, relative to placebo)
Ikbkb expression
(Fold, relative to placebo)
Nfkb1 expression
(Fold, relative to placebo)
Il6 expression
(Fold, relative to placebo)
Il1 expression
(Fold, relative to placebo)
F
G
H
I
Placebo
Leptin 0.75g/kg
GLPG 25mg/kg
GLPG+Leptin
Serum leptin levels
(ng/ml)
Body weight
(% drop from baseline)
Time (Days)
###
Fat mass (g)
Lean mass (g)
J
K
L
Serum insulin levels
(ng/ml)
Blood glucose (mmol/l)
Time (min)
Area under curve (AU)
M
N
Blood glucose (mmol/l)
Time (min)
Area under curve (AU)

Supplemental Figure 11. GLPG3970 treatment suppresses hypothalamic inflammatory markers and leptin co-treatment enhances its weight-loss effect.

Hypothalamic (A) *Tnfα*, (B) *Ikbkb,* (C) *Nfkb1* mRNA expression in placebo and GLPG3970-treated HFD-fed mice. n = 8-10 biologically independent samples per group. Data are mean ± s.e.m. *P = 0.033 for (A); *P = 0.031 for (B), by one-tailed Student's t-test. (D) *Il6, and (E) Il1* mRNA expression in placebo and GLPG3970-treated HFD-fed mice. n = 4-5 biologically independent samples per group. Data are mean ± s.e.m. *P = 0.011 for (D), by one-tailed Student's t-test. (F) Serum leptin in placebo and GLPG3970-treated HFD-fed mice. n = 8-10 biologically independent samples per group. Data are mean ± s.e.m. ***P < 0.001, by two-tailed Student's t-test. WT C57/BL6 mice were fed with HFD for 10 weeks. Mice were then treated with placebo, GLPG3970 (25mg/kg), leptin (1 μg/g, i.p. twice daily), or combination of both GLPG3970 and leptin for 14 days. (G) Body weight % drop from baseline. n = 5-6 biologically independent samples per group. ***P < 0.001; $^{\#\#}P$ < 0.01; $^{\#\#\#}P$ < 0.001, by two-way ANOVA with Tukey's multiple comparisons. (*Leptin vs GLPG+Leptin; $^{\#}$GLPG vs GLPG+Leptin). (H) Fat mass and (I) lean mass post 14 days of treatment. n = 5-6 biologically independent samples per group. *P < 0.05; ***P < 0.001, by two-tailed Student's t-test. (J) Serum insulin. n = 6 biologically independent samples per group. Data are mean ± s.e.m. **P = 0.0038, by two-tailed Student's t-test. (K) (L) Intraperitoneal insulin tolerance test on 4 hour fasted mice [0.75 IU/kg body weight]. (M) (N) Intraperitoneal pyruvate tolerance test on 16 hour fasted mice [1 g/kg body weight]. n = 5-7 biologically independent samples per group. Data are mean ± s.e.m. *P < 0.05; **P < 0.01; ***P < 0.001, by two-tailed Student's t-test for (L and N) or two-way ANOVA with Sidak's multiple comparisons for (K and M). Source data are provided as a Source Data file.

Uncropped and Unprocessed Western Blots

Figure 3E

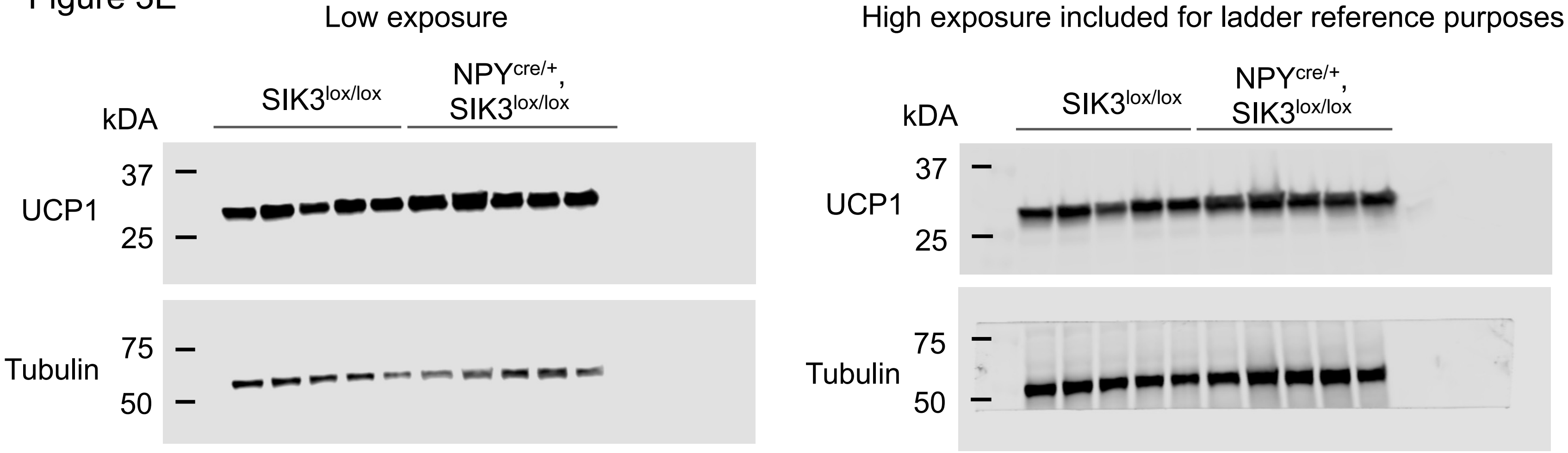

Figure 3H

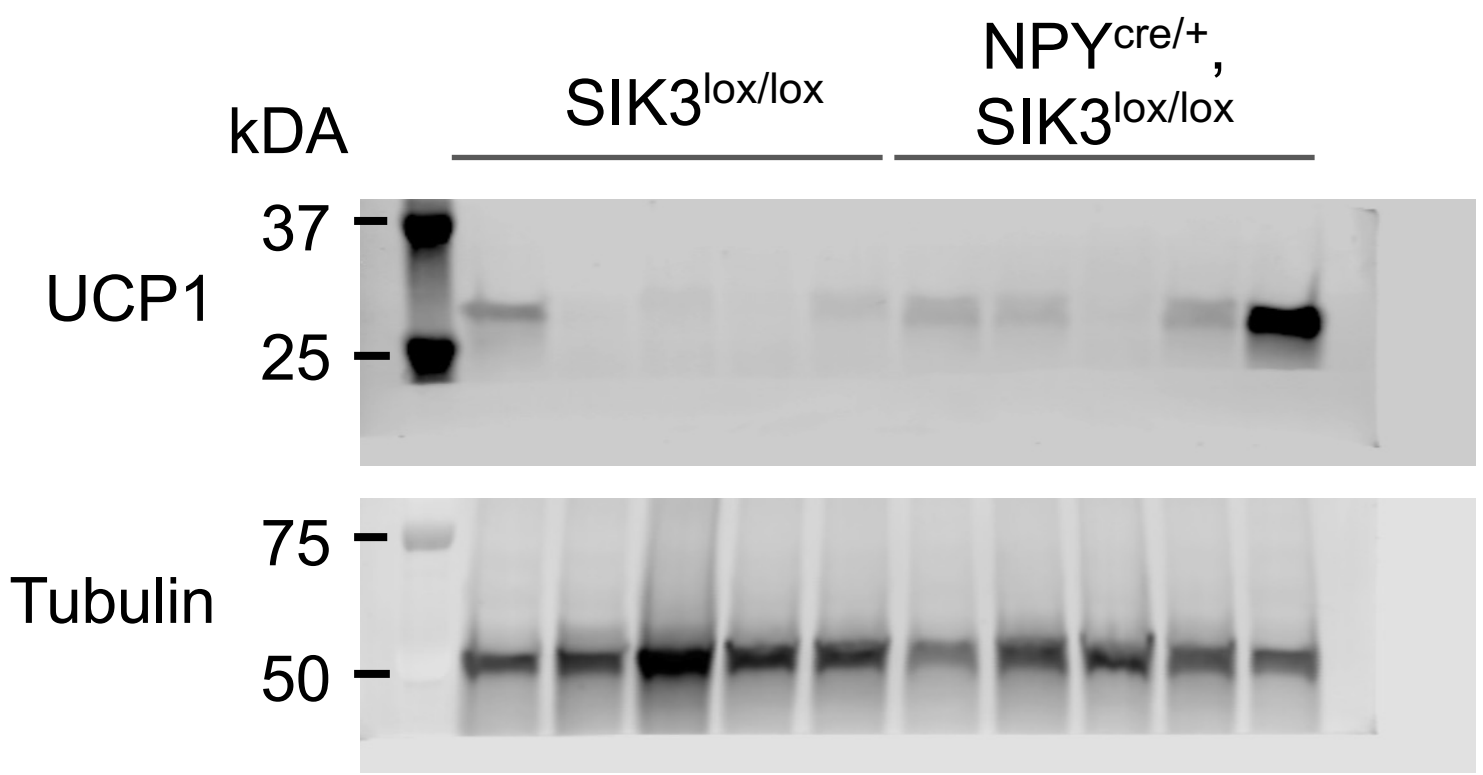

Figure 4K

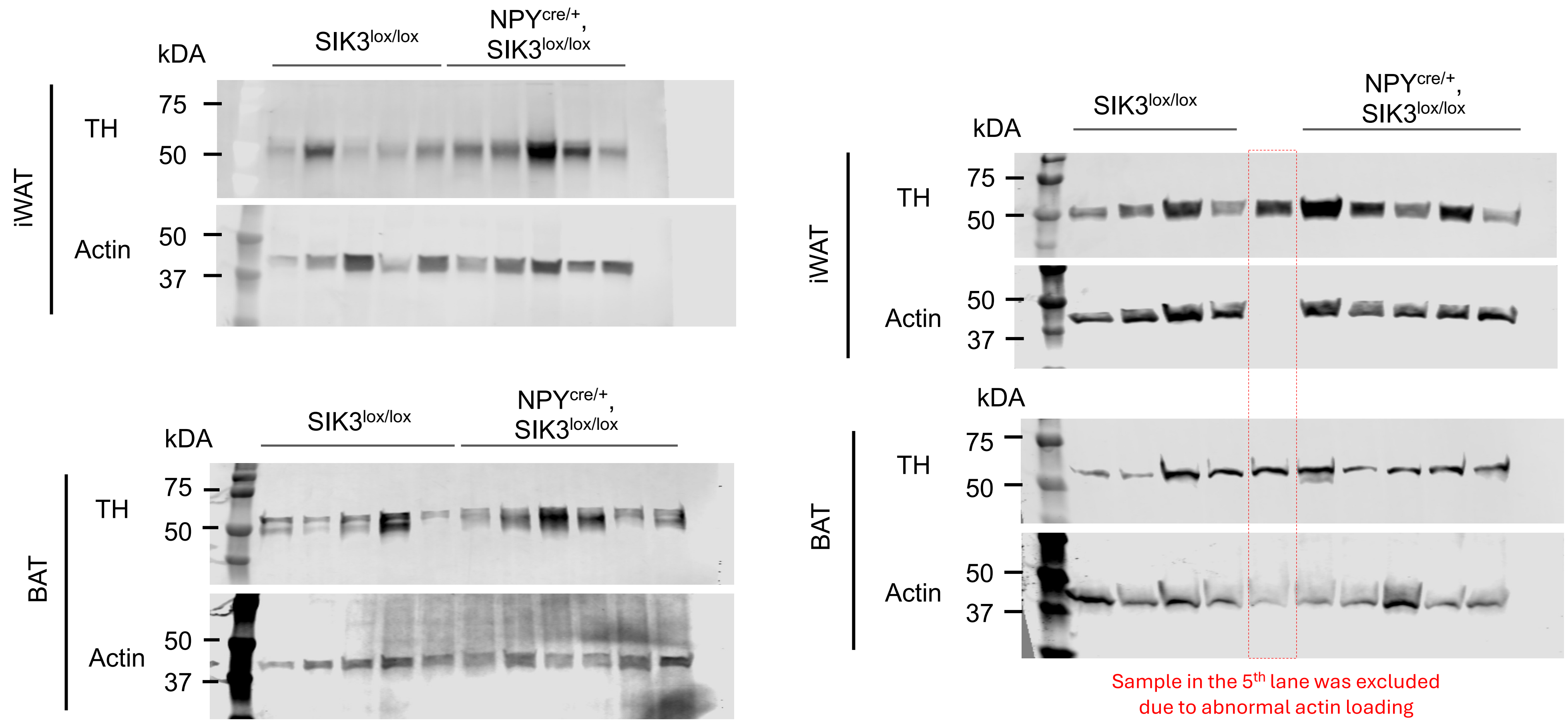

kDA
$SIK3^{lox/lox}$
$NPY^{cre/+}$, $SIK3^{lox/lox}$
iWAT
TH
Actin
75
50
50
37
BAT
TH
Actin
75
50
50
37
Sample in the 5th lane was excluded due to abnormal actin loading

Figure 7F

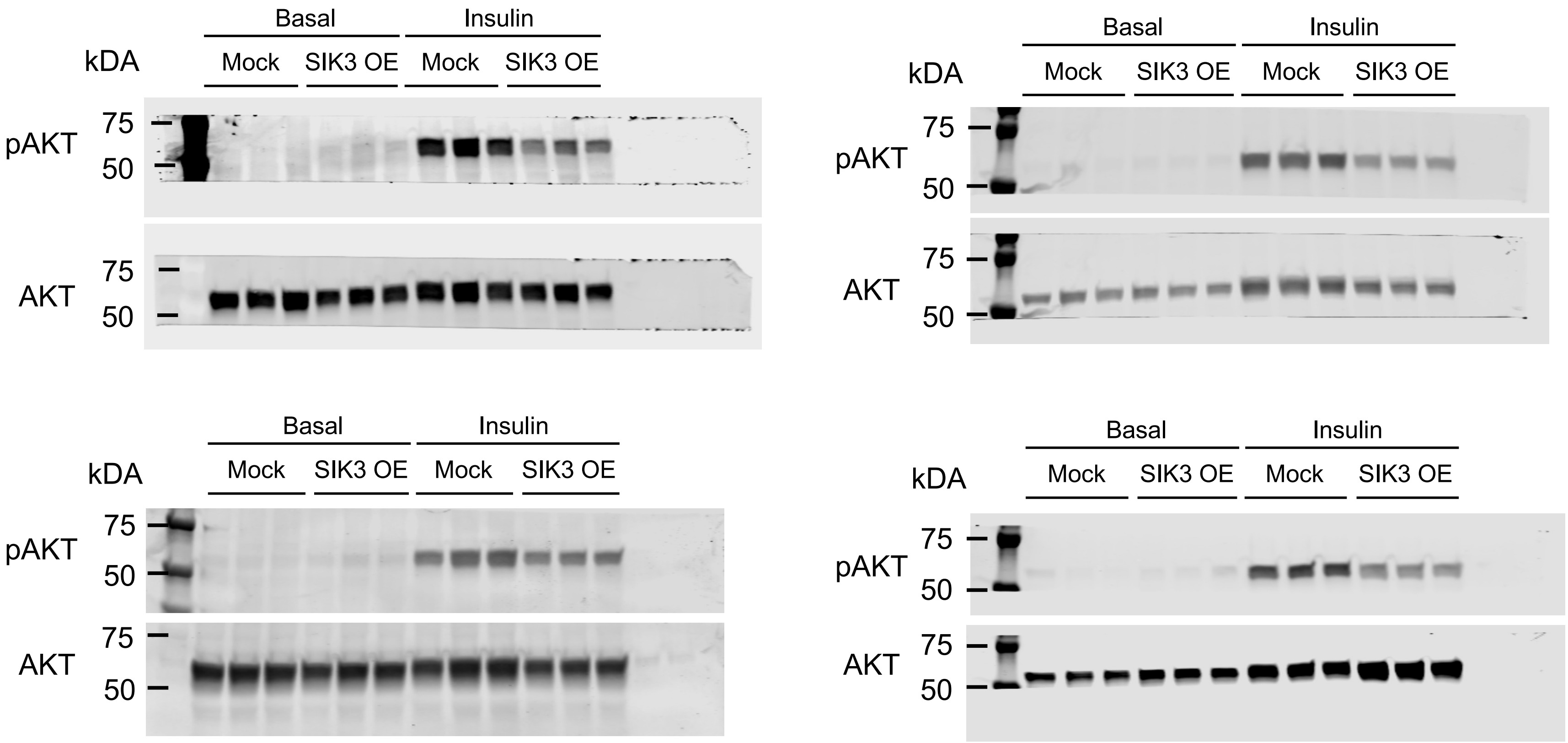

Figure 7H

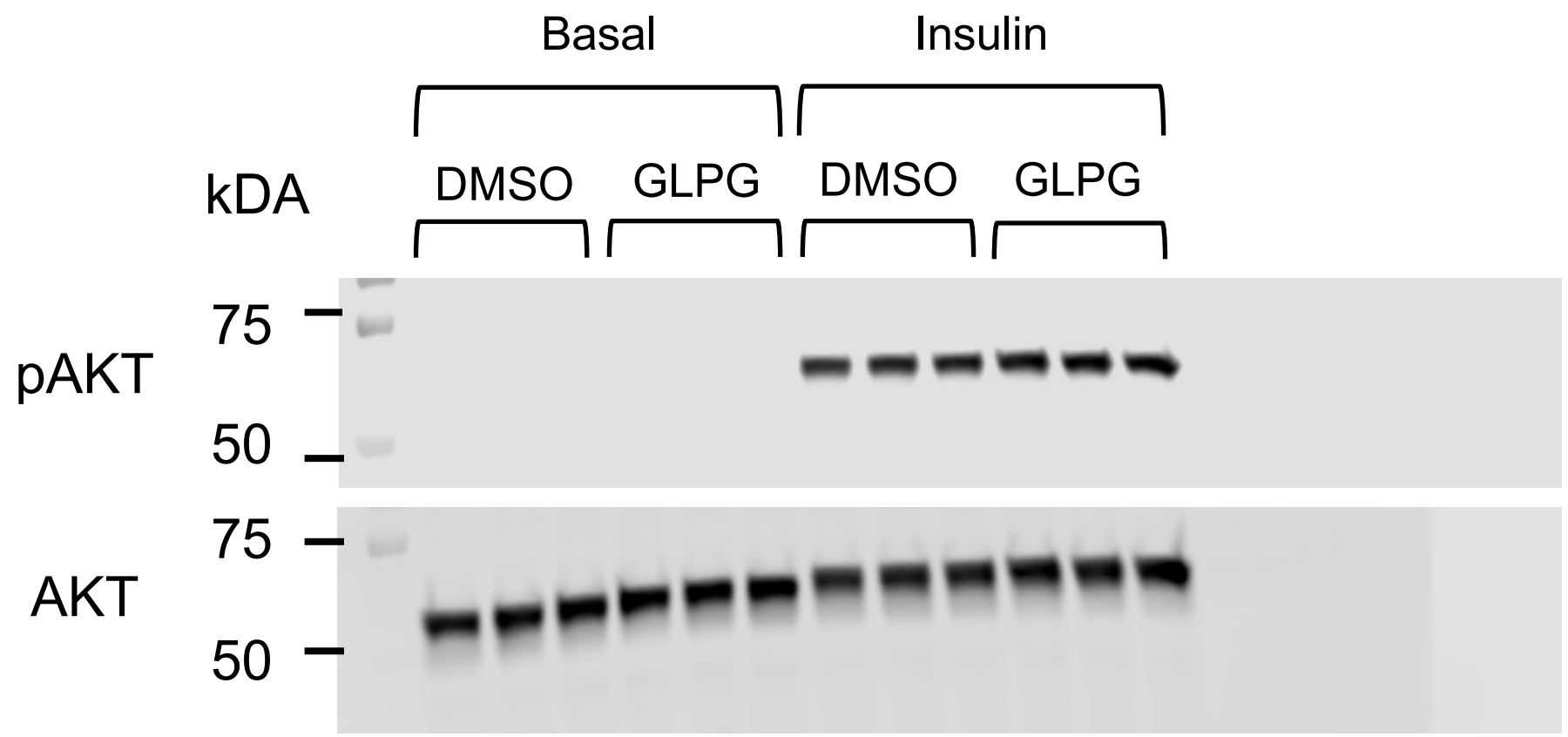

Basal
Insulin
kDA
DMSO
GLPG
DMSO
GLPG
75
pAKT
50
75
AKT
50

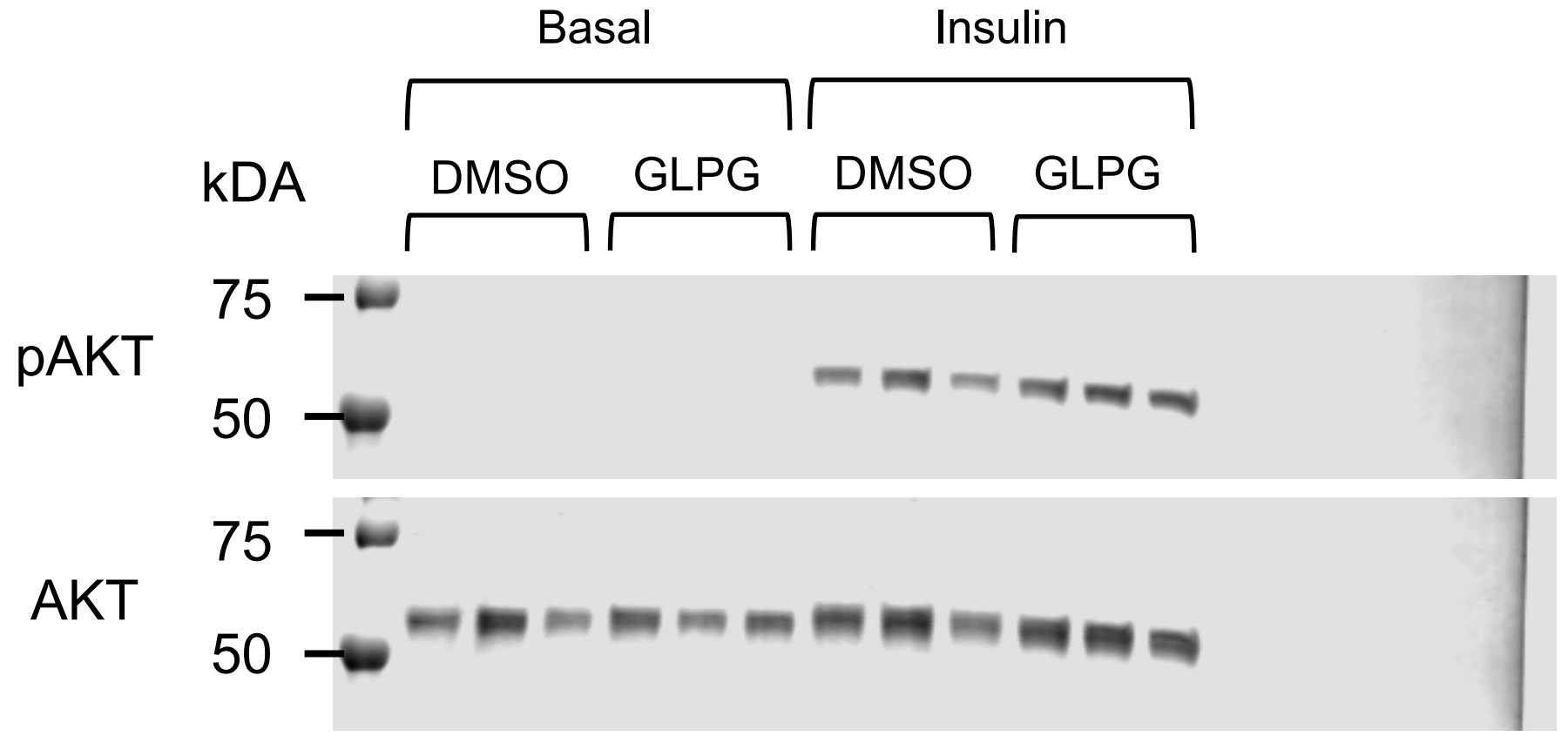

Basal
Insulin
kDA
DMSO
GLPG
DMSO
GLPG
75
pAKT
50
75
AKT
50

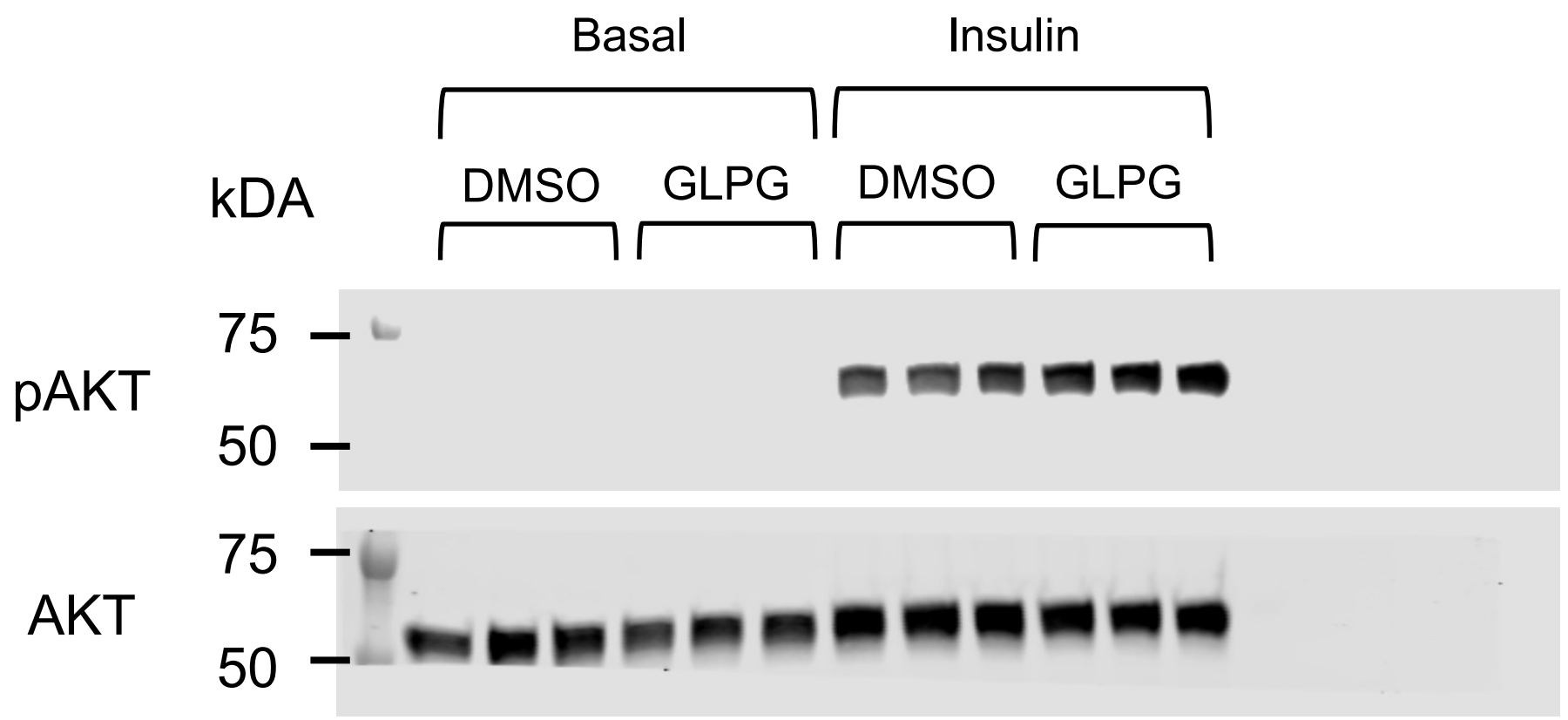

Basal
Insulin
kDA
DMSO
GLPG
DMSO
GLPG
75
pAKT
50
75
AKT
50

Figure 7J

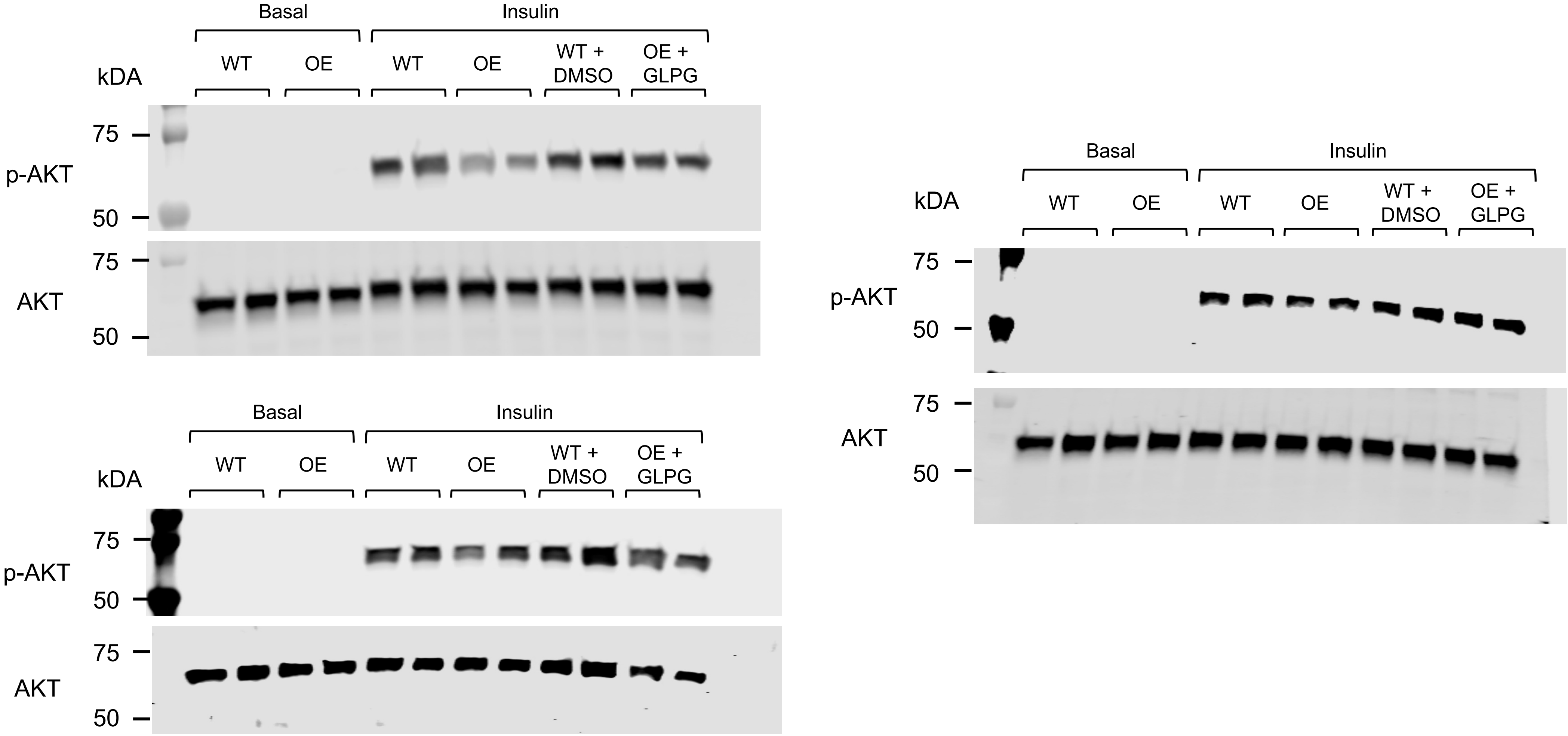

Figure S1K

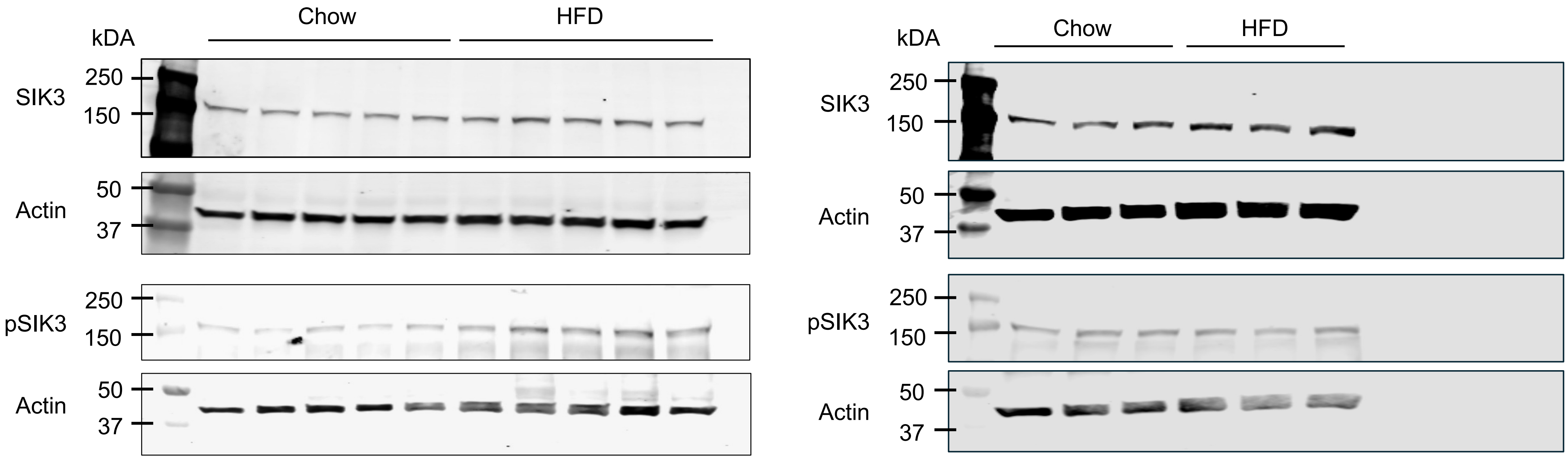

Chow
HFD
kDA
SIK3
250
150
Actin
50
37
pSIK3
250
150
Actin
50
37
Chow
HFD
kDA
SIK3
250
150
Actin
50
37
pSIK3
250
150
Actin
50
37
