# Supplementary material for: Targeting hypothalamic SIK3 to promote weight loss and improve glycemic control in mice
Source: Nat Commun. 2026 Jun 2;17:8432. doi: 10.1038/s41467-026-73649-1 (PMC13478199; doi:10.1038/s41467-026-73649-1)
Supplement: Supplementary file 2 [file 41467_2026_73649_MOESM2_ESM.pdf]

nature portfolio

Corresponding author(s): Kim Loh

Last updated by author(s): Mar 12, 2025

Reporting Summary

Nature Portfolio wishes to improve the reproducibility of the work that we publish. This form provides structure for consistency and transparency in reporting. For further information on Nature Portfolio policies, see our Editorial Policies and the Editorial Policy Checklist.

Statistics

For all statistical analyses, confirm that the following items are present in the figure legend, table legend, main text, or Methods section.

n/a	Confirmed	
☐	☒	The exact sample size (n) for each experimental group/condition, given as a discrete number and unit of measurement
☐	☒	A statement on whether measurements were taken from distinct samples or whether the same sample was measured repeatedly
☐	☒	The statistical test(s) used AND whether they are one- or two-sided<br>*Only common tests should be described solely by name; describe more complex techniques in the Methods section.*
☒	☐	A description of all covariates tested
☐	☒	A description of any assumptions or corrections, such as tests of normality and adjustment for multiple comparisons
☐	☒	A full description of the statistical parameters including central tendency (e.g. means) or other basic estimates (e.g. regression coefficient) AND variation (e.g. standard deviation) or associated estimates of uncertainty (e.g. confidence intervals)
☐	☒	For null hypothesis testing, the test statistic (e.g. F, t, r) with confidence intervals, effect sizes, degrees of freedom and P value noted<br>*Give P values as exact values whenever suitable.*
☒	☐	For Bayesian analysis, information on the choice of priors and Markov chain Monte Carlo settings
☒	☐	For hierarchical and complex designs, identification of the appropriate level for tests and full reporting of outcomes
☐	☒	Estimates of effect sizes (e.g. Cohen's d, Pearson's r), indicating how they were calculated

Our web collection on statistics for biologists contains articles on many of the points above.

Software and code

Policy information about availability of computer code

Data collection	Fluorescence microscopy - LAS-X Life Science Microscope Software (Leica Microsystems),<br>Brightfield microscopy -Olympus cellSens Dimension software v2.1,<br>Animal monitoring system - Promethion Live Software Platform (Sable Systems)<br>Immunoblot densitometry analysis - Image Studio Lite software (LI-COR Biosciences),<br>Dermal thermography - FLIR Research IT Max 4 program (FLIR Systems)<br>LC-MS - Analyst 1.6 AB Sciex.<br>RT-PCR was performed using LightCycler® 480 software, Roche Diagnostics
Data analysis	Image J2 Version 2.14/1.54f (NIH)<br>GraphPad Prism 9 software<br>CellProfiler (Version 3.1.9, Broad institute)<br>The code for single nucleus RNAseq data analysis can be found in the repository: https://doi.org/10.17863/CAM.87955 in standard RDS format. Post processing and analysis were conducted using R (version 4.2.0) R package SeuratObject - version:4.1.3 was used.

For manuscripts utilizing custom algorithms or software that are central to the research but not yet described in published literature, software must be made available to editors and reviewers. We strongly encourage code deposition in a community repository (e.g. GitHub). See the Nature Portfolio guidelines for submitting code & software for further information.

Data

Policy information about availability of data

All manuscripts must include a data availability statement. This statement should provide the following information, where applicable:

- Accession codes, unique identifiers, or web links for publicly available datasets
- A description of any restrictions on data availability
- For clinical datasets or third party data, please ensure that the statement adheres to our policy

The data supporting the findings of this study are available within the paper and its Supplementary Information files. Source data underlying all figures are provided with this paper.

Research involving human participants, their data, or biological material

Policy information about studies with human participants or human data. See also policy information about sex, gender (identity/presentation), and sexual orientation and race, ethnicity and racism.

Reporting on sex and gender	N/A
Reporting on race, ethnicity, or other socially relevant groupings	N/A
Population characteristics	N/A
Recruitment	N/A
Ethics oversight	N/A

Note that full information on the approval of the study protocol must also be provided in the manuscript.

Field-specific reporting

Please select the one below that is the best fit for your research. If you are not sure, read the appropriate sections before making your selection.

☒ Life sciences ☐ Behavioural & social sciences ☐ Ecological, evolutionary & environmental sciences

For a reference copy of the document with all sections, see nature.com/documents/nr-reporting-summary-flat.pdf

Life sciences study design

All studies must disclose on these points even when the disclosure is negative.

Sample size	Sample sizes were pre-determined based on power calculations informed by variability observed in our previously published experiments and consistent with standards in the field. Animal sample sizes were guided by previous studies (PMID: 22000926, 25594176, 33976180, 30413707, 30230471, 38643150, 38378998), and cell staining experiments were guided by previously published work (PMID: 34031592). Group sizes of n = 8–10 mice per treatment condition were estimated to provide >80% statistical power to detect biologically meaningful differences.
Data exclusions	Outliers were excluded only if they exceeded ±2 standard deviations from the group mean or met pre-specified technical failure criteria. Due to slight variability in stereotaxic injections, only AAV-CAG>FLEX-NPY-P2A-GFP injected mice with confirmed increased NPY expression in the arcuate nucleus were included in the analysis.
Replication	Key in vivo findings were reproduced in at least two independent experiments, with each experiment including multiple biological replicates (total sample sizes are indicated in the manuscript). All in vitro experiments were performed independently at least three times. For immunostaining 4-8 brain sections per biological replicate were quantified and averaged.
Randomization	Animals were randomly assigned to treatment groups to minimize allocation bias.
Blinding	Experiments were single-blinded: treatment administration was blinded, while outcome assessments and data analysis were not blinded.

Reporting for specific materials, systems and methods

We require information from authors about some types of materials, experimental systems and methods used in many studies. Here, indicate whether each material, system or method listed is relevant to your study. If you are not sure if a list item applies to your research, read the appropriate section before selecting a response.

Materials & experimental systems

n/a	Involved in the study
[ ]	[x] Antibodies
[ ]	[x] Eukaryotic cell lines
[x]	[ ] Palaeontology and archaeology
[ ]	[x] Animals and other organisms
[x]	[ ] Clinical data
[x]	[ ] Dual use research of concern
[x]	[ ] Plants

Methods

n/a	Involved in the study
[x]	[ ] ChIP-seq
[x]	[ ] Flow cytometry
[x]	[ ] MRI-based neuroimaging

Antibodies

Antibodies used

Rabbit-anti SIK3 (1:300, 39477, Cell Signaling Technology)
Rabbit-anti-SIK1 (pT182)+ SIK2 (PT175)+SIK3 (PT163) antibody (1:1000, EPR19196 Abcam)
Goat-anti rabbit Alexa Fluor 488 (1:500 or 1:1000, A32731, ThermoFisher Scientific)
Goat-anti rabbit Alexa Fluor 594 (1:500, A11012, Thermofisher Scientific)
Rabbit-anti UCP1 (1:300, 14670, Cell Signalling, Technology)
Rabbit-anti Neuropeptide Y (1:500, ab30914, Abcam)
Rabbit-anti Tyrosine hydroxylase (1:500, ab6211, Abcam)
Rabbit-anti CRTC1 (1:300, 2501s, Cell Signaling Technology)
Rabbit-anti HDAC5 (1:300, 20458s, Cell Signaling Technology)
Rabbit-anti-p-AKT (Ser473) [1:300, 9271S, Cell Signaling Technology]
Mouse-anti-Total-AKT [1:1000, 2920, Cell Signaling Technology]
Rabbit-anti-pSTAT3 (Tyr705) [1:300, 9145T, Cell Signaling Technology]
Rabbit-anti-phospho-CRTC1/TORC1 (Ser 151) [1:300, 3359, Cell Signaling Technology]
IRDYE 800 CW Anti-mouse IGG (H+L) (1:5000, 926-32210, Licor-Bio)
IRDYE 680 CW Anti-rabbit GG (H+L) (1:5000, 926-68071, Licor-Bio)

Validation

All primary and secondary antibodies have previously been used by authors and numerous publications as well as the manufacturer companies.

Rabbit-anti-SIK3 cited 3 times webpage: https://www.cellsignal.com/products/primary-antibodies/sik3-antibody/39477?srsltid=AfmBOoqypv_dNzU9b61QoyZwIPRIJegpGYSa9hnExR2oJTA96sRlSE13

Rabbit-anti-UCP1 cited 100 times webpage: https://www.cellsignal.com/products/primary-antibodies/ucp1-d9d6x-rabbit-mab/14670?srsltid=AfmBOooQfocem9JG4vdREDrqbSgSQIe4yzQWhG_RqM38uRbMu7p8l8M2

Rabbit-anti-NPY cited 84 times webpage: https://www.abcam.com/en-us/products/primary-antibodies/neuropeptide-y-antibody-ab30914?srsltid=AfmBOopbU698kBicFRvHxAg2Ruo7nlySc6iLtuc7fiCRsfZx9tzNwJGo

Rabbit-anti-TH cited 77 times webpage: https://www.abcam.com/en-us/products/primary-antibodies/tyrosine-hydroxylase-antibody-ab6211?srsltid=AfmBOopfwHe34mTIg5yQD-ON6pHIXyWJ3krjiGcmFoZZU2-sORVRylvb

Rabbit-anti-CRTC1 cited 11 times webpage: https://www.cellsignal.com/products/primary-antibodies/torc1-crtc1-antibody/2501?srsltid=AfmBOorj3CSrC7sHBo1Cm3Tk69ya4l3yyIAKs5OXXHIwUmbqHwpLHCoV

Rabbit-anti-HDAC5 cited 32 times webpage: https://www.cellsignal.com/products/primary-antibodies/hdac5-d1j7v-rabbit-mab/20458?srsltid=AfmBOopItARMpqTgLRzQeTRaO1TzJ9VcLQlj1M3IApJcR3-CKPTCjlll

Rabbit-anti-p-AKT (Ser473) cited 8010 times webpage: https://www.cellsignal.com/products/primary-antibodies/phospho-akt-ser473-antibody/9271?srsltid=AfmBOooWqkzGz4o0JlwrXTlbLLaNHoqA1lscq-5ykI8sFtGE5QxAPDut

Rabbit-anti-pSTAT3 Tyr705 cited 4305 times webpage: https://www.cellsignal.com/products/primary-antibodies/phospho-stat3-tyr705-d3a7-xp-rabbit-mab/9145?srsltid=AfmBOoq9L8SpiXUaHDlkQFulwJxYFI6P3KxZys2ZMLeaabKt3g03R0aF

Rabbit-anti-phospho-CRTC1/TORC1 (Ser 151) cited 14 times webpage: https://www.cellsignal.com/products/primary-antibodies/phospho-torc1-crtc1-ser151-antibody/3359?srsltid=AfmBOopYKjVjSxgsqua_OYVMklMMGyoTo96OM2ExghgTJlVDdVT_-mC9

Eukaryotic cell lines

Policy information about cell lines and Sex and Gender in Research

Cell line source(s)	The human neuroblastoma cell line SK-N-SH-SY5Y (CRL-2266) was obtained from the ATCC. Cells were maintained under standard culture conditions (37°C, 5% CO_2) in the recommended growth medium.
Authentication	Cell line authentication was conducted by ATCC. https://www.atcc.org/products/crl-2266
Mycoplasma contamination	Not tested for mycoplasma contamination.
Commonly misidentified lines (See ICLAC register)	SK-N-SH-SY5Y cell line is not among those listed as commonly misidentified or contaminated according to the ICLAC database.

Animals and other research organisms

Policy information about studies involving animals; ARRIVE guidelines recommended for reporting animal research, and Sex and Gender in Research

Laboratory animals	C57BL/6J mice (male, aged 8-22 weeks)<br>SIK3lox/lox mice (male, aged 8-22 weeks)<br>SIK3lox/lox mice (female, aged 8-24 weeks)<br>NPYcre/+ mice (male, aged 8-22 weeks)<br>POMCcre/+ mice (male, aged 8-22 weeks)<br>B6.Cg-Gt(ROSA)26Sortm9(CAG-tdTomato)Hze/J mice (male, aged 8-22 weeks)<br>NPYcre/+,SIK3lox/lox mice (male, aged 8-24 weeks)<br>NPYcre/+,SIK3lox/lox mice (female, aged 8-24 weeks)<br>POMCcre/+,SIK3lox/lox mice (male, aged 8-24 weeks)<br>NPYcre/+,Ai9,SIK3lox/lox mice (male, aged 8-16 weeks)<br>POMCcre/+,Ai9,SIK3lox/lox mice (male, aged 8-16 weeks)<br>db/db mice (male, aged 8-16 weeks)<br>db/+ mice (male, aged 8-16 weeks)
Wild animals	This study did not involve the use of wild animals
Reporting on sex	All experiments were performed on male mice unless otherwise stated and are indicated in the results and methods sections. Key experiments were also performed in female mice to assess potential sex differences. Sex was considered in the study design and analysis, and data are reported separately by sex for experiments in which both male and female mice were used. Disaggregated sample sizes for individual experiments are provided in the Source Data files.
Field-collected samples	This study did not utilize field-collected samples
Ethics oversight	Experiments were performed in accordance with protocols approved by the St Vincent's Hospital Animal Ethics Committee (AEC No. 017/19, 027/22, 023/23).

Note that full information on the approval of the study protocol must also be provided in the manuscript.

Plants

Seed stocks	N/A
Novel plant genotypes	N/A
Authentication	N/A
