# Supplementary material for: Targeting hypothalamic SIK3 to promote weight loss and improve glycemic control in mice
Source: Nat Commun. 2026 Jun 2;17:8432. doi: 10.1038/s41467-026-73649-1 (PMC13478199; doi:10.1038/s41467-026-73649-1)
Supplement: Supplementary file 3 [file 41467_2026_73649_MOESM3_ESM.pdf]

nature portfolio

# Peer Review File

# Targeting Hypothalamic SIK3 to Promote Weight Loss and Improve Glycemic Control in Mice

Corresponding Author: Dr Kim Loh

**This file contains all reviewer reports in order by version, followed by all author rebuttals in order by version.**

Version 0:

Reviewer comments:

Reviewer #1

(Remarks to the Author)
This is a well written manuscript, in which the authors show that inactivation of SIK3 in orexigenic NPY neurons reduces food intake, increases energy expenditure, and enhances white adipose tissue browning, resulting in resistance to high-fat diet-induced obesity. The manuscript contains a lot of well performed experiments.

I only have a few points:
1. GLPG3970 have half the weight reducing effect left in the SIK3 mutant mice, indicating that another compartment than NPY neurons or targeting of SIK2 mediate the remainder of the effect. Can the authors clarify this? It is of importance as SIK3 is expressed almost everywhere.
2. Is there a difference in the number of POMC / NPY neurons in the hypothalamus of the mutants?
3. Is the main effect seen in the NPY neuronal SIK3 mutants mediated via altered NPY, GABA or AgRP signaling? There are logical explainations and indications for NPY being involved, for example of reduced NPY expression, but can this be tested directly by e.g. inhibition in the SIK3 mutant?
4. SIK3, but not SIK1/2, is regulated in neurons, but what about the pancreatic islets? It is also important because NPY-cre could be expressed in the beta-cells(?). Is the NPY-cre really specific for the hypothalamus or recombining the floxed locus elsewhere?
5. The CRTC1 and HDAC5 mutants have the opposite phenotype to SIK3 mutant, confirming the reasoning in this manuscript (but also slightly reducing the novelty). Nevertheless, in the discussion the nuclear cytoplasmic ratios of CRTC1/HDAC5 examined in the SK-N-SH-SY5Y cells are mentioned to confirm the link to the downstream effectors. Although the results were also done in vivo ”Consistent with our in vitro findings, we observed that CRTC1 (Figure 6M, 6N and 6O) and HDAC5 (Figure 6P, 6Q and 6R) nuclear-to-cytoplasmic intensity was significantly elevated in the ARC of NPYcre/+,SIK3lox/lox mice.”, so why not include that in the discussion.
6. Seems GLPG3970 is inhibiting both SIK2 and SIK3 at the used doses, something that should be expressed more clearly rather than writing say ”the selective SIK3 inhibitor”.
7. Would be informative to mention the outcome in the phase II clinical trials for inflammatory diseases, so the reader doesn’t need to look that up.

Reviewer #2

(Remarks to the Author)
While the study presents promising findings suggesting hypothalamic SIK3 as a potential therapeutic target for obesity and related metabolic disorders, it suffers from both conceptual and methodological flaws that warrant serious reconsideration.

1. Focus on SIK3 Levels Rather Than Activation:
The central hypothesis of the study is built upon the elevated levels of SIK3 in response to HFD, yet the more biologically relevant question is which SIK homologues are activated under such conditions. Although SIK3 appears to be the only isoform showing increased expression, activation (i.e., phosphorylation status, subcellular localization, or downstream target engagement) is a more accurate indicator of functional involvement. This is a critical oversight, especially when attempting to dissect the role of SIK3 within a specific neuronal population.

2. Questionable Parallel Between Global SIK3 KO and NPY/AgRP-Specific SIK3 KO:
The study heavily relies on the assumption that the metabolic phenotype observed in global SIK3 KO mice is recapitulated in NPY/AgRP neuron-specific SIK3 KO mice. While there are superficial phenotypic similarities—such as resistance to HFD-induced obesity and increased energy expenditure—the causal molecular mechanisms are not adequately delineated in NPY/AgRP neuronal base . The assumption that the absence of SIK3 in NPY/AgRP neurons alone accounts for the moderate decrease in body weight, enhanced BAT thermogenesis and improved metabolic profile (increased leptin and insulin sensitivities) lacks direct and supporting evidences.
3. Mechanistic Gaps and Cell Line Limitations:
There is a methodological inconsistency throughout the study. Mechanistic insights rely on in vitro studies using the SY5Y neuroblastoma cell line, while key physiological findings are derived from in vivo mouse models. The SY5Y model does not accurately reflect NPY/AgRP neurons and limits the translational relevance of these mechanistic claims. Furthermore, the imaging studies used to demonstrate NPY specific co-localization of CRTC1/HDAC5 and their upstream inhibitory kinase SIK3 do not necesserily establish a causal reletionship between them. Beside the confirmation of cytoplasmic retention of these two transcritional co activators in SIK3 overexpressing SY5Y neuroblastoma cells do not confirm or adequetly represent the NPY neurons.
4. Ambiguities in Pharmacological Inhibition Studies:
The long-term administration of a SIK inhibitor is presented as supportive evidence, but this conclusion is confounded by two major issues:
The inhibitor used targets both SIK2 and SIK3, potentially affecting other tissues like the liver (gluconeogenesis), adipose tissue (glucose uptake and browning), and pancreas (insulin secretion). Thus, peripheral effects cannot be excluded.
The study does not demonstrate that the observed effects are NPY neuron-specific, raising the possibility that SIK inhibition in other ARC neuronal subtypes or even non-neuronal cells could contribute to the phenotype. Therefore, the conclusions drawn from this pharmacological approach are speculative at best.
5. Conceptual Disconnect in the Final Paragraph (Lines 625–626):
The final section spanning lines 625–626 contains statements that are not conceptually aligned with the preceding findings. This transition needs to be clarified or revised for consistency.
6. Need for Improved Cell-Type Specific Validation:
While the generation of NPY neuron-specific SIK3 knockout mice is a notable achievement, the study falls short of providing direct evidence that the observed phenotype is specifically due to SIK3 loss in NPY neurons. NATCOMM-level science demands clear mechanistic insight that distinguishes cell-autonomous effects from broader systemic changes. Techniques such as neuron-specific rescue, conditional re-expression, or downstream pathway manipulation would greatly strengthen the claim.
7. Missing Assessment of Hypothalamic Inflammation:
A significant omission is the lack of investigation into hypothalamic inflammation, which is a well-established contributor to obesity-related metabolic dysfunction. Previous reports suggest that mitigating ER stress and inflammatory responses in the hypothalamus has therapeutic potential. Given that GLPG3970, a SIK family inhibitor, is in Phase 2 trials for inflammatory conditions, the role of hypothalamic inflammation in SIK3-mediated metabolic effects should have been at least acknowledged, if not explored.
Conclusion:
This study represents a valuable first step in exploring the role of SIK3 in hypothalamic orexigenic neurons, but the interpretation of its findings is currently limited by key conceptual and methodological oversights. Addressing these issues—particularly the specificity of molecular pathways, validation in relevant cell types, and integration of inflammatory responses—will be essential to move this work toward a more conclusive and impactful contribution.

Reviewer #3

(Remarks to the Author)
This study investigated the role of hypothalamic SIK3 in regulating body weight and glucose homeostasis. The authors observed that SIK3 expression was upregulated in the hypothalamus of obese mice. They further demonstrated that SIK3 inactivation in hypothalamic NPY neurons reduced food intake, improved energy balance, and enhanced white adipose tissue browning. Using a SIK3 inhibitor (GLPG3970), they showed that treatment in diet-induced obese mice decreased food intake and adiposity. Based on these findings, the authors propose that SIK3 inhibition represents a novel therapeutic target for obesity and metabolic disorders. The manuscript is well-organized and logically structured, supported by appropriate references and detailed methodologies. These findings will interest researchers focused on neuronal regulation of metabolism and potential therapies for obesity.

Following are some major comments:
1. GLPG3970 shows significant positive effects on metabolism here, and it is important to discuss the indications and complications of GLPG3970 observed in previous clinical trials, especially considering that the global knockout of Sik3 in mice has detrimental effects and Sik3 seems to be widely expressed in the brain. Additionally, previous studies have highlighted the important role of SIK3 in regulating sleep. These phenotypes could be discussed in the context of the conditional knockout mice model used in this study.
2. Based on the sn-RNA-seq data, Sik3 is expressed in neurons, oligodendrocytes, and tanycytes. To enhance clarity, Figure 1G can include a bar chart illustrating the proportion of double-positive cells (e.g., SIK3+/NPY+ or SIK3+/POMC+) relative to total SIK3+ cells.
3. The use of 12 subtitles might be excessive; consider merging some to enhance reader-friendliness. Additionally, the

sections proposing CRTC1 and HDAC3 as downstream targets could be repositioned later in the text to improve logical flow. Currently, these sections appear disconnected from the preceding and subsequent results, which may hinder readability.
4. The assertion that "SIK3 centrally regulates leptin and insulin signaling through phosphorylation and inhibition of CRTC1 and HDAC5" (Line 413) is considered to be tuned down or proper discussion is needed to clarify this point, as the current data do not directly establish this regulatory mechanism.

Following are some minor points:
1. The bars of Sik1 and Sik2 under the chow diet in Fig 1H seem to be lower than 1. How is the data normalized should be clarified. Also, the housekeeping gene used for normalization in qPCR (Gapdh or ppia) should be specified in legends and explained in Methods.
2. The data presentation in Figs 1L and 1N can be improved. The significance of the dashed lines and inset is unclear. Some of the axes of the bar charts can be better labeled (for example, Fig 3I). The significance of the yellow and white arrows in Figs 6M and 6P should be explained in the legend.
3. The legend of Fig 5 seems overly brief. The time of re-fed (Fig 5B) and how is glucose administered in the glucose tolerance test (5F) should be specified in the legends.
4. The abbreviation SNS can be explained around Line 300.
5. The statement that “This study provides the first evidence that hypothalamic SIK3 signaling plays a key role in promoting development” (Line 511) is not accurate and should be revised.

Version 1:

Reviewer comments:

Reviewer #1

(Remarks to the Author)
thank you for giving clear answers to my points

Reviewer #2

(Remarks to the Author)
The manuscript provides significant mechanistic insight into the role of enhanced SIK3 level/activity in AgRP/NpY neurons in the development of HFD induced obesity using neuron specific SIK3 knockout mouse model and clinical candidate inhibitor studies with DIO mice. The authors’ propose mechanism for the elevated SIK3 activity in orexigenic NpY neurons of HFD fed animals is based on the cytoplasmic sequestration of SIK3’s effector proteins CRTC1 and HDAC4 downstream of impaired insulin and leptin signaling in mediating the observed obesity phenotype. However, the causal links between these pathways are primarily correlative and gathered from different experimental settings. In this vein the reported decrease in NpY level of NpY SIK3 knockout neurons although related to these pathways no mechanistic detail was provided. The study is timely and relevant, especially in light of recent findings showing anti-obesogenic effects of pan-SIK inhibition (e.g., [DOI:10.1161/res.137.suppl_1.Wed084]). While that abstract confirmed the therapeutic potential of SIK inhibition, it did not identify specific isoforms or mechanisms. This manuscript addresses that gap by targeted elimination of SIK3 in orexigenic NpY neurons and SIK2 in POMC neurons respectively albeit to lesser extent. Major Points:
1. Although the initial version holds SIK3 elimination in AgRP\NpY neurons responsible for the improved metabolic phenotype, the revised manuscript appropriately acknowledges possible contributions from SIK2 and SIK3 from other neuronal and none neuronal cells of the brain not excluding the peripheral metabolic tissues that expresses either gene alone or in combination. This balanced interpretation is commendable.
2. Experimental Limitation: Lack of SIK3 Rescue The authors restored NPY expression in NPY-neuron-specific SIK3 KO mice using a Cre-dependent AAV construct, which partially rescued the phenotype. However, a more definitive approach could have addressed the restoration of SIK3 itself in NpY neurons of NPYCre/+;SIK3lox/lox mice. Such an experiment would strengthen the claim that SIK3 absence/inhibition in these neurons is the sole and predominant member responsible for the observed metabolic improvements.
3. in discussion (lines 726-728) authors stated that 'the absence of obesity phenotypes in whole-body SIK2 knockout mice under HFD feeding suggests that SIK2 deficiency alone is insufficient to drive substantial weight loss' However from the statement here one can only drive the conclusion that systemic absence of SIK2 mice are resistant to HFD induced obesity and almost inacceptable to say SIK2'a role in weight loss. Based on the literature nobody has tested SIK2 specific or PAN SIK inhibitor on already obese mice except this study and the recent abstract mentioned above. Since SIK3 inhibitor used in this study targets both SIK2 and SIK3 we can only conclude that both gene have different but finite responsibility for the development of obesity in DIO mouse model (and genetic leptin deficient model).

4. Species Differences The manuscript notes that SIK1–3 expression/activity patterns in mouse metabolic tissues, particularly the liver, differ from human equivalents. This point warrants a precaution to be mentioned in discussion regarding translational relevance and potential limitations for human application.
Overall, this manuscript makes a valuable contribution to understanding the role of NpY specific SIK3 in energy homeostasis and obesity. Addressing the above points would further strengthen its impact and translational significance.

Reviewer #3

(Remarks to the Author)
The revised manuscript has addressed the questions.

Version 2:

Reviewer comments:

Reviewer #2

(Remarks to the Author)
The authors have addressed the methodological and conceptual concerns raised in my previous review. The revisions are sufficient to meet the standards for publication, though the overall contribution remains on the threshold of acceptance.

**Open Access** This Peer Review File is licensed under a Creative Commons Attribution 4.0 International License, which permits use, sharing, adaptation, distribution and reproduction in any medium or format, as long as you give appropriate credit to the original author(s) and the source, provide a link to the Creative Commons license, and indicate if changes were made.
In cases where reviewers are anonymous, credit should be given to 'Anonymous Referee' and the source.
The images or other third party material in this Peer Review File are included in the article's Creative Commons license, unless indicated otherwise in a credit line to the material. If material is not included in the article's Creative Commons license and your intended use is not permitted by statutory regulation or exceeds the permitted use, you will need to obtain permission directly from the copyright holder.
To view a copy of this license, visit https://creativecommons.org/licenses/by/4.0/

Detailed response to reviewers' comments:

**<u>Comments from Reviewer #1:</u>**

This is a well written manuscript, in which the authors show that inactivation of SIK3 in orexigenic NPY neurons reduces food intake, increases energy expenditure, and enhances white adipose tissue browning, resulting in resistance to high-fat diet-induced obesity. The manuscript contains a lot of well performed experiments. I only have a few points:

*Our response: We thank the reviewer for the positive feedback and for recognizing the quality of our work and experiments. Please find our detailed responses to each of the comments below.*

1. GLPG3970 have half the weight reducing effect left in the SIK3 mutant mice, indicating that another compartment than NPY neurons or targeting of SIK2 mediate the remainder of the effect. Can the authors clarify this? It is of importance as SIK3 is expressed almost everywhere.

*Our response: We thank the reviewer for raising this important point. Our data suggest that the weight-reducing effect of GLPG3970 in NPY neuron–specific SIK3 knockout mice likely also involves SIK3 inhibition in other cell types and tissues beyond NPY neurons. SIK3 is broadly expressed in metabolic tissues, as well as in intra- and extrahypothalamic neuronal populations, including the PVN, VMH, DMH and mesolimbic reward centers (VTA, nucleus accumbens) (Steuernagel et.al 2022 Nature Metabolism). Besides, non-neuronal cells such as tanycytes, astrocytes and microglia may also contribute to the appetite and energy balance effects of GLPG3970. We also agree with the reviewer that SIK2 inhibition cannot be excluded, as GLPG3970 is a potent dual inhibitor of SIK2 ($IC_{50}$ = 7.8 nM) and SIK3 ($IC_{50}$ = 3.8 nM), while showing markedly reduced activity toward SIK1 ($IC_{50}$ = 282.8 nM) and no significant off-target activity across a panel of 372 kinases (Peixoto et.al 2024 J Medicinal Chem). The role of SIK2 in appetite and metabolism, however, remains incompletely understood, and future studies using targeted knockout in hypothalamic neurons will be needed to clarify its contribution. We have now added a statement in the Discussion (Lines 704–745) to highlight this limitation, noting that the residual effect of GLPG3970 may result from SIK3 inhibition in other cells/tissues or from SIK2 targeting.*

2. Is there a difference in the number of POMC / NPY neurons in the hypothalamus of the mutants?

*Our response: We did not observe any difference in the number of POMC or NPY neurons in the hypothalamus of the mutant mice. These data have now been added in Supplementary Figure S2K-N (Line 212-215).*

3. Is the main effect seen in the NPY neuronal SIK3 mutants mediated via altered NPY, GABA or AgRP signaling? There are logical explainations and indications for NPY being involved, for example of reduced NPY expression, but can this be tested directly by e.g. inhibition in the SIK3 mutant?

*Our response: We appreciate the reviewer's suggestion. Our studies revealed a significant reduction in arcuate nucleus NPY expression in NPY neuron–specific SIK3 knockout mice,*

Editorial Note: Supplemental Figure 5 in this Peer Review File is reproduced with permission from BioRender, Created in BioRender. Onda, D. A. (2026) https://BioRender.com/mv2x6id

*which may represent a key mechanism contributing to the protection against diet-induced obesity. To further support that the observed phenotype was attributable to altered NPY activity, we selectively overexpressed NPY in HFD-fed wildtype and knockout mice by bilateral administration of a Cre-dependent AAV construct (AAV-CAG>FLEX-NPY-P2A-GFP) (A-D). This approach enabled targeted restoration of NPY expression within the arcuate nucleus (E). Notably, restoring NPY expression abolished the reduction in body weight and adiposity observed in NPY neuron–specific* SIK3 *knockout mice (F-H) by decreasing energy expenditure when compared to control mice (I-L). These findings indicate that the resistance to diet-induced weight gain is mediated, at least in part, by dampened NPY activity, thereby confirming a cell-autonomous role for SIK3 in regulating NPY neuron function. These data have now been added in Supplemental Figure S5A-S5L.*

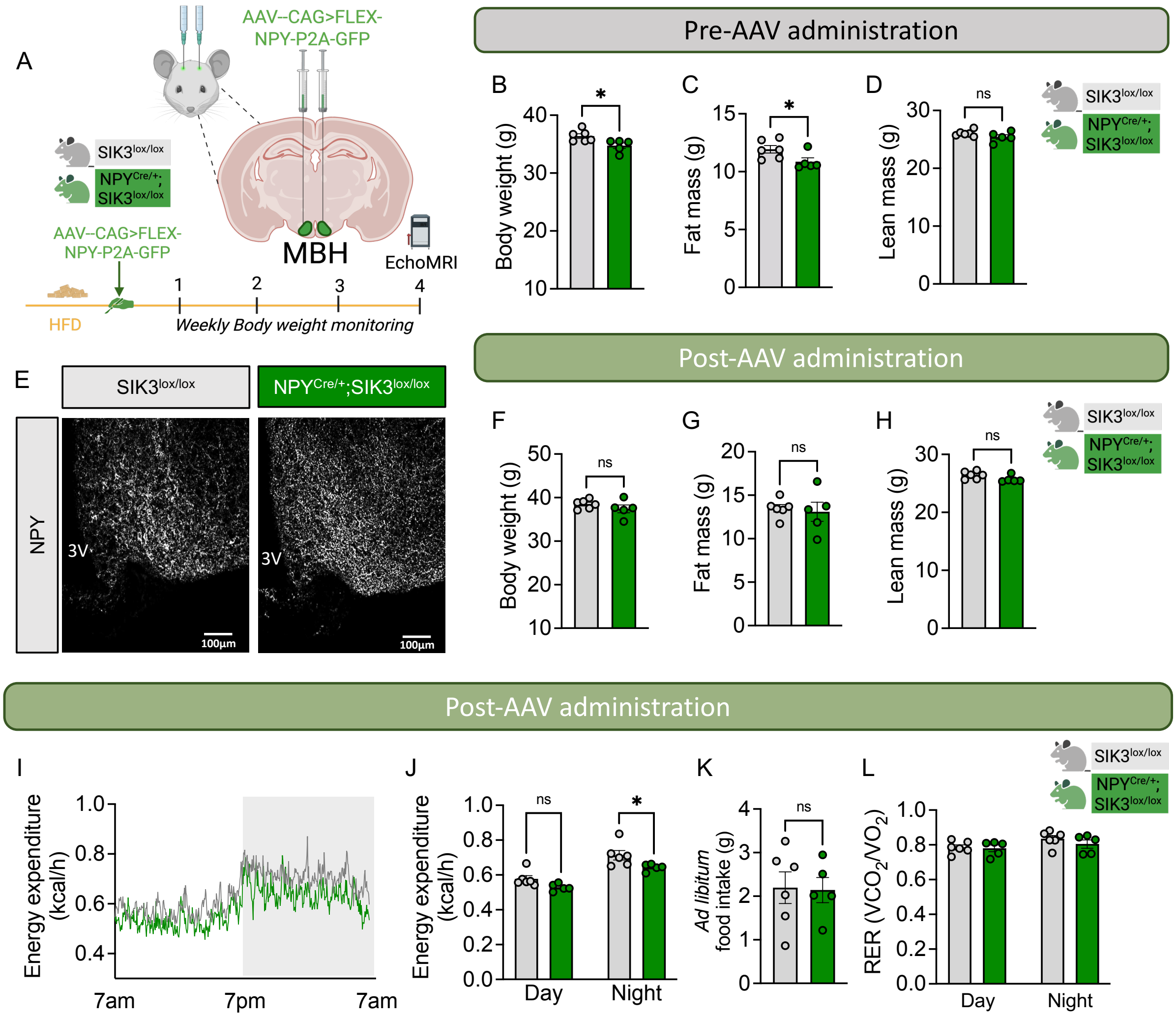


**Supplemental Figure 5. Targeted restoration of NPY in the ARC of NPY$^{cre/+}$;SIK3$^{lox/lox}$ mice reversed the reduced body weight observed in HFD-fed mice.**

(A) Schematic diagram of experimental design. (B) Representative images of NPY in the ARC of SIK3$^{lox/lox}$ and NPY$^{cre/+}$,SIK3$^{lox/lox}$ mice. Scale bar 100 µm. Pre-AAV administration measurements of (C) body weight, (D) fat mass and (E) lean mass in HFD-fed SIK3$^{lox/lox}$ and NPY$^{cre/+}$,SIK3$^{lox/lox}$ mice. 4 weeks post-AAV administration measurements of (F) body weight, (G) fat mass, (H) lean mass, (I)(J) energy expenditure, (K) 24 hours *ad libitum* food intake and (L) respiratory exchange ratio of HFD-fed

SIK3$^{lox/lox}$ and NPY$^{cre/+}$,SIK3$^{lox/lox}$ mice. Data ± s.e.m., n = 5-6 per group. P values by student t test or two-way ANOVA. *$p < 0.05$; **$p < 0.01$; ***$p < 0.001$.

4. SIK3, but not SIK1/2, is regulated in neurons, but what about the pancreatic islets? It is also important because NPY-cre could be expressed in the beta-cells(?). Is the NPY-cre really specific for the hypothalamus or recombining the floxed locus elsewhere?

*Our response: We thank the reviewer for raising this important point. SIK3 is expressed in pancreatic islets, but its regulation and functional role in beta-cells appear to be distinct from those in hypothalamic neurons. To evaluate potential off-target recombination by NPY-Cre, we isolated pancreatic islets from SIK3$^{lox/lox}$ and NPY$^{Cre/+}$;SIK3$^{lox/lox}$ mice and assessed DNA recombination. While recombination was detectable in the islets of NPY$^{Cre/+}$;SIK3$^{lox/lox}$ mice (A), glucose-stimulated insulin secretion (GSIS) at both basal (2.8 mM) and stimulatory (20 mM) glucose concentrations was comparable between genotypes (B). Given that adult β-cells express little NPY, this recombination likely reflects NPY expression in sympathetic nerve fibres or other non-β-cell populations within the islets. Nonetheless, these results are consistent with the notion that the systemic improvements in glycaemic control observed in NPY$^{Cre/+}$;SIK3$^{lox/lox}$ mice are primarily attributable to reduced adiposity and suppression of hepatic glucose production. We have added a statement in the Result (Lines: 343-346) and the data in Supplemental Figure S6A-B to clarify the tissue specificity of NPY-Cre and the limited potential for off-target recombination in pancreatic islets.*

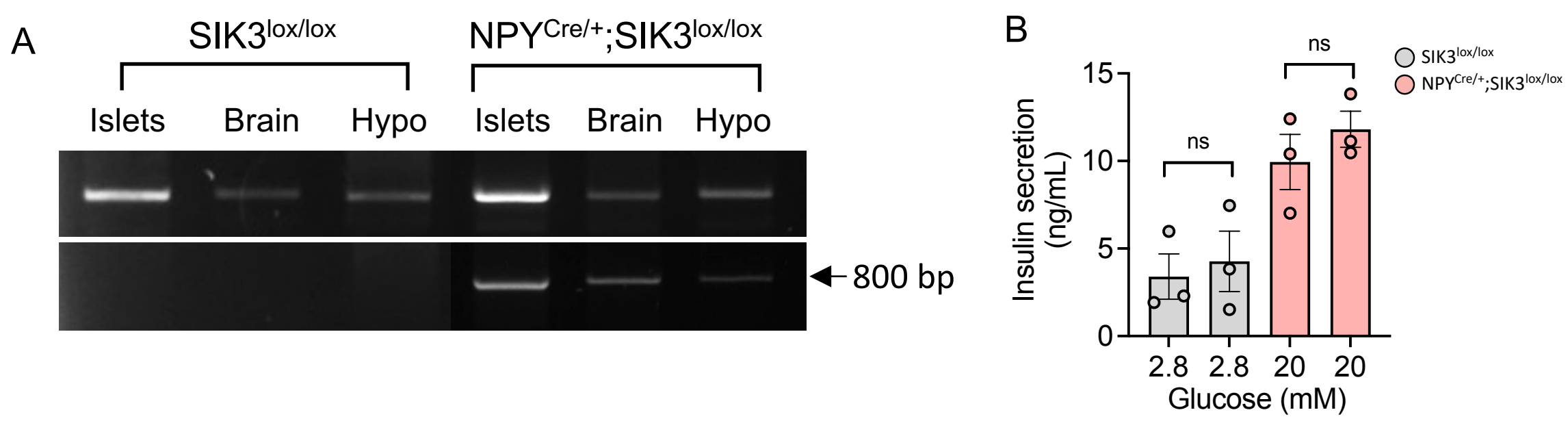


**Supplemental Figure 6. Assessment of DNA recombination and islet insulin secretion in NPY neurons SIK3 knockout mice**
(A) Recombination of the floxed SIK3 allele (~800 bp) with NPY$^{Cre/+}$ in the pancreatic islets, brain, and hypothalamus assessed by PCR. (B) Insulin secretion under basal (2.8 mM) and high (20 mM) glucose stimulation from islets derived from SIK3$^{lox/lox}$ and NPY$^{Cre/+}$;SIK3$^{lox/lox}$ mice. Data are mean± SEM n=3 per group

5. The CRTC1 and HDAC5 mutants have the opposite phenotype to SIK3 mutant, confirming the reasoning in this manuscript (but also slightly reducing the novelty). Nevertheless, in the discussion the nuclear cytoplasmic ratios of CRTC1/HDAC5 examined in the SK-N-SH-SY5Y cells are mentioned to confirm the link to the downstream effectors. Although the results were also done in vivo" Consistent with our in vitro findings, we observed that CRTC1 (Figure 6M, 6N and 6O) and HDAC5 (Figure 6P, 6Q and 6R) nuclear-to-cytoplasmic intensity was significantly elevated in the ARC of NPYcre/+,SIK3lox/lox mice.", so why not include that in the discussion.

*Our response: We have now added this clarification in the Discussion (Lines: 661-668), highlighting that, consistent with our in vitro findings, CRTC1 and HDAC5 nuclear localization is significantly elevated in the arcuate nucleus of $NPY^{Cre/+}$;$SIK3^{lox/lox}$ mice, reinforcing their role as downstream effectors of SIK3 and contributing to the metabolic phenotype observed in these mice.*

6. Seems GLPG3970 is inhibiting both SIK2 and SIK3 at the used doses, something that should be expressed more clearly rather than writing say, “the selective SIK3 inhibitor”.

*Our response: We agree that GLPG3970 is a dual inhibitor of SIK2 and SIK3 at the concentrations used in our study. We have now revised the manuscript to describe GLPG3970 as a SIK2/3 inhibitor rather than a selective SIK3 inhibitor and have included the relevant* $IC_{50}$ *values (SIK3: 3.8 nM; SIK2: 7.8 nM; SIK1: 282.8 nM) to clarify its target specificity.*

7. Would be informative to mention the outcome in the phase II clinical trials for inflammatory diseases, so the reader doesn’t need to look that up.

*Our response: Thank you for this suggestion. In response, we have included a brief summary of the outcomes from the Phase II clinical trials of GLPG3970 in chronic inflammatory diseases in the Discussion (Lines:688-698), which now read: “In previous clinical development, the selective SIK2/3 inhibitor GLPG3970, developed by Galapagos NV, was generally well tolerated and demonstrated preliminary efficacy in patients with psoriasis, with >30 % of patients achieving approximately 50 % reduction in the Psoriasis Area and Severity Index (PASI) compared to none in the placebo arm. In a Phase 2a trial in ulcerative colitis patients, some improvements in endoscopic and histologic parameters were observed in GLPG3970-treated patients, although the primary Mayo Clinic Score endpoint did not significantly differ from placebo. Similarly, no meaningful clinical benefit was observed in a Phase 2a rheumatoid arthritis trial. Across studies, GLPG3970 was generally safe, with mostly mild to moderate adverse events. These findings support the rationale for SIK2/3 inhibition and provide translational context for its investigation in metabolic disease such as obesity and its associated complications.” This provides readers with the clinical trial outcomes directly without the need to consult external sources and publications.*

**Comments from Reviewer #2:**

While the study presents promising findings suggesting hypothalamic SIK3 as a potential therapeutic target for obesity and related metabolic disorders, it suffers from both conceptual and methodological flaws that warrant serious reconsideration.

*Our response: While we fully acknowledge and appreciate the reviewer's perspective, we believe this work provides novel and significant insights into the role of SIK3 in obesity and associated metabolic complications. Specifically, our findings are the first to highlight hypothalamic SIK3 as a potential novel regulator of body weight and energy homeostasis, as well as a potential therapeutic target for obesity, thereby addressing an important knowledge gap in the field. The novelty and inventive nature of this discovery is further underscored by the successful filing of two international PCT patent applications.*

*We have employed a comprehensive approach using in vitro cell-based assays to dissect the underlying signalling pathways, in vivo transgenic KO mouse models to establish physiological relevance and pharmacological studies to assess therapeutic potential. Key additional work included in this revised manuscript includes: 1) To demonstrate consistency and strengthen the translational impact of our work, we have incorporated data from two independent pharmacological inhibitors, including the newly added pan-SIK inhibitor YKL-05-099. The consistency of results observed with both GLPG3970 and YKL-05-099 provides compelling validation of our conclusions (refer to Question 4). 2) To establish a cell-autonomous role, we selectively overexpressed NPY in the NPY-neuron specific SIK3 KO mice using a Cre-dependent AAV construct, allowing targeted restoration of NPY expression. This rescue reversed the metabolic benefits observed in the NPY-neuron specific SIK3 KO mice, confirming that the phenotype is, at least in part, cell-autonomous to NPY neurons (refer to Question 6). 3) Assessment of SIK3 activity levels (refer to Question 1). In addition, we have also included data from female transgenic KO mice (Lines: 362-370. Supplemental Figure S6F-S6S) in the revised manuscript to ensure both sexes are represented and to avoid sex-specific phenotypes. Taken together, we believe the novelty, methodological rigor and translational relevance of our study provide a strong foundation supporting our conclusions. We hope that the additional data and revisions have addressed the reviewer's concerns and strengthened the manuscript and enhanced its suitability for publication.*

1. Focus on SIK3 Levels Rather Than Activation: The central hypothesis of the study is built upon the elevated levels of SIK3 in response to HFD, yet the more biologically relevant question is which SIK homologues are activated under such conditions. Although SIK3 appears to be the only isoform showing increased expression, activation (i.e., phosphorylation status, subcellular localization, or downstream target engagement) is a more accurate indicator of functional involvement. This is a critical oversight, especially when attempting to dissect the role of SIK3 within a specific neuronal population.

*Our response: We thank the reviewer for highlighting this important point. We agree that both activation status and protein/mRNA expression are critical for establishing the functional involvement of hypothalamic SIK3 in obesity. Our initial focus on SIK3 was guided by analyses from single-nucleus RNA sequencing databases of the hypothalamus (Steuernagel et.al 2022*

*Nature Metabolism), which demonstrated that SIK3 is the predominant isoform expressed in the brain, specifically in hypothalamic neurons and glia cells. While we found that SIK3 expression is upregulated in obese mouse models, we also recognize the importance of directly assessing SIK3 kinase activation. However, due to the limited availability of high-quality phospho-specific antibodies, examining SIK3 phosphorylation in the hypothalamus has been challenging.*

*To address this, we have now employed a pan-phospho SIK antibody [Anti-SIK1 (phospho T182) + SIK2 (phospho T175) + SIK3 (phospho T163); Abcam EPR19196] and first validated its specificity in control and SIK3-overexpressing SH-SY5Y cells. As expected, total SIK3 protein levels (~150 kDa) were increased in SIK3-overexpressing cells compared to control (J). Using the pan-phospho SIK antibody, we also detected a stronger band at ~150 kDa corresponding to phosphorylated SIK3 (J). We then applied this approach to hypothalamic lysates from chow- and HFD-fed mice. Importantly, we found that both phosphorylated SIK3 and total SIK3, when normalized to actin, were significantly upregulated in the hypothalamus of HFD-fed obese mice (K-M). This indicates that the increased abundance and absolute levels of hypothalamic SIK3 expression, rather than changes in its activation, are likely driving the development of obesity and consistent with SIK3 functioning as a constitutively active kinase. We have added a statement in the Result (Lines: 184-187, Supplemental Figure S1J-S1M).*

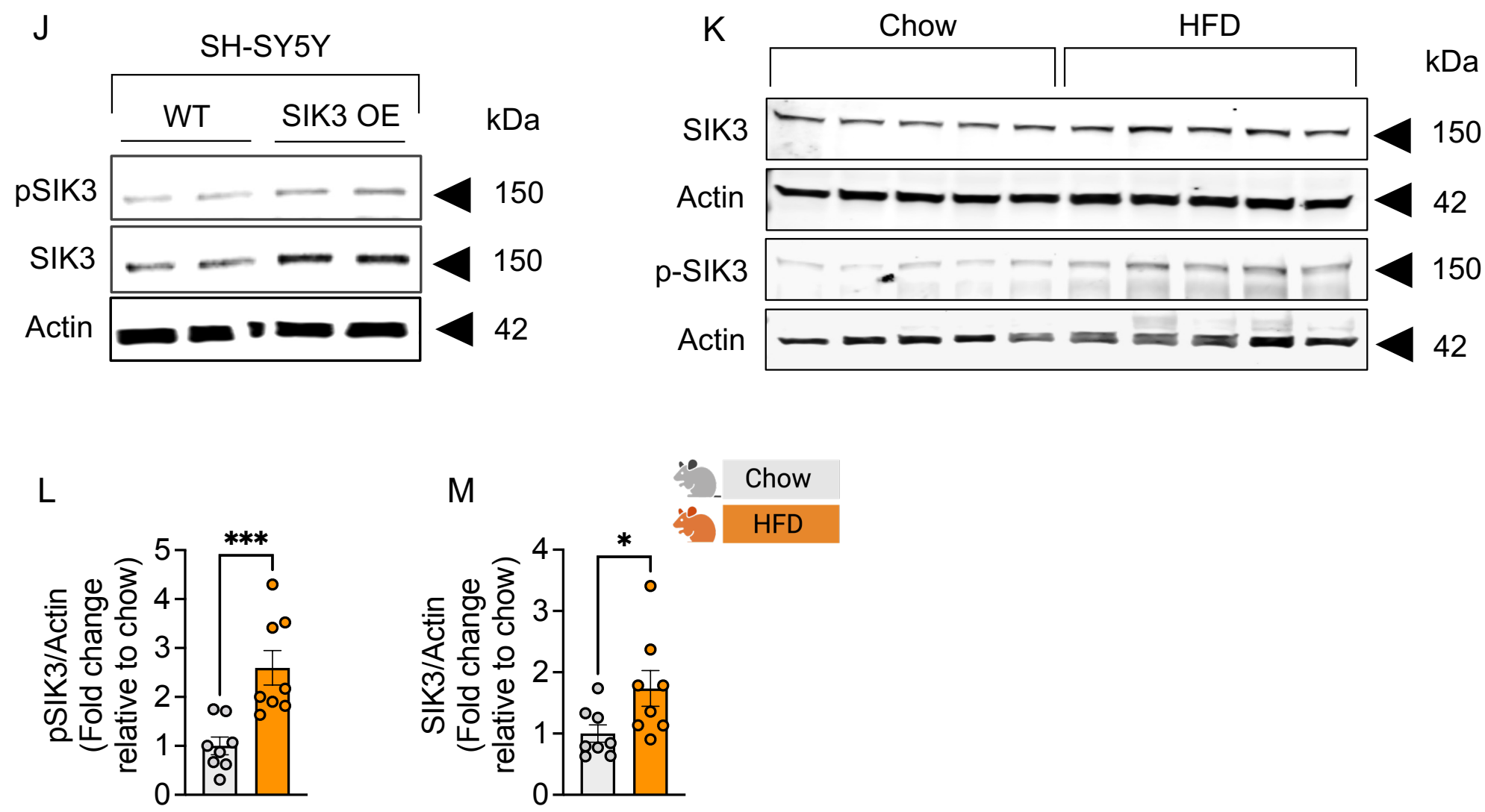


**Supplemental Figure 1.** (J) Immunoblot of phosphorylated SIK3 and total SIK3 in WT and SIK3 overexpressing SK-N-SH-SY5Y neuroblastoma cells. (K) Representative immunoblots of phosphorylated SIK3 and total SIK3 in chow and HFD hypothalamus. (L) Densitometry analysis of phosphorylated SIK3 in the hypothalamus of chow and HFD-fed mice. (M) Densitometry analysis of total SIK3 in the hypothalamus of chow and HFD-fed mice. Data are mean ± s.e.m, n=8 per group.

2. Questionable Parallel Between Global SIK3 KO and NPY/AgRP-Specific SIK3 KO:
The study heavily relies on the assumption that the metabolic phenotype observed in global SIK3 KO mice is recapitulated in NPY/AgRP neuron-specific SIK3 KO mice. While there are superficial phenotypic similarities—such as resistance to HFD-induced obesity and increased

energy expenditure—the causal molecular mechanisms are not adequately delineated in NPY/AgRP neuronal base. The assumption that the absence of SIK3 in NPY/AgRP neurons alone accounts for the moderate decrease in body weight, enhanced BAT thermogenesis and improved metabolic profile (increased leptin and insulin sensitivities) lacks direct and supporting evidence.

*Our response: We would like to reiterate that the majority of global SIK3 knockout mice die within the first week after birth and exhibit developmental abnormalities, dwarfism and markedly reduced body weight, including decreased lean mass, even under basal chow-fed conditions. As such, it is misleading to draw definitive conclusions from the global SIK3 deletion model regarding the specific role of hypothalamic SIK3 in body weight and metabolic regulation. In contrast, our conditional knockout model specifically targets SIK3 in orexigenic NPY neurons, which do not display developmental abnormalities and show comparable metabolic parameters to wild-type littermates under basal chow-fed conditions. The resistance to diet-induced weight gain phenotype becomes evident only under high-fat diet feeding, thereby avoiding systemic confounders and allowing us to delineate the neuron-specific role of SIK3 in the regulation of energy homeostasis. Consistent with this, the magnitude of effects on body weight, adiposity and glucose homeostasis in NPY neuron–specific SIK3 knockout mice was more modest than that observed in the global SIK3 knockout, suggesting that additional SIK3-dependent mechanisms in other neuronal populations or peripheral tissues may also contribute to the overall phenotype. Furthermore, the NPY/AgRP specificity of our findings is supported by the observation that deletion of SIK3 in POMC neurons did not result in significant phenotypic changes under high-fat diet feeding. Therefore, we agree with the reviewer that the global SIK3 knockout phenotype cannot be fully ascribed to NPY/AgRP neuronal mechanisms alone. Accordingly, we have revised the text to clarify that the NPY neuron–specific SIK3 knockout represents a partial phenocopy of the global SIK3 knockout model. We also acknowledge that future studies involving targeted deletion of SIK3 in other neuronal populations/hypothalamic regions or peripheral tissues will be necessary to elucidate the tissue-specific roles of SIK3 in body-weight regulation and to further define their contribution to the overall global SIK3 phenotype (Line 731-745).*

*Importantly, our molecular and physiological analyses support a causal role for SIK3 deficiency in NPY neurons in conferring resistance to diet-induced obesity. Specifically, NPY-neuron–specific SIK3 knockout mice exhibited reduced NPY expression in both the arcuate nucleus and second-order neurons within the paraventricular nucleus, along with elevated tyrosine hydroxylase levels in the paraventricular nucleus and brown adipose tissue. These alterations were accompanied by increased brown adipose tissue temperature and upregulation of thermogenic genes (e.g., Ucp1), indicative of an enhanced central thermogenic drive that likely contributes to increased energy expenditure and reduced adiposity and weight gain. At the molecular level, leptin and insulin stimulation resulted in enhanced phosphorylation of AKT and STAT3 within the arcuate nucleus, specifically in NPY neurons (as shown by Ai9–pAKT/pSTAT3 co-localisation) in the knockout mice, demonstrating heightened intracellular leptin and insulin signalling in these neurons. Notably, the insulin-induced AKT phosphorylation observed in NPY-neuron–specific SIK3 knockout mice was*

*abolished upon administration of an insulin receptor antagonist, confirming that the effect is insulin receptor signalling dependent. The enhanced central insulin signalling observed with SIK3 loss in NPY neurons aligns with reduced hepatic gluconeogenesis, as demonstrated by the gold standard assay hyperinsulinemic–euglycemic clamp studies, and contributes to improved overall glycaemic control. However, we agree with the reviewer that additional studies are required to delineate the direct regulatory mechanisms by which SIK3 modulates leptin and insulin signalling in the hypothalamus. Accordingly, we have tempered our conclusions in the revised manuscript, using terms such as "associated with" and have clarified that further investigations are warranted to determine how SIK3 directly influences these hormonal signalling pathways (Line 681-684).*

3. Mechanistic Gaps and Cell Line Limitations:
There is a methodological inconsistency throughout the study. Mechanistic insights rely on in vitro studies using the SY5Y neuroblastoma cell line, while key physiological findings are derived from in vivo mouse models. The SY5Y model does not accurately reflect NPY/AgRP neurons and limits the translational relevance of these mechanistic claims. Furthermore, the imaging studies used to demonstrate NPY specific co-localization of CRTC1/HDAC5 and their upstream inhibitory kinase SIK3 do not necessarily establish a causal relationship between them. Beside the confirmation of cytoplasmic retention of these two transcriptional co activators in SIK3 overexpressing SY5Y neuroblastoma cells do not confirm or adequately represent the NPY neurons.

*Our response: We thank the reviewer for this insightful comment and acknowledge that the SH-SY5Y neuroblastoma cell line does not fully recapitulate the molecular and physiological characteristics of hypothalamic NPY neurons. Our rationale for using this model was to complement our in vivo findings and to provide an alternative cellular system for assessing the direct molecular relationship between SIK3 and its known downstream targets: CRTC1 and HDAC5, in a neuronal context. The SH-SY5Y line is widely used in neuroendocrine and neuronal signalling research due to its neuronal lineage properties and high transfection efficiency, which make it well suited for mechanistic studies of kinase-mediated pathways. Importantly, overexpression of SIK3 in SH-SY5Y cells led to increased cytoplasmic retention of CRTC1 and HDAC5, whereas pharmacological inhibition of SIK3 significantly promoted their nuclear translocation. These in vitro findings support and are consistent with our in vivo observations, which demonstrate that nuclear localisation of CRTC1 and HDAC5 in NPY neurons (validated using Ai9 reporter mice) was significantly increased following SIK3 ablation, reinforcing our mechanistic conclusions. To further strengthen our results, we have also now shown that in our in vivo pharmacological model, GLPG3970-treated mice exhibited significantly elevated nuclear accumulation of CRTC1 and HDAC5 compared with controls (Lines: 389-391, Supplemental Figures S7C-S7H). Furthermore, we have included additional discussion in the revised manuscript highlighting the limitations of the SH-SY5Y model and noting that future studies using primary hypothalamic neuronal cultures will be valuable to further confirm and extend these mechanistic insights under more physiologically relevant conditions (Lines: 661-665).*

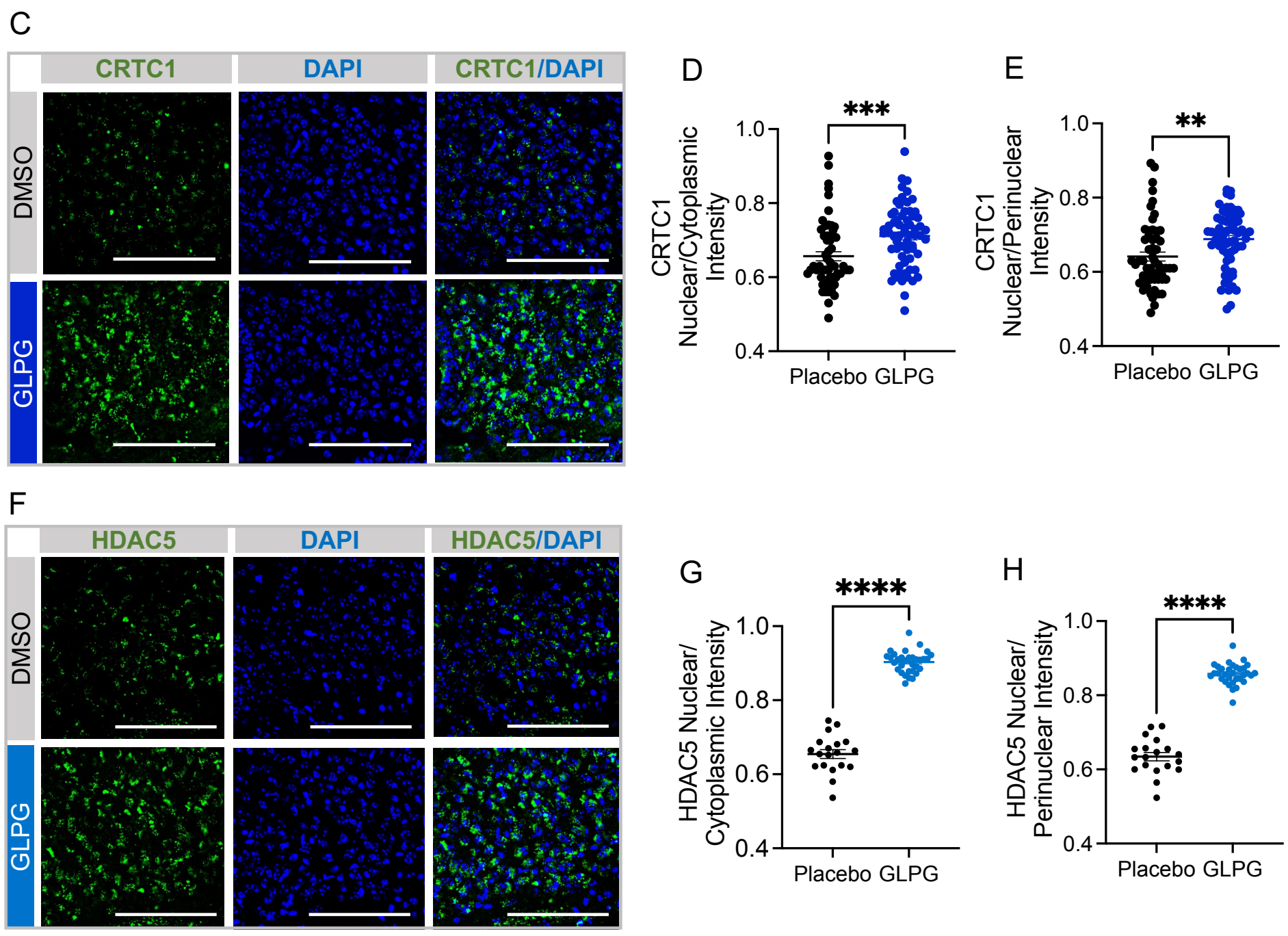


**Supplemental Figure 7.** (C) Immunostaining of CRTC1 in placebo and GLPG3970 treated HFD-fed mice. (D) CRTC1 Nuclear/Cytoplasmic ratio and (E) CRTC1 Nuclear/Perinuclear ratio. (F) HDAC5 immunostaining in placebo and GLPG3970 treated HFD-fed mice. (G) HDAC5 Nuclear/Cytoplasmic ratio. (H) HDAC5 Nuclear/Perinuclear ratio. Scale bar, 100 µm. Data ± s.e.m.. P values by student t test. *$p < 0.05$; **$p < 0.01$; ***$p < 0.001$.

4. Ambiguities in Pharmacological Inhibition Studies:
The long-term administration of a SIK inhibitor is presented as supportive evidence, but this conclusion is confounded by two major issues: The inhibitor used targets both SIK2 and SIK3, potentially affecting other tissues like the liver (gluconeogenesis), adipose tissue (glucose uptake and browning), and pancreas (insulin secretion). Thus, peripheral effects cannot be excluded. The study does not demonstrate that the observed effects are NPY neuron-specific, raising the possibility that SIK inhibition in other ARC neuronal subtypes or even non-neuronal cells could contribute to the phenotype. Therefore, the conclusions drawn from this pharmacological approach are speculative at best.

*Our response: We agree with the reviewer that pharmacological approaches, such as the use of GLPG3970, do not allow full discrimination between SIK2- and SIK3-dependent effects, nor between central and peripheral mechanisms, and we have initially clarified this limitation explicitly in the discussion section. While peripheral contributions cannot be fully excluded with systemic pharmacological inhibition approaches, several lines of evidence support a central, neuronally mediated mechanism. 1) In line with our mass spectrometry data demonstrating that GLPG3970 is detectable in the hypothalamus following oral administration, we engaged a medicinal chemist to evaluate its CNS exposure properties. GLPG3970 has a CNS multi-parameter optimization (MPO) score of 4.0, which is indicative of favourable CNS penetration. While there is no strict universal cutoff for MPO scores in therapeutics with CNS activity, scores around 4.0 generally correlate with compounds capable*

*of achieving meaningful CNS exposure. Together, these findings support that GLPG3970 reaches the central nervous system, reinforcing the plausibility of the central SIK3-mediated effects observed in our pharmacological studies. 2) Both systemic GLPG3970 treatment and NPY-neuron–specific SIK3 knockout mice exhibit reduced adiposity, attributable to both reduced energy intake and increased energy expenditure. Indeed, our newly conducted pair-feeding experiments demonstrated that GLPG3970-treated mice lost slightly more weight than pair-fed controls consuming identical amounts of food, indicating that increased energy expenditure, in addition to reduced caloric intake, contributes to the observed phenotype (Lines: 529-532, Supplemental Figure S10M and S10N). Notably, GLPG3970 administration in NPY-neuron–specific SIK3 knockout mice resulted in only approximately 10% weight loss compared to ~22% in wild-type controls, indicating that SIK3/NPY neurons, at least in part, mediate a substantial component of the effect. 3) Systemic GLPG3970 treatment also increased leptin-induced pSTAT3 in the arcuate nucleus, closely mirroring the effects observed in the NPY-neuron–specific SIK3 knockout model. Moreover, we also observed significantly reduced fasting leptin serum in the GLPG3970-treated mice. Combined leptin and GLPG3970 treatment produced a greater and more rapid reduction in body weight than either intervention alone (Lines:537-541 Supplemental Figure S11F-S11I), supporting a central, leptin-dependent component. Collectively, these findings suggest that the metabolic benefits of systemic SIK3 inhibition include a central, neuron-mediated component, with potential contributions also likely from peripheral actions. The discussion has been revised to explicitly reflect this interpretation and its associated limitations (Lines:704-745).*

*We would like to emphasize that the rationale for conducting the pharmacological inhibition study was to provide complementary and translationally relevant evidence, offering preclinical proof of concept that systemic inhibition of SIK3 reduces body weight, adiposity and improving glycaemic control. To further strengthen the pharmacological studies and enhance the translational relevance of our findings, we have incorporated data from two independent pharmacological inhibitors, including the newly added pan-SIK inhibitor YKL-05-099 (Line:473-481, Supplemental Figure S9A-S9O). The consistency of metabolic outcomes observed with both GLPG3970 and YKL-05-099, including reduced body weight and adiposity, decreased food intake and improved glucose homeostasis, provides compelling evidence supporting our conclusions and demonstrates that these effects are reproducible across distinct pharmacological approaches.*

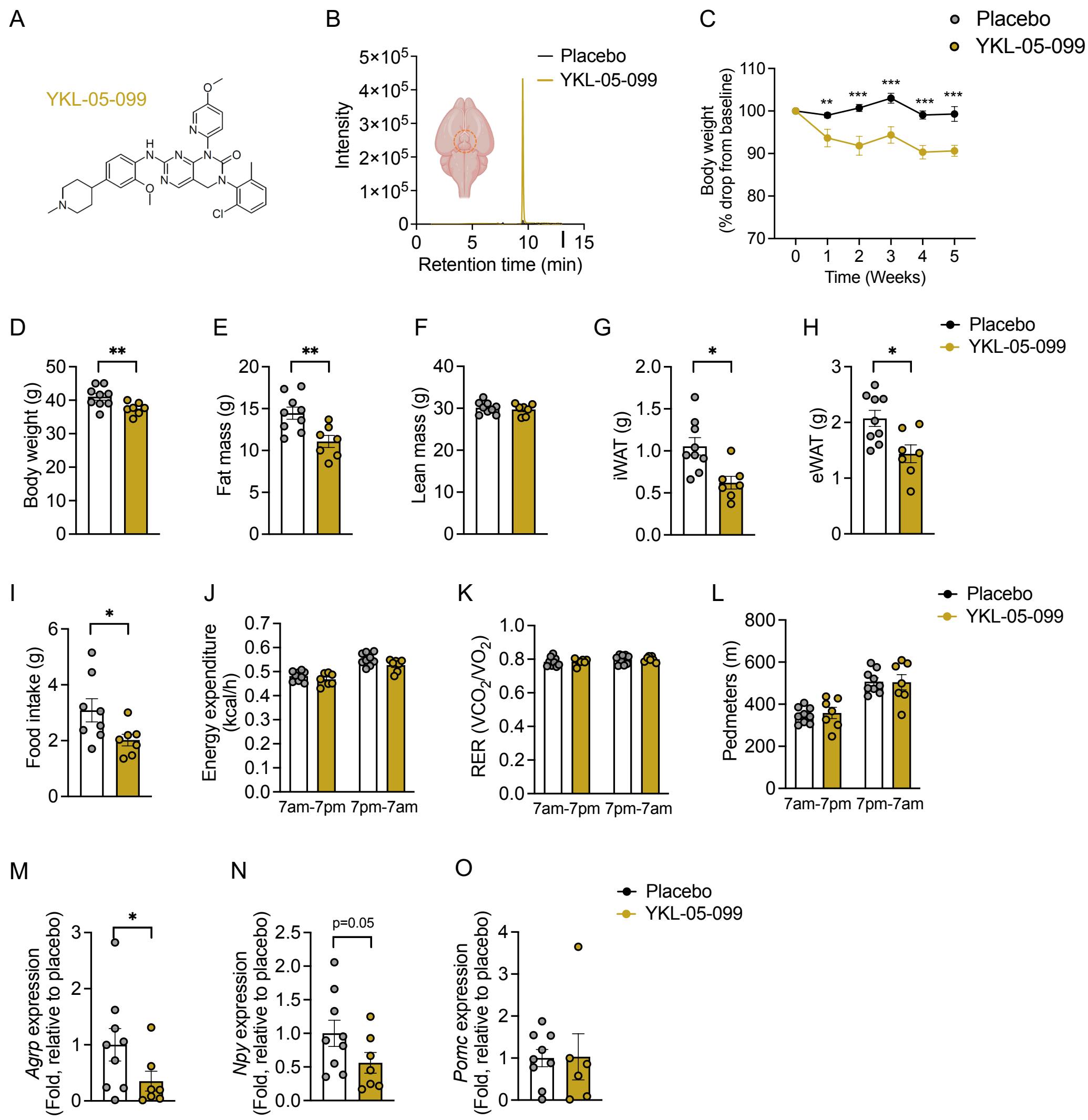


**Supplemental Figure 9. The pan-SIK inhibitor YKL-05-099 promotes weight loss and suppresses food intake in HFD-fed obese mice.**
(A) The chemical structure of YKL-05099. (B) Qualitative chromatograms showing detection of YKL-05-099 in mouse hypothalamus. (C) Body weight % drop from baseline. (D) Body weight. (E) Fat mass. (F) Lean mass. Dissected weights of individual fat depots from (G) iWAT (H) eWAT. (I) Daily food intake. (J) Energy expenditure. (K) Respiratory exchange ratio. (L) Pedmeters. Whole hypothalamic (M) *Agrp*, (N) *Npy,* (O) *Pomc* gene expression in HFD-fed C57BL/6 mice treated with placebo or YKL-05-099 (18 mg/kg, oral, once daily for 5 weeks). Data ± s.e.m., n = 7-9 per group. P values by two-way ANOVA and student's t test. *$p < 0.05$; **$p < 0.01$; ***$p < 0.001$.

5. Conceptual Disconnect in the Final Paragraph (Lines 625–626):
The final section spanning lines 625–626 contains statements that are not conceptually aligned with the preceding findings. This transition needs to be clarified or revised for consistency.

*Our response: We thank the reviewer for this comment. We have revised the final paragraph to improve conceptual alignment with the preceding findings. The original intent of these sentences was to integrate evidence from both genetic and pharmacological studies, suggesting*

*that the weight loss and reduced adiposity induced by GLPG3970 are at least partly attributable to SIK3 inhibition in NPY neurons. We have now clarified this statement to explicitly convey that, while SIK3 inhibition in NPY neurons likely mediates a significant component of the observed effects, contributions from other neuronal populations and peripheral tissues cannot be excluded. The revised text (Lines: 704-719) now reads: "Although NPY neurons represent a key neuron through which SIK3 regulates energy balance, the metabolic effects of GLPG3970 cannot be attributed exclusively to SIK3 inhibition within this neuronal population. GLPG3970 was administered orally and reaches both the central nervous system and peripheral organs, raising the likelihood of targeting multiple central and peripheral sites. SIK3 is widely expressed across numerous hypothalamic and extrahypothalamic regions including the PVN, VMH, DMH, ventral tegmental area and nucleus accumbens, and therefore GLPG3970 may influence broader neural circuits governing appetite and energy expenditure. In addition, non-neuronal cell types such as tanycytes, astrocytes and microglia, all of which express SIK3, may contribute to changes in central nutrient sensing and neuroinflammatory tone. Beyond the CNS, SIK3 is highly expressed in peripheral metabolic tissues including liver and skeletal muscle, and pharmacological inhibition by GLPG3970 in these tissues could enhance energy expenditure independently of NPY neuron signalling. Thus, while NPY neuron–specific SIK3 deletion partially blunts the efficacy of GLPG3970, the compound's potent systemic effects likely reflect integrated actions across multiple neuronal and peripheral cell types rather than a solely NPY-dependent mechanism." This revision ensures conceptual consistency and a balanced interpretation of the data.*

6. Need for Improved Cell-Type Specific Validation:
While the generation of NPY neuron-specific SIK3 knockout mice is a notable achievement, the study falls short of providing direct evidence that the observed phenotype is specifically due to SIK3 loss in NPY neurons. NATCOMM-level science demands clear mechanistic insight that distinguishes cell-autonomous effects from broader systemic changes. Techniques such as neuron-specific rescue, conditional re-expression, or downstream pathway manipulation would greatly strengthen the claim.

*Our response: We thank the reviewer for highlighting the importance of mechanistic validation of cell-autonomous effects. Our studies revealed a significant reduction in arcuate nucleus NPY expression in NPY neuron–specific SIK3 knockout mice, which may represent a key mechanism contributing to the protection against diet-induced obesity. To further support that the observed phenotype was attributable to altered NPY activity, we selectively overexpressed NPY in HFD-fed wildtype and knockout mice by bilateral administration of a Cre-dependent AAV construct (AAV-CAG>FLEX-NPY-P2A-GFP) (A-D). This approach enabled targeted restoration of NPY expression within the arcuate nucleus (E). Notably, restoring NPY expression abolished the reduction in body weight and adiposity observed in NPY neuron–specific* SIK3 *knockout mice by decreasing energy expenditure when compared to control mice (F-L). These findings indicate that the resistance to diet-induced weight gain is mediated, at least in part, by dampened NPY activity, thereby confirming a cell-autonomous role for SIK3 in regulating NPY neuron function. These data have now been added in Supplemental Figure S5A-S5L.*

Editorial Note: Supplemental Figure 5 in this Peer Review File is reproduced with permission from BioRender, Created in BioRender. Onda, D. A. (2026) https://BioRender.com/mv2x6id

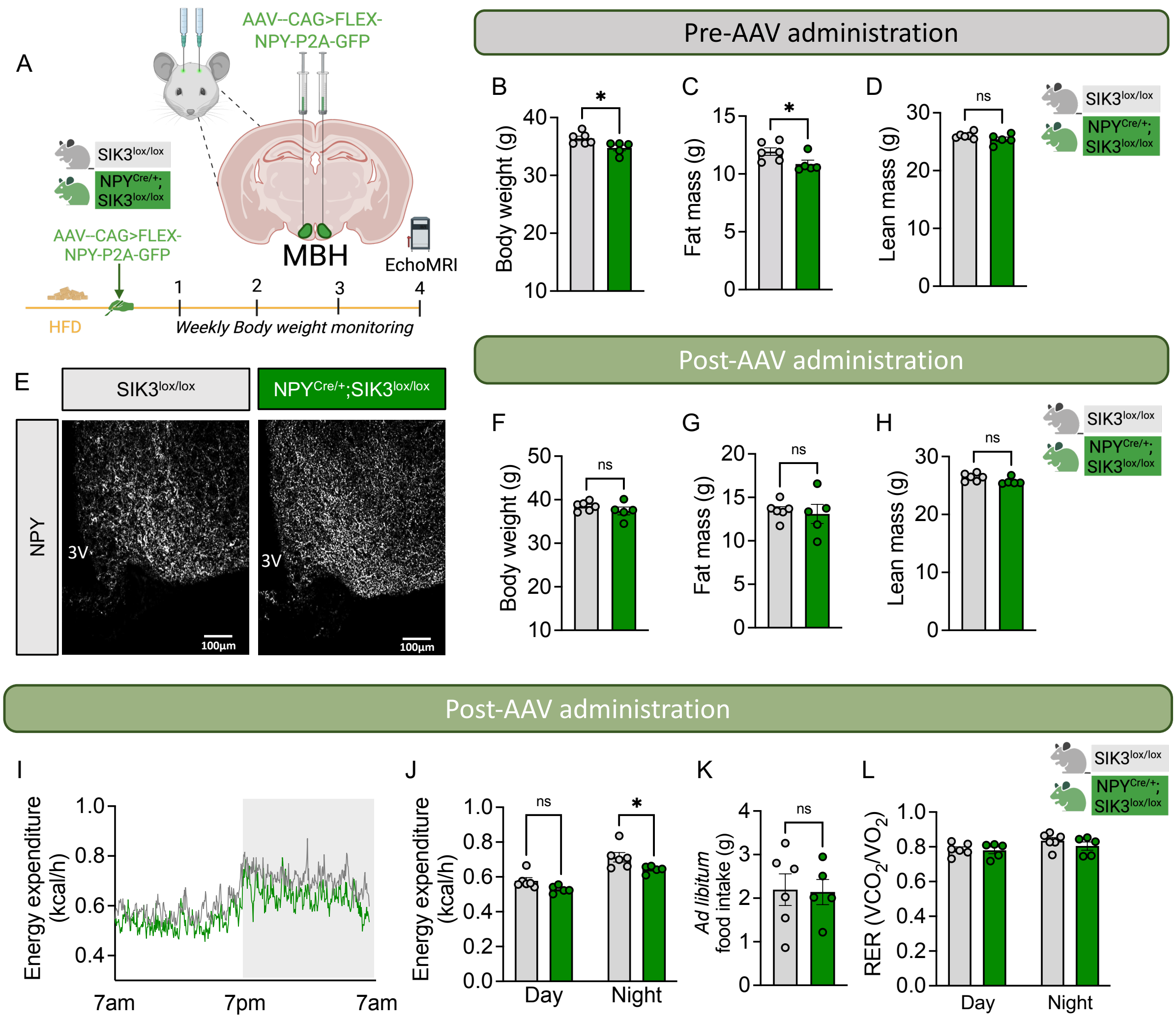


**Supplemental Figure 5. Targeted restoration of NPY in the ARC of $NPY^{cre/+}$;$SIK3^{lox/lox}$ mice reversed the reduced body weight observed in HFD-fed mice.**
(A) Schematic diagram of experimental design. (B) Representative images of NPY in the ARC of $SIK3^{lox/lox}$ and $NPY^{cre/+}$,$SIK3^{lox/lox}$ mice. Scale bar 100 µm. Pre-AAV administration measurements of (C) body weight, (D) fat mass and (E) lean mass in HFD-fed $SIK3^{lox/lox}$ and $NPY^{cre/+}$,$SIK3^{lox/lox}$ mice. 4 weeks post-AAV administration measurements of (F) body weight, (G) fat mass, (H) lean mass, (I)(J) energy expenditure, (K) 24 hours *ad libitum* food intake and (L) respiratory exchange ratio of HFD-fed $SIK3^{lox/lox}$ and $NPY^{cre/+}$,$SIK3^{lox/lox}$ mice. Data ± s.e.m., n = 5-6 per group. P values by student t test or two-way ANOVA. *$p < 0.05$; **$p < 0.01$; ***$p < 0.001$.

7. Missing Assessment of Hypothalamic Inflammation:
A significant omission is the lack of investigation into hypothalamic inflammation, which is a well-established contributor to obesity-related metabolic dysfunction. Previous reports suggest that mitigating ER stress and inflammatory responses in the hypothalamus has therapeutic potential. Given that GLPG3970, a SIK family inhibitor, is in Phase 2 trials for inflammatory conditions, the role of hypothalamic inflammation in SIK3-mediated metabolic effects should have been at least acknowledged, if not explored.

*Our response: We thank the reviewer for raising this important point. Accordingly, we have extended our analysis to assess hypothalamic inflammation by measuring mRNA expression of key inflammatory markers, including Tnfα, Il6 and Ikkβ in the hypothalamus of GLPG3970-treated mice. Expression of these markers was significantly reduced compared to placebo, indicating that pharmacological inhibition of SIK3 by GLPG3970 attenuates hypothalamic inflammatory signalling. These findings are consistent with the previous reported anti-inflammatory effects of GLPG3970 in immune cells and suggest that reduced hypothalamic inflammation may contribute to the metabolic improvements observed in our study. The new data have been incorporated into the revised manuscript (Lines:532-535, Supplemental Figure S11A-S11E).*

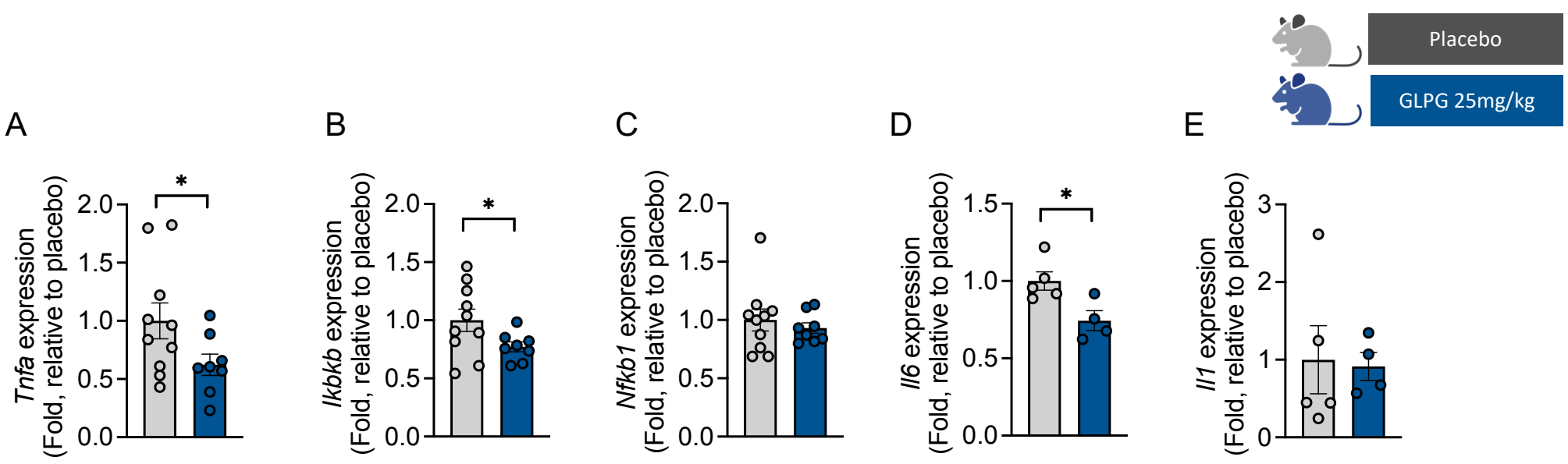


**Supplemental Figure 11.** Hypothalamic (A) *Tnfα*, (B) *Ikbkb,* (C) *Nfkb1,* (D) *Il6, and (E) Il1* mRNA expression in placebo and GLPG3970-treated HFD-fed mice. Data ± s.e.m., n = 4-10 per group. P values by student t test or two-way ANOVA. *p < 0.05; **p < 0.01; ***p < 0.001.

**Comments from Reviewer #3:**

This study investigated the role of hypothalamic SIK3 in regulating body weight and glucose homeostasis. The authors observed that SIK3 expression was upregulated in the hypothalamus of obese mice. They further demonstrated that SIK3 inactivation in hypothalamic NPY neurons reduced food intake, improved energy balance, and enhanced white adipose tissue browning. Using a SIK3 inhibitor (GLPG3970), they showed that treatment in diet-induced obese mice decreased food intake and adiposity. Based on these findings, the authors propose that SIK3 inhibition represents a novel therapeutic target for obesity and metabolic disorders. The manuscript is well-organized and logically structured, supported by appropriate references and detailed methodologies. These findings will interest researchers focused on neuronal regulation of metabolism and potential therapies for obesity.

*Our response: We thank the reviewer for the positive and encouraging comments. We appreciate the recognition of our study's clear structure, rigorous methodology and significance in identifying hypothalamic SIK3 as a novel regulator of energy homeostasis.*

1. GLPG3970 shows significant positive effects on metabolism here, and it is important to discuss the indications and complications of GLPG3970 observed in previous clinical trials, especially considering that the global knockout of Sik3 in mice has detrimental effects and Sik3 seems to be widely expressed in the brain. Additionally, previous studies have highlighted the important role of SIK3 in regulating sleep. These phenotypes could be discussed in the context of the conditional knockout mice model used in this study.

*Our response: Accordingly, we have now included additional discussion on the clinical indications and reported outcomes of GLPG3970 from three Phase 2 clinical trials (psoriasis, ulcerative colitis and rheumatoid arthritis) (Lines:688-698). While our pre-clinical proof-of-concept studies demonstrated that GLPG3970 promotes weight loss and improves obesity-associated complications, we acknowledge that it was originally developed for autoimmune and chronic inflammatory indications and was reported to be well tolerated. We also note that global SIK3 deletion in mice leads to developmental abnormalities and a dwarfism phenotype. In contrast, our conditional knockout model specifically targets SIK3 in NPY neurons, thereby avoiding systemic effects and allowing us to delineate its neuron-specific role in metabolic regulation.*

*Furthermore, we have expanded the discussion to include these elegant studies from several groups on the established role of SIK3 in sleep regulation (Funato et.al 2016 Nature, Honda et.al 2018 PNAS, Wang et.al 2018 Nature, Iwasaki et.al 2021 Journal of Neuroscience, Kim et.al 2022 Nature, Zhou el.al 2022 Nature and Chen et.al 2025 PNAS) and clarified that our mice did not exhibit overt circadian abnormalities, as evidenced by comparable ambulatory activity patterns between wild-type vs NPY-neuron specific SIK3 knockout or placebo vs GLPG3970 treated mice across both the light and dark cycle (Lines: 618-629). This suggests that the metabolic phenotype observed in our model is not secondary to altered sleep-wake cycle or circadian rhythm but rather reflects a direct role of SIK3 in NPY neuron-mediated energy homeostasis.*

2. Based on the sn-RNA-seq data, Sik3 is expressed in neurons, oligodendrocytes, and tanycytes. To enhance clarity, Figure 1G can include a bar chart illustrating the proportion of double-positive cells (e.g., SIK3+/NPY+ or SIK3+/POMC+) relative to total SIK3+ cells.

*Our response: We thank the reviewer for this constructive suggestion. In line with the reviewer's recommendation, we have now quantified and included the proportion of double-positive cells (SIK3$^+$/NPY$^+$ and SIK3$^+$/POMC$^+$) relative to total SIK3$^+$ cells. Our analysis shows that approximately 24% of hypothalamic SIK3$^+$ cells co-express NPY and about ~18% co-express POMC, supporting the notion that SIK3 is enriched in key neurons regulating energy homeostasis. This additional analysis has been incorporated into the revised figure (Line:167-169, Supplemental Figure S1E).*

3. The use of 12 subtitles might be excessive; consider merging some to enhance reader friendliness. Additionally, the sections proposing CRTC1 and HDAC3 as downstream targets could be repositioned later in the text to improve logical flow. Currently, these sections appear disconnected from the preceding and subsequent results, which may hinder readability.

*Our response: We agree that consolidating some subtitles improves readability and have accordingly merged several related sections to streamline the result section into 9 subtitles. If necessary, we would be happy to further consolidate or reduce the text after acceptance to comply with Nature Communication's journal guidelines. We have retained the current placement of the sections discussing CRTC1 and HDAC5 as downstream targets, as this positioning allows the mechanistic findings to directly follow the molecular signalling data that led to their identification as key downstream effectors responsible for the observed phenotypic changes. We believe this order preserves the logical flow of our experimental narrative and more effectively connects these pathways to the SIK3 signalling context.*

4. The assertion that "SIK3 centrally regulates leptin and insulin signaling through phosphorylation and inhibition of CRTC1 and HDAC5" (Line 413) is considered to be tuned down or proper discussion is needed to clarify this point, as the current data do not directly establish this regulatory mechanism.

*Our response: We thank the reviewer for this comment. Previous studies in CRTC1 and HDAC5 knockout mice have demonstrated obese phenotypes associated with impaired insulin and leptin signalling. Given that CRTC1 and HDAC5 are the predominant isoforms expressed in the hypothalamus and are well-established downstream substrates of SIK3, our in vitro and in vivo findings are consistent with a model in which SIK3 modulates energy balance through these pathways, aligning with our observation that loss of SIK3 in NPY neurons enhances central leptin and insulin signalling. We hypothesize that this relationship links upstream SIK3 and hormonal signalling to downstream metabolic outcomes. However, we agree with the reviewer that further studies are required to establish the direct regulatory mechanism by which SIK3 centrally modulates leptin and insulin signalling. We have therefore revised the relevant statements in the manuscript to temper our conclusions, using terms such as "associated with" or "indirectly regulates," (Lines: 443-445) and have clarified that additional studies are needed to determine how SIK3 directly regulates these signalling pathways in the Discussion section (Lines: 681-684).*

**Following are some minor points:**

1. The bars of Sik1 and Sik2 under the chow diet in Fig 1H seem to be lower than 1. How is the data normalized should be clarified. Also, the housekeeping gene used for normalization in qPCR (Gapdh or ppia) should be specified in legends and explained in Methods.

*Our response: We apologize for this minor oversight. The qPCR data in Figures 1H and 1I are normalized to the housekeeping gene Gapdh and expressed as fold change relative to the average expression under chow diet for each individual gene (i.e., SIK1, SIK2, or SIK3). We have clarified this in the figure legends, updated the Methods section to specify the use of Gapdh, and revised the relative fold values in Figure 1H to ensure accuracy (Figure legend Lines: 814-818).*

2. The data presentation in Figs 1L and 1N can be improved. The significance of the dashed lines and inset are unclear. Some of the axes of the bar charts can be better labeled (for example, Fig 3I). The significance of the yellow and white arrows in Figs 6M and 6P should be explained in the legend.

*Our response: We thank the reviewer for these helpful suggestions. We have revised Figures 1L and 1N to clarify the dashed lines and added "ARC" to indicate the arcuate nucleus. The insets are to provide colour images highlighting immunostaining using green fluorescence. Figure 3I has been updated with clearer x-axis labeling to improve data presentation. In Figures 6M and 6P, the legends have been revised to explain the significance of the yellow and white arrows. These changes enhance clarity and ensure that all annotations and data features are clearly interpretable.*

3. The legend of Fig 5 seems overly brief. The time of re-fed (Fig 5B) and how is glucose administered in the glucose tolerance test (5F) should be specified in the legends.

*Our response: The figure legends for Figure 5 have been revised to provide additional details. Specifically, the refeeding duration for Figure 5B and the route of glucose administration in the glucose tolerance test for Figure 5F have now been clearly specified in the legends. (Lines: 864-868)*

4. The abbreviation SNS can be explained around Line 300.

*Our response: The abbreviation "SNS" has now been defined as "sympathetic nervous system" at the first mention in the manuscript (Line 308).*

5. The statement that "This study provides the first evidence that hypothalamic SIK3 signaling plays a key role in promoting development" (Line 511) is not accurate and should be revised.

*Our response: We have now revised the statement to more accurately reflect our findings. The sentence now reads: "This study provides evidence that hypothalamic SIK3 signaling regulates energy homeostasis and may contribute to the development of obesity" (Line 571-573).*

**Detailed response to Reviewer 2's comments:**

Reviewer #2 (Remarks to the Author):

The manuscript provides significant mechanistic insight into the role of enhanced SIK3 level/activity in AgRP/NpY neurons in the development of HFD induced obesity using neuron specific SIK3 knockout mouse model and clinical candidate inhibitor studies with DIO mice. The authors' propose mechanism for the elevated SIK3 activity in orexigenic NpY neurons of HFD fed animals is based on the cytoplasmic sequestration of SIK3's effector proteins CRTC1 and HDAC4 downstream of impaired insulin and leptin signaling in mediating the observed obesity phenotype. However, the causal links between these pathways are primarily correlative and gathered from different experimental settings. In this vein the reported decrease in NpY level of NpY SIK3 knockout neurons although related to these pathways no mechanistic detail was provided. The study is timely and relevant, especially in light of recent findings showing anti-obesogenic effects of pan-SIK inhibition (e.g., [DOI:10.1161/res.137.suppl_1.Wed084]). While that abstract confirmed the therapeutic potential of SIK inhibition, it did not identify specific isoforms or mechanisms. This manuscript addresses that gap by targeted elimination of SIK3 in orexigenic NpY neurons and SIK2 in POMC neurons respectively albeit to lesser extent.

*Our response: We thank the reviewer for this thoughtful and balanced assessment of our study. Given the complexity of nutrient and hormonal signalling in vivo, mechanistic insight is most effectively gained through the integration of complementary experimental approaches. In this context, our genetic, pharmacological and signalling data collectively provide strong evidence linking SIK3 activity, insulin and leptin signalling, and downstream transcriptional regulators to the observed metabolic phenotype. Our intention was to present an integrated model in which enhanced SIK3 levels in orexigenic AgRP/NPY neurons contribute to diet-induced obesity through coordinated regulation of CRTC1 and HDAC5 localisation in the setting of impaired central insulin and leptin signalling and to show that pharmacological inhibition improves metabolic health. Collectively, the data provide a coherent and biologically plausible framework supporting this model.*

*With respect to the reduced NPY levels observed in SIK3-deficient neurons, we agree that additional mechanistic resolution would be valuable and we view this as an important avenue for follow-up studies arising from the present work. More broadly, we note that fully delineating complex biological pathways typically requires multiple complementary studies rather than a single manuscript. By way of example, the role of SIK3 in sleep physiology was ultimately resolved across at least four separate Nature papers (Funato et al., 2016 Nature; Wang et al., 2018 Nature; Kim et al., 2022 Nature; Zhou et al., 2022 Nature), each contributing a distinct dimension to the overall understanding. In a similar vein, the present study establishes a strong foundation for understanding the role of hypothalamic SIK3 in body weight and metabolic regulation, upon which future work can build to address additional mechanistic layers beyond the scope of this manuscript.*

1. Although the initial version holds SIK3 elimination in AgRP\NpY neurons responsible for the improved metabolic phenotype, the revised manuscript appropriately acknowledges

possible contributions from SIK2 and SIK3 from other neuronal and none neuronal cells of the brain not excluding the peripheral metabolic tissues that expresses either gene alone or in combination. This balanced interpretation is commendable.

*Our response: We thank the reviewer for their positive feedback and are pleased that the balanced interpretation in the revised manuscript is appreciated.*

2. Experimental Limitation: Lack of SIK3 Rescue The authors restored NPY expression in NPY-neuron-specific SIK3 KO mice using a Cre-dependent AAV construct, which partially rescued the phenotype. However, a more definitive approach could have addressed the restoration of SIK3 itself in NpY neurons of NPYCre/+;SIK3lox/lox mice. Such an experiment would strengthen the claim that SIK3 absence/inhibition in these neurons is the sole and predominant member responsible for the observed metabolic improvements.

*Our response: While we agree that restoring SIK3 expression specifically in NPY neurons would provide additional confirmation, this experiment is currently constrained by well-established technical limitations of AAV-mediated gene delivery. The full-length SIK3 coding sequence (~4.1 kb) exceeds the practical packaging capacity of standard AAV vectors (~4.7 kb) once promoter, reporter and regulatory elements are included. We have attempted to generate an AAV-based SIK3 construct; however, these efforts were unsuccessful due to the inherent size constraints of the SIK3 coding sequence. Although dual-AAV strategies (e.g. homologous recombination–based approach) have been reported, these methods introduce substantial variability in recombination efficiency, expression levels, and cell-type specificity, particularly in hypothalamic neurons. As a result, outcomes can be difficult to interpret and may introduce additional confounds. For this reason, we elected not to pursue a technically marginal rescue that could generate ambiguous results. Instead, we performed a functional rescue by restoring NPY expression in NPY-neuron–specific SIK3 knockout mice, which fully rescued the metabolic phenotype. This places SIK3 upstream of NPY in a linear pathway within these neurons and strongly supports our conclusion that SIK3 activity in NPY neurons is a predominant driver of the observed effects.*

3. in discussion (lines 726-728) authors stated that 'the absence of obesity phenotypes in whole-body SIK2 knockout mice under HFD feeding suggests that SIK2 deficiency alone is insufficient to drive substantial weight loss' However from the statement here one can only drive the conclusion that systemic absence of SIK2 mice are resistant to HFD induced obesity and almost inacceptable to say SIK2'a role in weight loss. Based on the literature nobody has tested SIK2 specific or PAN SIK inhibitor on already obese mice except this study and the recent abstract mentioned above. Since SIK3 inhibitor used in this study targets both SIK2 and SIK3 we can only conclude that both gene have different but finite responsibility for the development of obesity in DIO mouse model (and genetic leptin deficient model).

*Our response: We appreciate this insightful comment and have revised the wording to more accurately reflect SIK2's contribution while emphasizing the distinct and complementary roles of SIK2 and SIK3. Specifically, whole-body SIK2 knockout mice show resistance to HFD-induced obesity, but this does not directly indicate a role in driving weight loss. Importantly, our study, together with recent pharmacological data using the pan-SIK inhibitor that also targets SIK2, supports the conclusion that both genes contribute to the development of obesity, each with a finite but*

*meaningful impact. Accordingly, the Discussion (lines 721–728) has been revised to read as follows: "Given the modest ~2-fold selectivity of GLPG3970 for SIK3 over SIK2, inhibition of SIK2 may also contribute to the observed improvements in diet-induced obesity and glycemic control. While whole-body SIK2 knockout mice show resistance to diet-induced obesity, this does not directly indicate a role in driving weight loss. Together, these findings highlight the complementary contributions of SIK2 and SIK3 and underscore the need for future studies using selective inhibitors or SIK2 or combined SIK2/SIK3 deletion models to define the roles of these kinases in mediating the therapeutic actions of GLPG3970."*

4. Species Differences The manuscript notes that SIK1–3 expression/activity patterns in mouse metabolic tissues, particularly the liver, differ from human equivalents. This point warrants a precaution to be mentioned in discussion regarding translational relevance and potential limitations for human application. Overall, this manuscript makes a valuable contribution to understanding the role of NpY specific SIK3 in energy homeostasis and obesity. Addressing the above points would further strengthen its impact and translational significance.

*Our response: We thank the reviewer for highlighting this important point. We agree that species-specific differences in SIK1–3 expression and activity could influence the translational relevance of our findings. While SIK3 is highly conserved between mouse and human, the precise tissue- and cell-type expression patterns may differ. We have therefore added a note in the Discussion acknowledging this limitation and cautioning that extrapolation to human physiology should be made carefully. Notably, the GLPG3970 compound studied here has also been tested in clinical trials in humans, providing preliminary evidence that SIK inhibition is pharmacologically active across species. Accordingly, the Discussion (lines 728-731) has been added to read as follows: "While SIK3 is highly conserved between mouse and human, species- and tissue-specific differences in SIK isoform expression pattern, particularly in metabolic tissues, may affect translational relevance." We appreciate the reviewer's suggestion, which we believe strengthens the overall impact and translational context of the manuscript.*

**Detailed response to Reviewer 2's comments:**

Reviewer #2 (Remarks to the Author):

The authors have addressed the methodological and conceptual concerns raised in my previous review. The revisions are sufficient to meet the standards for publication, though the overall contribution remains on the threshold of acceptance.

*Our response: We sincerely thank the reviewer for their constructive feedback and thoughtful evaluation of our work. We are very pleased that the methodological and conceptual concerns have been addressed to the reviewer's satisfaction, and we are glad that the reviewer considers the manuscript suitable for publication. We would like to further emphasize the translational significance of our study, as one of the SIK3 inhibitors tested in this manuscript has previously progressed to a Phase 2 clinical trial, supporting the notion that SIK3 represents a viable and clinically relevant therapeutic target.*
